# HBEGF-TNF induce a complex outer retinal pathology with photoreceptor cell extrusion in human organoids

**DOI:** 10.1038/s41467-022-33848-y

**Published:** 2022-10-19

**Authors:** Manuela Völkner, Felix Wagner, Lisa Maria Steinheuer, Madalena Carido, Thomas Kurth, Ali Yazbeck, Jana Schor, Stephanie Wieneke, Lynn J. A. Ebner, Claudia Del Toro Runzer, David Taborsky, Katja Zoschke, Marlen Vogt, Sebastian Canzler, Andreas Hermann, Shahryar Khattak, Jörg Hackermüller, Mike O. Karl

**Affiliations:** 1 Center for Regenerative Therapies Dresden (CRTD), Technische Universität Dresden, Dresden, Germany; 2 German Center for Neurodegenerative Diseases (DZNE) Dresden, Dresden, Germany; 3 Department Computational Biology, Helmholtz Centre for Environmental Research—UFZ, Leipzig, Germany; 4 Technische Universität Dresden, Center for Molecular and Cellular Bioengineering (CMCB), Technology Platform Core Facility Electron Microscopy and Histology, Dresden, Germany; 5 Department of Neurology, Technische Universität Dresden, 01307 Dresden, Germany; 6 Department of Computer Science, Leipzig University, Leipzig, Germany

**Keywords:** Neurological models, Neurodegeneration, Stem-cell research, Experimental models of disease, Preclinical research

## Abstract

Human organoids could facilitate research of complex and currently incurable neuropathologies, such as age-related macular degeneration (AMD) which causes blindness. Here, we establish a human retinal organoid system reproducing several parameters of the human retina, including some within the macula, to model a complex combination of photoreceptor and glial pathologies. We show that combined application of TNF and HBEGF, factors associated with neuropathologies, is sufficient to induce photoreceptor degeneration, glial pathologies, dyslamination, and scar formation: These develop simultaneously and progressively as one complex phenotype. Histologic, transcriptome, live-imaging, and mechanistic studies reveal a previously unknown pathomechanism: Photoreceptor neurodegeneration via cell extrusion. This could be relevant for aging, AMD, and some inherited diseases. Pharmacological inhibitors of the mechanosensor PIEZO1, MAPK, and actomyosin each avert pathogenesis; a PIEZO1 activator induces photoreceptor extrusion. Our model offers mechanistic insights, hypotheses for neuropathologies, and it could be used to develop therapies to prevent vision loss or to regenerate the retina in patients suffering from AMD and other diseases.

## Abstract (web summary)

Complex neuropathologies, like macular degeneration, are common but incompletely understood. Here the authors report a complex human retina pathology organoid model induced by HBEGF-TNF, causing PIEZO1-mediated photoreceptor degeneration by cell extrusion.

## Introduction

Neurodegenerative diseases which cause vision loss present a huge public and personal health burden. Age-related macular degeneration (AMD) involves complex risk factors^1–3^ and is part of a group of macular degenerative diseases (MDDs); inherited retinal dystrophies (IRDs) are caused by many mutations with variable phenotypes^4,5^. Animal models are advancing our understanding, but most animals lack a macula^6^ and only show some traits of AMD/MDDs^2,4^. Specifically, the complex retinal phenotypes in advanced AMD (geographic atrophy)^7–14^ and some MDDs/IRDs^15–17^ are still unsolved: data suggest that photoreceptor (PR) degeneration, which may be caused or followed by atrophy of the opposing retinal pigment epithelium (RPE) and choroid^8,9,11^, develop in combination with Müller glia (MG) pathologies^7,10–13,18–28^, particularly scarring^10,19–22,24^, and retinal remodeling^26,28,29^, (transient) thickening^10,15–17,30^, and dyslamination^7,10,26,28^. Conversely, in other PR pathologies, retinal thickness just decreases, and scars and remodeling predominately occur at endstages^5,23,26,31,32^. Together, effective therapy development is still a challenge; targeting pathologic processes to prevent pathology progression is a future potential treatment option for a broad patient spectrum^2–4^. Human retinal organoids (HROs) generated from human induced pluripotent stem cells (hiPSCs) provide a reduced model system that will facilitate therapy development. HROs reproduce some key aspects of retinal development and structure^33–37^, but physiological and synaptic functions are still limited. Neurodegenerations may take decades to develop in patients, and it is still unclear how to reproduce and accelerate pathogenesis in human models. Developmental disorders, and some changes of IRDs have been modeled^38^: However, to what extent dynamic (onset to completion) and complex pathogenesis can be reproduced in postmitotic HROs remains uncertain. Here, we sought to approach this by developing an inducible phenotypic model for complex pathologies, and explore if HROs provide mechanistic access. TNF (tumor necrosis factor) is a proinflammatory factor regulating different cell death types and may be involved in PR degeneration^23,39–41^. Further, EGF (epidermal growth factor) and HBEGF (heparin-binding EGF-like growth factor), two EGF-family members, and TNF regulate glial pathologic processes^23,31,42–44^. Thus, we hypothesized that the combined application of TNF and HBEGF to postmitotic HROs generated from hiPSCs of healthy donors might be sufficient to induce PRs and MG pathologies as one complex phenotype. TNF/EGF signaling is also interesting beyond this: Both factors regulate inflammation and regeneration^39–41,44^. Among many candidates, TNF and EGF are predicted AMD risk genes^1,45,46^ and progression factors^47,48^, and while some studies suggest elevated gene/protein levels in AMD and other pathologies^39–42,44,48,49^, none of this has been validated yet in larger patient studies or is considered a high priority (Supplementary Data 1). Overall, the differential functions^50^ and pathogenicity of TNF-/EGF-signaling in neurodegenerations are still unknown.

In this work, we show that combined (not separate) application of HBEGF and TNF (HT) in a human model system is sufficient to induce a complex pathology termed the HT-HRO model: PR degeneration and MG pathologies develop simultaneously as one dynamic phenotype (progressing from onset to endstage). It is highly effective: Inducible, dose-dependent, and reproducible. We demonstrate that our established HRO system is cone-rich and provides many parameters of the human retina. Notably, it uniformly and robustly reproduces some anatomical parameters of the foveal–parafoveal subregion of the macula. Through (ultra)structural, transcriptome, and mechanistic analyses, we gain insight into the HT-HRO model’s complexity and identify genes/signaling pathway candidates regulating pathogenesis—some associated with retinal diseases, and some new ones, like the mechanosensor PIEZO1. Thereby, we reveal a pathomechanism: photoreceptor degeneration by cell extrusion. Extrusion might be a cause of displaced/ectopic PRs described upon aging^29,51–54^, and in patients with AMD^10,12,13,18,29,51,52,54^ and other pathologies such as inherited retinal dystrophies due to CRB1^16^ and RPGR^55,56^ gene mutations. Our studies with pharmacologicals in HROs suggest that inflammatory (HBEGF-TNF) and biomechanical (PIEZO1) factors might induce and/or exacerbate neuropathologies in patients, and represent untapped therapeutic targets to prevent not just PR extrusion, but even a complex neuropathology. Overall, our results support a versatile preclinical human pathology model that will advance research on AMD and other pathologies.

## Results

### Human retinal organoids provide prerequisites for pathology modeling

**Fig. 1:**
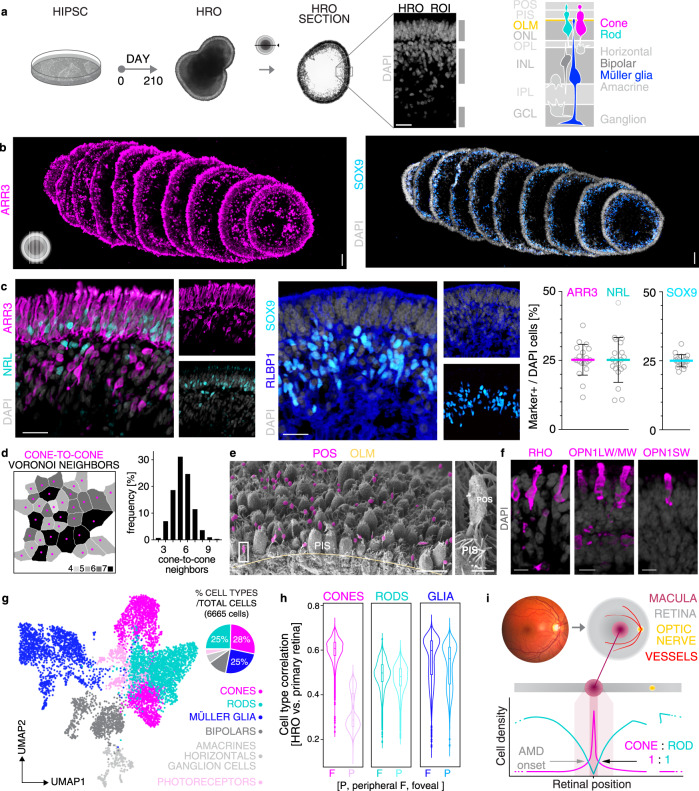
HRO system reproducing several characteristics of the human retina. **a** Schematic. HRO generation, retinal structure, and cell types quantified on (**b**, **c**) immunostained serial HRO sections: cones (ARR3); rods (NRL); MG (SOX9, RLBP1). **c** Graphs: circles represent individual HROs (*n*) derived from *N* = 4 independent experiments, *n* ≥ 5/*N*; mean ± SD over *n*. Part of the dataset presented in Fig. 3c, Supplementary Data 2. **d** Voronoi neighbor analysis: number of cone neighbors analyzed from en-face images of HRO wholemounts immunostained for cone (ARR3), photoreceptor (RCVRN), and Müller glia (SLC1A3 + RLBP1) markers (Supplementary Fig. 3a–e, k; *N* = 1; *n* = 8; mean ± SD over *n*; 3 ROIs averaged/*n*). **e** Scanning electron microscopy of HRO indicated the formation of photoreceptor inner (PIS) and outer (POS) segments (*N* = 1, *n* = 6). **f** Rod (RHO) and cone (OPN1LW/MW, OPN1SW) opsin immunostaining on HRO sections (*N* = 1; *n* = 9). **g** HRO single-cell RNA-seq analysis: dotplot shows cell clusters after Louvain cluster detection in a UMAP embedding. Piechart: relative abundance of major cell types in an individual HRO. Dotplot/piechart: pseudocolored for cell types as indicated. Photoreceptors (light pink): likely immature cones, but could not be clearly assigned. **h** Comparison of single-cell RNA-seq data: distribution of Pearson correlation of HRO cells and human retinal fovea and periphery reference datasets^58^. Violin graph: Pearson correlation of cones, rods, and Müller glia in HRO (*N* = 1) against the human reference vectors (correlation values of three individual human donors (*N* = 3) combined in one violin graph; Supplementary Fig. 3g: individual donor data). Boxplots: minimum, 1st quantile, median, 3rd quantile, maximum values, dots are outlier data points (lower range: the 25th percentile-1.5 × IQR; upper range: 75th percentile + 1.5 × IQR; IQR: interquartile range). **i** The human anatomical macula: eye fundus image (at age 28) and schematic of the retina. Graph: macular cone–rod densities in humans in vivo (references: Supplementary Fig. 3i, j). Arrows: cone–rod ratio replicated in HROs; prevalent site of AMD/MDDs onset^9,110^, (Supplementary Fig. 3i, j). Scale bars: **b** 1 mm, **c** 50 µm, **e** 10 µm (inset 1 μm), **f** 10 µm. Source data are provided as a Source Data file.

We recently established an HRO system that we further characterized here using various hiPSC lines from healthy donors (Fig. 1a and Supplementary Fig. 1). At day (D) D200 of differentiation, the average HRO had an estimated surface area of approximately one-quarter of the human fovea, the central region of the macula (7 mm^2^, based on mean circumference of 2.3 mm)^6,25^, with low variance (Supplementary Fig. 2a). Immunostained HRO cross-sections showed a laminated structure with all major retinal cell types (Fig. 1b and Supplementary Fig. 2b–f): cone (ARR3) and rod (NRL) photoreceptors in the outer nuclear layer, MG (RLBP1, SOX9) and interneurons: bipolar (PRKCA, VSX2 + SOX9−), amacrine and horizontals (ELAVL3/4, PAX6), in the inner nuclear layer, and retinal ganglion cells (BRN3, PAX6). Histological analyses also indicate synaptic layer formation (Fig. 1a and Supplementary Fig. 2e, f) reminiscent of outer and inner plexiform layers in vivo^6^. Notably, cones, rods, and MG show a cell ratio of about 1:1:1, with on average 28%, 25%, and 25% of total cells, respectively (Fig. 1c; *n* HROs per *N* independent differentiations; *N* = 4; *n* ≥ 5/*N*, 2 hiPSC lines); with a comparable interorganoid (coefficient of variation (COV) for ARR3, NRL, and SOX9 marker+ cells/total cells (DAPI) per ROI: 10%, 17%, and 5%) and intraorganoid variance (COV: 18%, 20%, and 11%) (Supplementary Fig. 2b). The remaining 22% are other retinal cells. The human retina has a specific PR mosaic, and cell-pattern analysis of HROs using GPU-accelerated image processing and machine learning also identified two cell types with the same parameters as cones and rods, a 1:1 ratio, and a high cone density (27,000 ± 4782/mm^2^) (Fig. 1d and Supplementary Fig. 3a–e). Voronoi analysis showed that cones have on average 6±1 cone neighbors at 5 ± 1 μm distance. PRs also show characteristic morphologies with photoreceptor inner (PIS) and nascent outer (POS) segments located outside of the apical retinal border, called the outer-limiting membrane (OLM): ultrastructural studies and PRPH2 immunostaining support this (Fig. 1a, e and Supplementary Fig. 2g–k), and opsins are expressed in rods (RHO) and all cone subtypes: OPN1LW/MW cones predominate, whereas very few express OPN1SW (Fig. 1f, Supplementary Fig. 3f, and Supplementary Movie 1). When compared to ARR3 (pan-cone marker), opsins seem to not yet be fully upregulated in D200 HROs. Further, human retinal cells have defined gexotypes (gene expression histotypes) based on the anatomical region: CALB1 expressing cones are part of the peripheral but not macular human retina^57^, and are absent in HROs (Supplementary Fig. 2d). Comparative analysis of RNA-seq data from individual entire HROs (*n* = 6, D210, Supplementary Fig. 4a) with published human data showed that foveal genes are more enriched in HROs than those of the peripheral retina. HRO single-cell analysis by 10X genomics (Fig. 1g and Supplementary Fig. 4b–e, g) identified all major retinal cell types, and confirmed the cone, rod, and MG ratio (Fig. 1c). Pearson correlation analyses (Fig. 1h) revealed that HRO cones have a much higher median correlation (0.608) with foveal cones of the primary human retina^58^ than with peripheral human cones (0.287). MG correlate slightly more with the fovea (0.598 versus 0.561); and rods were found to be nearly the same (0.497 versus 0.481). However, this could not be explained by a mixture of more foveal- and more peripheral-related cells. Instead, the clear majority of rods and MG are either more foveal or show transcriptomes in-between foveal and peripheral cells (Supplementary Fig. 3g, h). The primary human macula data was derived from samples containing the entire foveola (cones only) and fovea, and parts of the parafovea (both with rods and cones), which might explain our correlation data. Together, this HRO system is cone-rich and uniformly reproduces several parameters, some of which are found at the interface of the fovea–parafovea of the human macula: a homogenous 1:1:1 cone, rod, and MG composition^6,25^ with low variance, a high cone density, a hexagonal cone pattern, and PR/MG gexotypes (Fig. 1i and Supplementary Fig. 3i–k). To determine to what extend it might reproduce a defined part of the retina requires studies of several other parameters. Here, we sought to apply this system, since it robustly reproduces many parameters advantageous for quantitative studies.

### HBEGF-TNF induces cone and rod degeneration in human retinal organoids

**Fig. 2:**
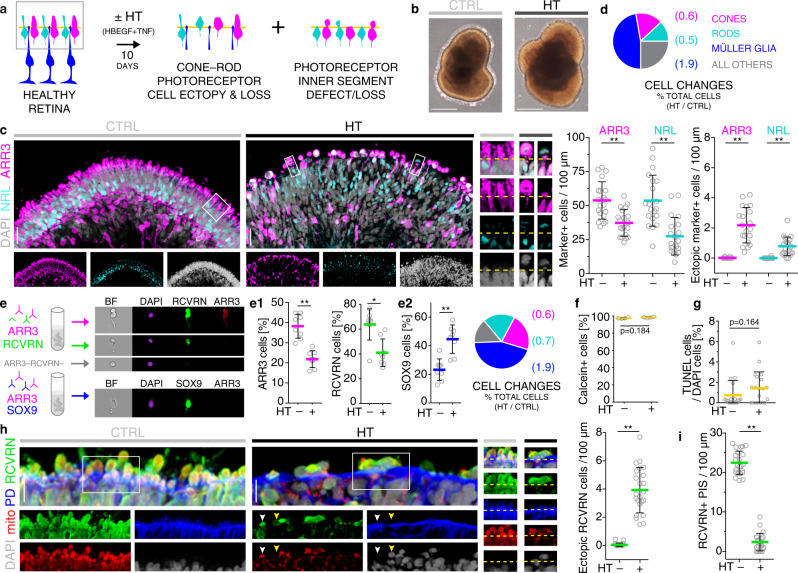
Combined HBEGF-TNF (HT) application induces photoreceptor degeneration in HROs. **a** Schematic: photoreceptor changes investigated. HT induces cone (ARR3) and rod (NRL) degeneration, including ectopic cell displacement above the outer-limiting membrane (OLM, indicated by a yellow dashed line) and cell loss. **b** Bright-field images: HT-treated and control HROs in culture. **c** Immunostained serial HRO sections were used to determine retinal cell-type composition: counts of cells positive for ARR3 and NRL in HT-HROs compared to controls (count of marker+ cells per 100 μm; Supplementary Data 2; control data partially presented in Fig. 1 c). **d** Summary: HT-induced changes in retinal cell-type composition (count of marker+ cells) per total cell number (nuclei, DAPI). Control data based on Fig. 1c: cone and rod; Fig. 4: MG data; compare to Fig. 1h; Supplementary Fig. 6g. Gray fraction: all other cells not labeled by the three markers used. **e** Cell composition analysis: flow cytometry of HT-treated and control HROs. Dissociated HRO cells were split into two fractions for analysis: immunostained for cone (ARR3) and photoreceptor (RCVRN) markers, or cone (ARR3) and MG (SOX9) markers. Images: imaging flow-cytometry analysis (DAPI, nuclei). **e1**, **e2** Graphs: flow-cytometry data. **e3** Piechart: HT-HRO cell composition. Numbers in parenthesis: fold change of HT-HROs compared to controls. Colors as depicted in (**d**). **f** Flow cytometry of Calcein live-dye staining, and (**g**) TUNEL staining on cryosections. **h**, **i** Immunostaining analysis of photoreceptor marker RCVRN on HRO serial sections: shows ectopic (extruded) photoreceptors outside of the OLM, and (**h**, **i**) pathologic changes and loss of RCVRN- and mitochondria-positive (mito) photoreceptor inner segments (PIS). Filamentous actin: visualized by Phalloidin488 (PD) to determine the OLM/apical boundary. **c**, **e**–**i** Graphs (mean ± SD) and statistics over *n*. Two-sided Student’s *t* test; **P < 0.0001, *P = 0.0023. **c**, **g**–**i** Graphs: each circle represents one HRO (*n*) derived from *N* = 4 with *n* ≥ 5/*N*; 2 hiPSC lines (Supplementary Data 2). **e** Graphs: each circle represents one set (s) of 6 − 9 pooled HROs (2 − 3 s/*N*, *N* = 3, 2 hiPSC lines). **f** Graphs: each circle represents cells analyzed from one sample (s); two pooled HROs/s; *N* = 1 independent experiment. **e**, **f** Mean ± SD and statistics over samples. Scale bars: **b** 500 µm, **c**, **h** 50 µm. Source data are provided as a Source Data file.

To test our hypothesis that HBEGF-TNF (HT) is sufficient to induce a pathology, we applied it once per day for 10 days to D200 HROs (HT-HRO), unless indicated otherwise (Fig. 2a and Supplementary Fig. 5a; *N* = 4, *n* ≥ 5/*N*). Phase-contrast microscopy of living HROs clearly showed that retinal epithelial thickness increased within days of HT treatment (Fig. 2b and Supplementary Fig. 5b, c). To assess cellular changes, we counted cell-specific markers on immunostained serial HRO sections (Fig. 2c, d): rods (NRL) and cones (ARR3) were significantly reduced by 49% and 31%, respectively (each *P* < 0.0001, Supplementary Data 2). Flow cytometry of two parallel marker sets confirmed this (Fig. 2e and Supplementary Fig. 6; *N* = 3, 2 − 3 HROs pooled/*N*; 2 hiPSC lines): in controls, 64 ± 4% of cells were PRs (RCVRN), with the larger fraction of them cones (38 ± 2% ARR3), and 23 ± 3% MG (SOX9). After HT treatment, 36% of all PRs were lost (*P* = 0.0018): 43% of cones (*P* < 0.0001) and 25% of rods (RCVRN minus ARR3), whereas MG doubled in number (*P* = 0.003). Notably, flow cytometry and TUNEL assay indicated intact cell membranes and high cell viability in controls and HT-HROs (Fig. 2f, g and Supplementary Figs. 6h and 7), raising the question of how PRs were lost. We noticed that a significant number of cones and rods (including cell nuclei) were found outside of the OLM (Fig. 2c, h and Supplementary Fig. 5d, e). Such pathologically displaced (ectopic) PRs have been recorded by patient histology and are thought to be more common in older patients^29,51–54^, AMD^10,12,13,18,29,51,52,54^, some MDDs/IRDs^15,16,32,56,59,60^, and pathologic extracellular deposits^13,61^; they have also been recorded by in vivo imaging^62^. However, the underlying mechanism and function, e.g., to protect or remove damaged/dysfunctional PRs, have not yet been studied. In other organs, mechanisms for cell displacement out of epithelia have been identified, termed cell extrusion: TNF, inflammation, or cell death/damage may be inducers^63,64^.

### Photoreceptor degeneration involves cellular defects and cell extrusion

**Fig. 3:**
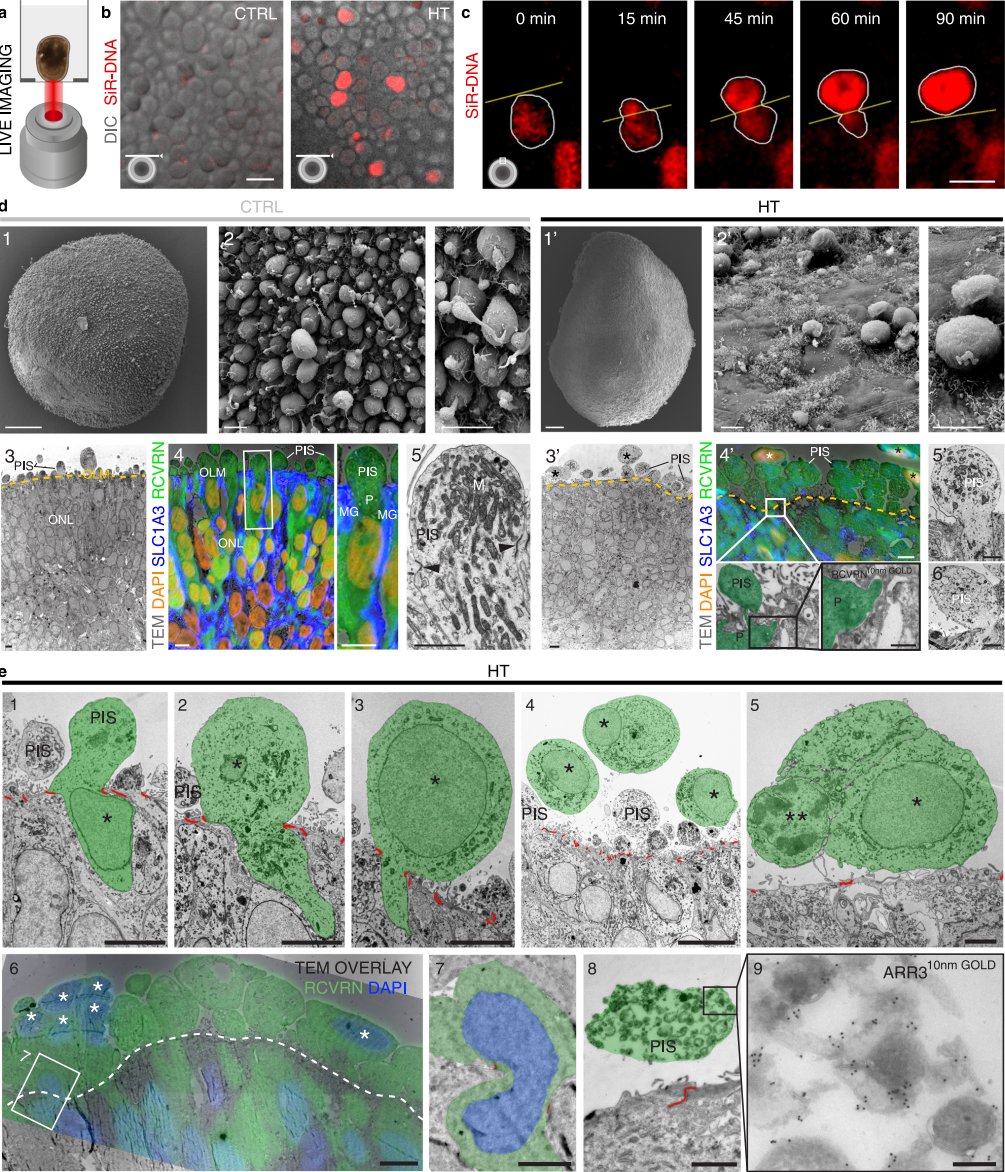
HBEGF-TNF treatment induces photoreceptor cell extrusion and photoreceptor inner segment (PIS) defects. **a** Live-imaging of HROs. **b** Differential interference contrast. **c** Spinning disk confocal microscopy. **b**, **c** HT-HROs and controls (CTRL) live in culture (Supplementary Data 3 and Movies 2–4). SiR-DNA cell nuclei live-dye labeling (acutely applied for imaging). **b** En-face imaging: ectopic nuclei appeared after 5 days in HT-HRO on its surface, but not in controls (optical plane positioned at the PIS level). Independent experiments: *n* = 10 HROs (Supplementary Data 3). **c** Cross-sectional image series from Supplementary Movie 3: cell nuclei move out of the apical organoid boundary onto its surface (yellow line). Cell nuclei in the outer retina appear round in controls and before extrusion, and transiently became hourglass-like shaped during extrusion. Independent experiments of *n* = 8 HROs (Supplementary Data 3). **d** HT-HROs compared to CTRL analyzed by scanning (SEM) electron microscopy (EM) of (1−2, 1’−2’), transmission EM (TEM) of epoxy-embedded HROs (3, 5, 3’, 5’−6’), and correlative-light electron microscopy (CLEM) of ultrathin cryosections (4, 4’): RCVRN (photoreceptors, P), SLC1A3 (Müller glia, MG), and DAPI (extruded (*) cell nuclei) staining, visualized by immunofluorescence and gold particles (black dots, 10 nm gold). *N* = 1 independent experiment (*N*) with *n* = 3 (TEM) and *n* = 5 (SEM) HROs (*n*) analyzed. **e1–5** TEM image series: photoreceptors (P, green pseudocolor) during or after extrusion (10-days HT). Cell junctions (OLM): red labeled. Photoreceptor nuclei: asterisks. Double asterisk: a dead nucleus (**e5**). **e6–9** CLEM of HT-HROs (**e6**, **e7**: 10-days HT; **e8**, **e9**: 20 days HT) immunolabeled for RCVRN (**e6, 7**) or ARR3 (**e8, 9**) and DAPI confirms that photoreceptors become extruded. **e6** Extruded cells (asterisks) and **e7** a photoreceptor in the process of being squeezed through the OLM, indicated by hourglass-shaped nucleus and position of cell junctions (white dashed line (**e6**), red pseudocolor (**e7**)), are visible. Boxed area: higher magnification in (**e7**). **e8, 9** PIS (green) of a cone (ARR3 labeled) in HT-HRO. **e** TEM and CLEM: each *N* = 1, *n* = 3 HROs/N. Mitochondria (M). Outer (ONL)/inner (INL) nuclear layer. Scale bars: **b** 25 µm, **c**, **e4**, **e6** 10 µm, **d1, 1’** 50 µm, **d2–4, 2’−4’**; ROI of **d2, 2’**; **e1–3, e5** 5 µm, **d5**, **5’−6’**; **e5, 7, 8** 2 µm, ROI of **d4’** 500 nm, **e9** 200 nm.

To determine whether PRs degenerate by regulated cell extrusion, and to confirm that this is not an artifact of tissue processing, we performed combined differential interference contrast and fluorescence en-face microscopy of living HROs once a day (Fig. 3a). This showed that cell nuclei (SiR-DNA labeled) appeared in large numbers on the outer HRO surface after 5–10 days of HT treatment but not in controls (Fig. 3b, *n* = 3 per variable). Confocal microscopy recordings over an average timeframe of 12 h on days 5–8 of HT treatment revealed a dynamic cell-extrusion process: cell nuclei frequently moved apically past the OLM and PIS region, and thus exited out of the retina onto the surface (*n* = 5 HROs; Supplementary Data 3 and Supplementary Movies 2–4). Occasionally, cell nuclei passing the OLM transiently took on an hourglass shape, suggesting that cells might be squeezing through the OLM to exit the retina (Fig. 3c at 15−60 min). Immunohistology (Fig. 2h, i and Supplementary Fig. 5d–g), and scanning (SEM, Fig. 3d1-2, d1’−2’), transmission (TEM, Fig. 3d3, 3’, 5, 5’, 6’, e1-5), and correlative-light (CLEM, Fig. 3d4-5, d4’, e6–9) electron microscopy provided further insight: in controls, PRs were positioned with their soma and nucleus in the ONL below the OLM and their PIS and POS above (Figs. 2h and 3d1-5 and Supplementary Figs. 2i, j and 5d, e), comparable to the retina in vivo. In HT-HROs, numerous PR soma and nuclei were pathologically ectopic of the OLM, and PIS numbers were reduced (Figs. 2h, i and 3d1’−4’ and Supplementary Fig. 5e–g) while the remaining PIS showed impaired mitochondria and structure (Fig. 3d5’−6’). Together, live and ultrastructural imaging show morphological changes supporting several stages of PR extrusion (Fig. 3d3’, 4’; e1-9) with their nuclei delaminating and passing through the OLM, indicated by hourglass-shaped PR nuclei (validated with RCVRN by CLEM; Fig. 3e6, e7). The nuclei and soma of extruded PRs localized apically of the OLM, and some attached to the HRO surface; most seemed phenotypically healthy (like controls), but some had altered chromatin morphology and margination, indicative of cell stress or death (Fig. 3e5). TUNEL assay confirmed this: about 1.5% of the extruded cones and rods were indeed dying, whereas most cells inside the retina were negative for different cell death assays (Fig. 2f, g and Supplementary Figs. 6h and 7). However, cell damage and death could still have been induced in situ and completed upon extrusion.

### HBEGF-TNF induces gliosis, retinal dyslamination, and scar formation

**Fig. 4:**
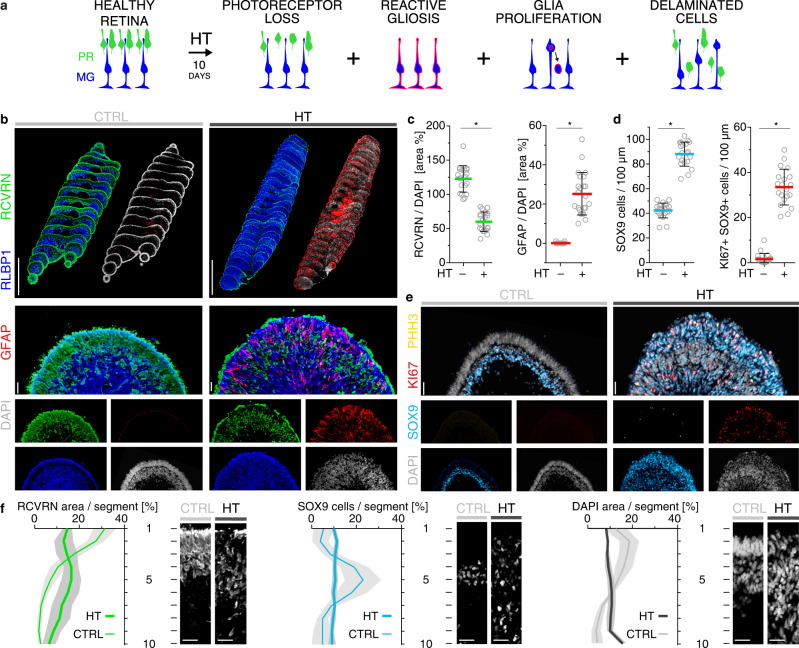
HBEGF-TNF induces reactive gliosis, glial proliferation, and retinal dyslamination in HRO. **a** Schematic of photoreceptor and glial pathologic changes (10-days HBEGF-TNF (HT) treatment). **b**–**e** Immunostained serial HRO sections were used to quantify the level of gliosis (GFAP) as well as the photoreceptor cells (RCVRN), Müller glia (RLBP1/SLC1A3 costain), cell nuclei (DAPI) based on marker area analysis, and proliferating (KI67), mitotic (PHH3, phosphohistone H3), and SOX9-positive (Müller glia) cells based on cell counts. Graphs: circles show individual HROs (*n*) with *n* ≥ 5 per independent experiment (*N*, *N* = 4). Two-sided Student’s *t* test; *P < 0.0001. **f** Cell delamination was quantified to describe retinal dyslamination in HT-HROs compared to controls. Based on *n* ≥ 5 HROs (*n*) per independent experiment (*N*, *N* = 4). **c**, **d**, **f** Graphs (mean ± SD) and statistics over *n*. Scale bars: **b**, **e** serial reconstructions 1 mm, others 50 µm. See Supplementary Data 2. Source data are provided as a Source Data file.

**Fig. 5:**
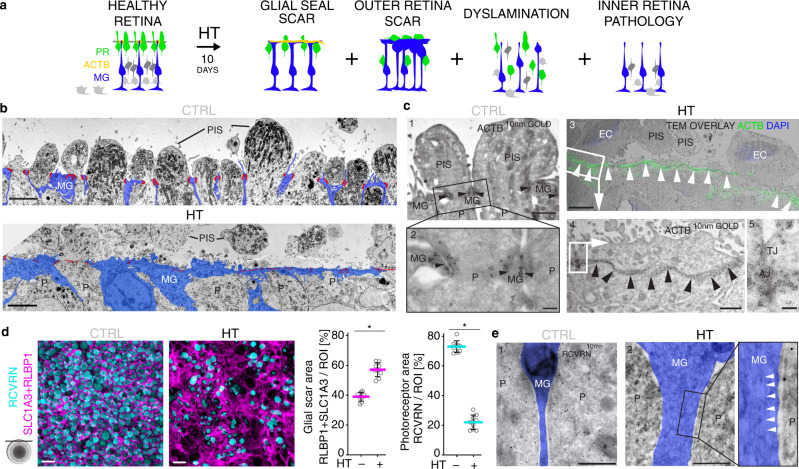
HBEGF-TNF-treated HROs show glial scar formation. **a** Schematic: retinal dyslamination and glial pathologic changes investigated (10-days HBEGF-TNF (HT) treatment). **b** Transmission electron microscopy of epoxy-embedded HRO sections with HT treatment/without (CTRL): images of the most apical HRO regions show photoreceptors (P) with inner segments (PIS) that contain mitochondria and are interconnected with Müller glia (MG). Pseudocolors: cell junctions (red lines), MG (blue). *N* = 1 independent experiment (*N*) with *n* = 3 HROs. **c** Correlative-light electron microscopy: in control HROs, ACTB immunostaining, visualized by gold particles (black dots, 10 nm gold), was restricted to cell junctions (**c1, 2**: arrowheads) of the OLM and apical microvilli of MG. Conversely, prominent electron-dense, filamentous structures spanning between cell junctions (arrowheads) were observed in HT-HRO: these were confirmed to be actin filaments (ACTB+, **c3–5**). Extruded cell nuclei (EC) stained with DAPI are frequently observed in HT-HROs, but not controls. *N* = 1 independent experiment (*N*) with *n* = 3 HROs. **d** En-face images of HRO wholemounts with/without HT treatment (10 days) immunostained for MG (SLC1A3 + RLBP1) and photoreceptor (RCVRN) markers. Quantitative analysis of marker area: MG expand at the apical surface level of HT-HROs while PIS diminish, indicative of glial scar formation. Graphs: circles represent individual HROs (*n*) derived from *N* = 1 independent experiments (*N*) with *n* = 9; 3 ROIs averaged/*n*; two-sided Student’s *t* test; *P < 0.0001. Graphs (mean ± SD) and statistics over *n*. **e** Tokuyasu cryosections stained with RCVRN (10 nm protein A gold) to identify photoreceptors (P). MG processes (RCVRN-negative, pseudocolored in blue) are thickened upon HT treatment, indicating hypertrophy (*N* = 1). Filaments (arrowheads) were observed in MG processes of HT-HRO. *N* = 1 independent experiment (*N*) with *n* = 3 HROs. TJ tight junction, AJ adherens junction. Scale bars: **b**, **c3** 5 µm, **c1**, **4** 1 µm, **c2**, **5** 200 nm, **d** 10 µm, **e1, 2** 500 nm. Source data are provided as a Source Data file.

Based on our hypothesis, HT might not only induce PR but also MG pathologies (Fig. 4a). This is also of interest because neighboring cells are mediators of cell extrusion in other organs^63,64^. Transcriptomics of human retinas and HROs suggest that HT receptors are physiologically expressed mostly in MG^36^ (Supplementary Fig. 4f, h). In HROs, HT induced expression of the glial fibrillary acidic protein (GFAP), a marker for reactive gliosis^23,25,43^, an umbrella term for various beneficial and detrimental functions upon neuropathologies, whereas GFAP was absent in controls (Fig. 4b, c and Supplementary Fig. 5f, g). Although PR numbers decreased by 50% after 10-day HT treatment (Fig. 2c, d), as confirmed by RCVRN immunostaining (Fig. 4b, c; *P* < 0.0001; Supplementary Fig. 5f, g and Supplementary Data 2), the average HRO size and total cell number rather increased slightly (Supplementary Fig. 5b, c, f). Quantification of cell-cycle (KI67), mitosis (phosphohistone-3) and MG (SOX9) markers indicated proliferative gliosis with duplication in MG number (Fig. 4d, e, Supplementary Fig. 8a–c, and Supplementary Data 2), closely compensating for rod/cone loss. We assessed further processes that might contribute to complex pathologies (Fig. 5a): retinal dyslamination was measured based on cell positions and radial (apical to basal) distributions on HRO sections using cell-specific markers. PRs (RCVRN), MG (SOX9) (Fig. 4f and Supplementary Fig. 8d, e), bipolar (VSX2), and amacrine (PAX6) cells (Supplementary Fig. 9a–f, h) are organized in layers in controls. All these cells became delaminated in HT-HROs, with PRs and MG equally redistributed radially across the epithelium (Fig. 4f and Supplementary Figs. 8e and 9d, e). After HT treatment, bipolars and amacrines also slightly decreased in number, and there was little evidence of synaptic connections (Supplementary Fig. 9d–g). (Ultra)structural studies showed that the outer retina is highly organized in controls: PRs are interspersed and interconnected with each other and with MG processes, and these cell connections make up the OLM (Fig. 5b). In HT-HROs, this order became severely disturbed: apical glial processes expanded in size, extended laterally along the retinal circumference, and replaced or covered PRs over large areas (Fig. 5b–d and Supplementary Fig. 10a, b), reminiscent of seal-like glial scars described in some complex pathologies and remodeling^10,19–22,24,26–28^. To quantitatively describe sealing by glial scars, we studied randomly sampled microscopic en-face images of immunostained wholemount HROs at the OLM level (Fig. 5d and Supplementary Fig. 10b): in controls, HRO surfaces were densely covered by PISs (73 ± 4% RCVRN+) surrounded by rather thin MG processes (39 ± 3% RLBP1 + SLC1A3+) at their base. In HT-HROs, there are fewer PISs (22 ± 5%, *n* = 9, *P* < 0.0001) and MG cover larger and irregularly shaped scar-like areas (57 ± 5%, *n* = 9, *P* < 0.0001). Analysis of HRO cross-sections indicated even larger MG scars in the outer retina that replace lost PRs and supersede the remaining ones (Supplementary Fig. 10c–f). The radial MG processes also appeared thicker, and their nuclei indented and multilobulated (Fig. 5e and Supplementary Fig. 10g), indicative of glial migration, hypertrophy, and scarring^27^. Flow-cytometry data support MG hypertrophy (Supplementary Fig. 10h, h1–3; *N* = 3, *n* = 8, *P* < 0.005). Actin condensation in other organs has been shown in scarring and in neighboring cells in cell extrusion^63^. In HT-HROs, glial scars have large, electron-dense, filamentous structures spanning between cell junctions, confirmed to be actin filaments by CLEM, whereas in controls, actin was restricted to cell junctions of the OLM and MG microvilli (Fig. 5c and Supplementary Fig. 10a). Together, HT induced several PR and MG pathologies which might reproduce features of remodeling and complex pathologies. Selected findings were confirmed in independent experiments (four hiPSC lines: Supplementary Fig. 11; three timepoints: Fig. 4 and Supplementary Figs. 5 and 8).

### Synchronous development of photoreceptor and glia pathologies

**Fig. 6:**
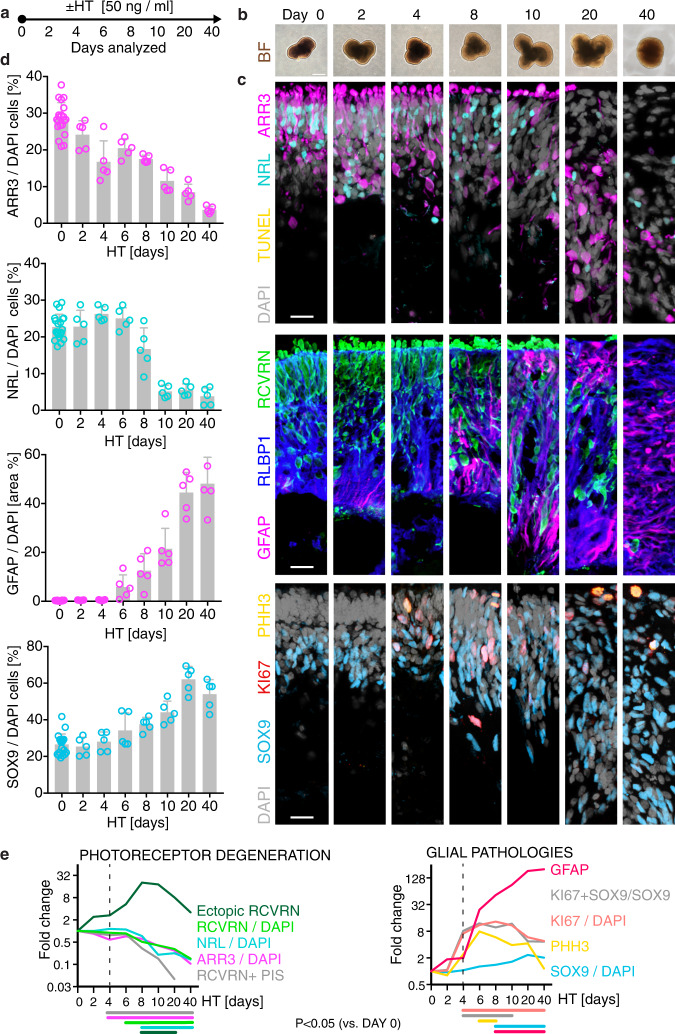
Kinetics of HBEGF-TNF-induced pathogenesis: simultaneous and temporally progressive photoreceptor and glial pathologies. **a** Experimental design: HBEGF-TNF (HT) was applied daily and HROs were analyzed at indicated timepoints. **b** Representative bright-field images of HT-treated HROs in culture. **c** Representative images and (**d**) quantitative analysis of photoreceptor and glial pathology development: cone (ARR3) and rod (NRL) photoreceptor (RCVRN) cell loss and cell ectopy, as well as loss of photoreceptor inner segments (PIS); gliosis (GFAP), cell proliferation (KI67, PHH3) of MG (SOX9), and total cell number (DAPI). See Supplementary Figs. 12 and 13. Graphs show mean ±SD, and circles represent individual HROs (*n*) derived from *N* = 1 independent experiment (*N*) with *n* ≥ 5 per variable. **b**, **c** *N* = 1 independent experiments (*N*) with *n* ≥ 5 HRO per timepoint. **e** Summary of quantitative data depicted in (**d**) and Supplementary Fig. 12. Summarized data are given as mean fold change (log2 scale) compared to control (0 days HT) HROs. Colored lines below the *x* axis depict statistical analysis using one-way ANOVA with Tukey post-hoc test (based on individual HROs (*n*)) at the indicated timepoints compared to control (day 0; statistics over *n*). Scale bars: **b** 500 µm, **c** 50 µm. Source data are provided as a Source Data file.

To determine if HT-induced PR and MG pathologies develop together, as often observed in complex pathologies, we studied the kinetics of pathogenesis. The markers described above were quantitatively analyzed at 0, 2, 4, 6, 8, 10, 20, and 40 days (D) of HT treatment (Fig. 6 and Supplementary Figs. 12 and 13): after D4, cones started to significantly decline in number and proliferative gliosis began. While cell death remained rare at all times, PIS loss and PR extrusion, together with PR loss and cell delamination, transiently increased from D4. PRs and MG gradually redistributed radially (Supplementary Fig. 12b–d), and MG number transiently increased (Fig. 6d). By D40, very few PRs remained. HT already induced a pathologic effect at 2.5 ng/ml, and the application of increasing concentrations up to 50 ng/ml showed that the magnitude of the pathology dose-dependently increased (Supplementary Fig. 14), thus, HT treatment was not just pathologic at a higher dose. Together, HT induced simultaneous onset and spatiotemporally synchronous, progressive, and stimulus-dependent development of PR and MG pathologies as one complex phenotype (Fig. 6e).

### Transcriptomics reveals potential mechanisms and disease associations

**Fig. 7:**
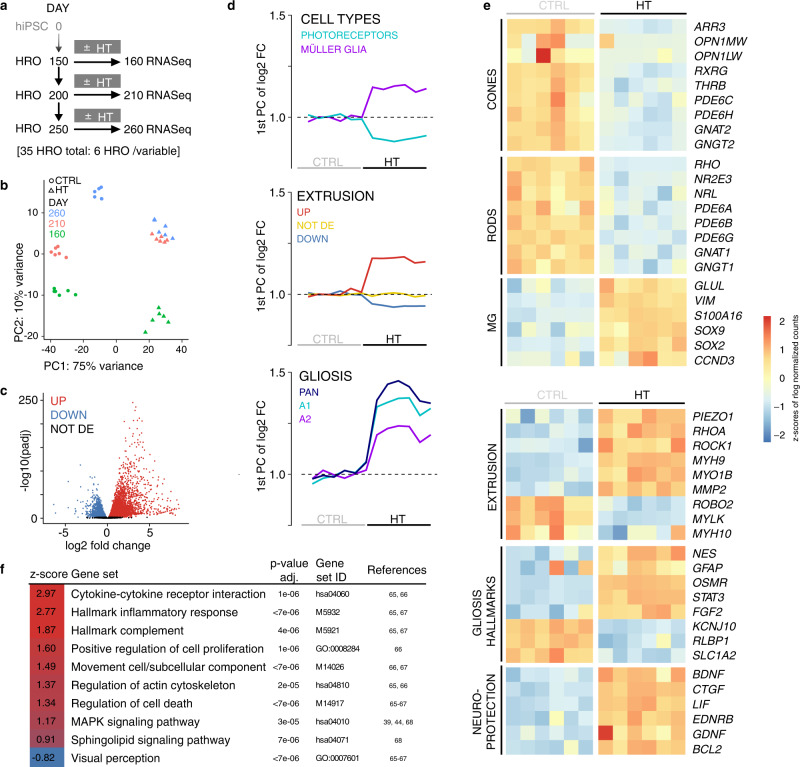
HT-HRO model transcriptomics indicates distinct pathologic processes and candidate regulators. **a** Experimental design: RNA-seq was performed on individual HROs after daily treatment with HBEGF-TNF (HT) for 10 days or with no treatment (CTRL), and at three different organoid ages. **b** Principal component (PC) analysis of the rlog-transformed count data. **c** Volcano plot shows statistical significance (−log10 of the adjusted *P* values) in the magnitude of change (log2FC values) of the differentially- (DEG; red and blue) and non-differentially (black) expressed genes between HT and CTRL (D210, FDR 0.01). **d** Graphs depicting the 1st PC of the log2-fold change in CTRL and HT-HROs (D210) for custom-made genes-of-interest (GOI) lists based on published data (Supplementary Data 4), representing retinal cell types (photoreceptors and Müller glia (MG)), cell-extrusion regulators, and gliosis (marker for pan-gliosis, A1 and A2 states). **e** Heatmaps depict *Z*-scores of rlog-transformed counts of genes expressed at D210 selected from the specified GOIs that are differentially expressed in HT versus CTRL HROs. **f** Ensemble of Gene Set Enrichment Analysis (EGSEA): selected examples of significantly enriched terms (e.g., biological functions or signaling pathways) in the HT-HRO model (Supplementary Data 5a) overlap with gene sets previously reported in relation to AMD (references indicated). The *Z*-score (red, up; blue, down) states how many standard deviations a given score deviates from the population’s mean. Data have been deposited on Gene Expression Omnibus.

To gain an insight into the pathomechanisms, we performed RNA-seq of control and HT-treated (10 days) HROs at different organoid ages: D150, D200, or D250 (*n* = 6 individual HROs/variable, Fig. 7a and Supplementary Fig. 15a–c). Principal component analysis (Fig. 7b) shows clearly separated variables, and high reproducibility (variance: HT treatment, 75%; age, 10%). We focused on D210, with 7829 differentially expressed genes (DEG) (FDR 0.01; Fig. 7c and Supplementary Fig. 15d–f). To determine changes in major cell types and selected pathologic processes, we assembled custom-made genes-of-interest (GOI) lists from literature (Supplementary Data 4, Fig. 7d, e, and Supplementary Fig. 15g): PR (41 of 42 GOIs) and MG (17 of 17 GOIs) genes become down- and upregulated, respectively. Cell-extrusion regulators reported for other organs showed 56 DEGs out of 78 total genes, and an increase in genes associated with gliosis (26 GOIs). Further gene expression patterns^64^ not yet studied in the human retina suggest gliosis programs with neurotoxic (10 GOIs) and neuroprotective (7 GOIs) functions. To assess biological processes and signaling cascades more systematically, we performed Ensemble of Gene Set Enrichment Analysis (EGSEA), resulting in 298 terms at D210 (Supplementary Data 5a), and 473 terms at all three ages (D160, D210, and D260; enrichment 0.6-fold to fivefold; adjusted *P* value <0.01). From these analyses, we found several terms or genes previously associated with retinal pathologies, and several new ones, which might provide access to still incompletely understood mechanisms, like cell extrusion and remodeling. For example, terms related to HT signaling, inflammation, cell contacts, migration, and epithelial mesenchymal transition (Supplementary Fig. 16). In the HT-HRO model, 19 out of 48 predicted AMD risk genes^2^ with predicted high priority (Supplementary Data 5b) were differentially expressed, and EGSEA revealed some potential AMD-related terms, including immune response^65^, complement^65^, and cholesterol homeostasis^2^ (Fig. 7f). Thus, we explored whether selected DEGs from the HT-HRO model are also differentially expressed in published patient datasets (Supplementary Data 5c). These datasets need to be considered carefully: most are based on few samples, and each might contain healthy to advanced lesion stages, and thus might also reflect secondary processes or changes in cell composition. Notably, some data show that *TNF*, *EGF*, *HBEGF*, and related receptors are enriched in advanced AMD retinas^65–68^ (Supplementary Data 1), whereas one with many samples does not^69^. Further, mechanosensor PIEZO1 regulates extrusion in other systems^63,64^, and increases in reactive glia^70^. *PIEZO1* and *2*, and other cell-extrusion-associated genes are enriched in HT-HRO (Fig. 7e) and potentially in advanced AMD retinas^68^. *PIEZO1* is expressed at low levels in cones of humans and HROs^36^ (Supplementary Fig. 4h). *PIEZO* mutations might cause MDDs^71^ and contribute to IRDs^72^. MAPK1 activation has been linked to PR death, glial proliferation, and possibly IRDs/AMD^39,44,68^. Sphingolipids are thought to be involved in PR cell death, OLM integrity, gliosis, and scarring, as well as cell extrusion in other organs^63^. Together, transcriptomics of the HT-HRO model supports our two hypotheses: HT-induced combined PR and MG pathology and PR degeneration via extrusion. It also revealed potential regulators.

### Pharmacologicals for potential cell-extrusion regulators prevent the complex pathology

**Fig. 8:**
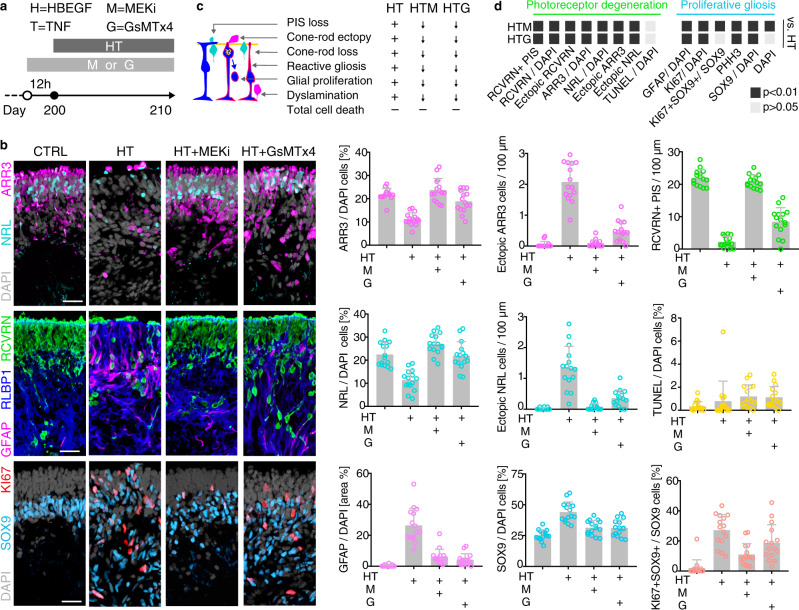
Pharmacological MEK or PIEZO1 inhibition attenuates photoreceptor and glial pathologies in the HT-HRO model. **a** Experimental design: starting from day 200, HROs were cultured for 10 days with HT treatment or without. A selective inhibitor (MEKi; U0126) of MAP kinase kinases (MEK1 and MEK2), known to inhibit MAPK activation, or GsMTx4, an inhibitor of PIEZO1 and other mechanosensitive ion channels, were added to HROs starting 12 h before HT application, and throughout the HT treatment period. **b** Representative ROI images recorded from immunostained serial HRO sections, and quantitative analysis to determine the effect of the pharmacological inhibitors on the HT-HRO phenotype, including retinal cell-type composition, pathologic changes of photoreceptor inner segments (PIS), cell death (TUNEL), glial proliferation, gliosis (GFAP), and retinal dyslamination (see Supplementary Fig. 17c). **c** Schematic summary of the inhibitor effects on the HT-induced phenotype; arrows indicate a reduction in phenotype severity. **d** Graphical depiction of statistical analysis of quantitative data shown in (**b**) Dark-gray squares depict significant changes (*P* < 0.01, one-way ANOVA with Tukey’s post hoc test) compared to HT-treated HROs. **c** Graphs: each circle represents 1 individual HRO (*n*) derived from *N* = 3 independent experiments (*n* ≥ 5/*N*). **c** Graphs (mean ± SD) and (**d**) statistics over *n*. Scale bars: **b** 50 μm. See Supplementary Data 6. Source data are provided as a Source Data file.

**Fig. 9:**
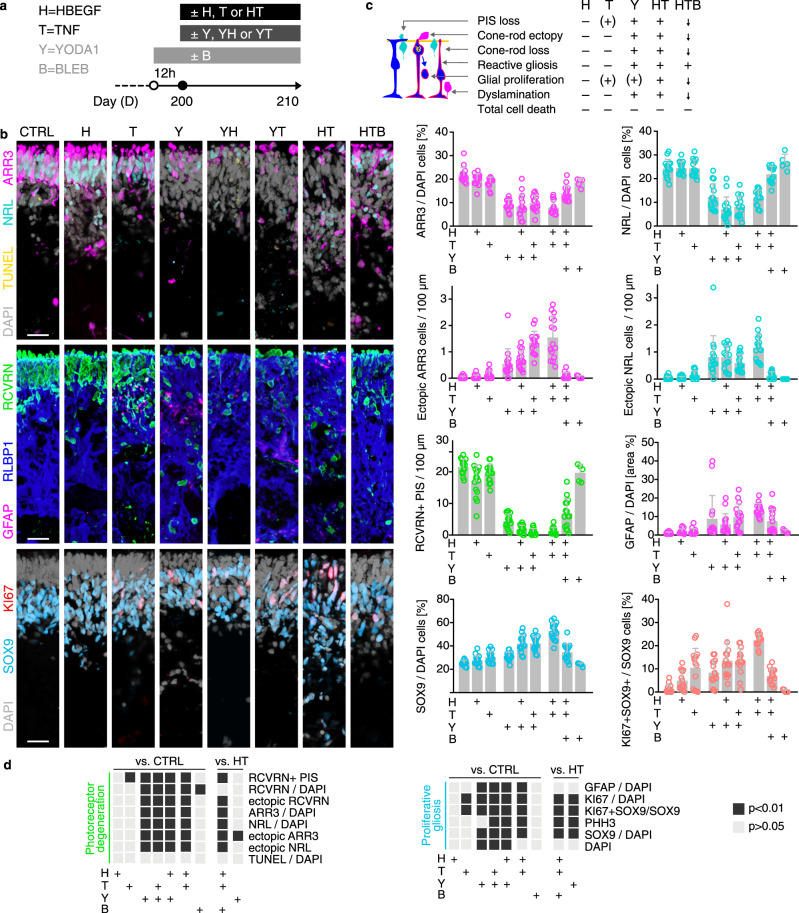
PIEZO1 activator YODA1 is sufficient to induce photoreceptor extrusion, but single HBEGF or TNF is not. **a** Experimental design: starting from day 200, HROs were treated with PIEZO1-channel activator YODA1 (Y) alone, or in combination with either HBEGF (H) or TNF (T). In addition, HROs were also treated with HBEGF or TNF alone, or in combination (HT). (−)-Blebbistatin (BLEB, B), an inhibitor of myosin II, was applied to some HT-treated HROs starting 12 h before HT application, and then throughout the HT treatment period. HROs were analyzed at D210. **b** Representative ROI images recorded from immunostained serial HRO sections and quantitatively analyzed to determine the effect of the treatments, including retinal cell-type composition, pathologic changes of photoreceptor inner segments (PIS), cell death (TUNEL), glial proliferation, gliosis (GFAP), and MG delamination (see Supplementary Fig. 18c). **c** Schematic summary of the effects of the treatments on HROs: treatments induced either none (–), a minor ((+)), or a major (+) phenotype, or a reduction (arrows) in phenotype severity compared to HT. **d** Graphical depiction of statistical analysis of quantitative data shown in (**b**). Dark-gray squares depict significant changes (*P* < 0.01, one-way ANOVA with Tukey’s post hoc test) compared to control (CTRL) or HT-treated HROs, respectively. **c** Graphs: each circle represents 1 individual HRO (*n*) derived from *N* = 3 independent experiments (*n* ≥ 5/*N*). **c** Graphs (mean ± SD) and (**d**) statistics over *n*. Types of treatments are indicated (+). Scale bars: **b** 50 μm. See Supplementary Data 6. Source data are provided as a Source Data file.

To probe pathomechanisms, we studied candidates revealed by transcriptomics: the contribution of PIEZO1 and MAPK to retinal homeostasis and pathology are unknown, and both might (differentially) regulate PR extrusion and glial pathologies. GsMTx4 (grammostola spatulata mechanotoxin 4) is a peptide originally identified in glia as a PIEZO1 inhibitor. To block MAPK activation, we used a chemical dual-inhibitor for MEK1/MEK2 (UO126 (MEKi)). Pharmacologicals were applied for 12 h prior to and 10 days during HT treatment (Fig. 8a and Supplementary Data 6). Both inhibitors each visibly reduced HT-induced retinal thickening (Supplementary Fig. 17a). Based on histology and quantitative analysis, HROs are morphologically more similar to controls than to HT-HROs, (Fig. 8b–d and Supplementary Fig. 17; *n* = 5/*N*; *N* = 3): the PR loss in HT-HROs (40 ± 12%, RCVRN/DAPI area) compared with controls was significantly reduced by GsMTx4 (85 ± 10%, *P* < 0.0001) and almost completely prevented by MEKi (96 ± 11%, *P* < 0.0001). Not only were cone and rod loss averted, but also PR extrusion and PIS loss (Fig. 8b–d and Supplementary Fig. 17b). Both inhibitors also reduced glial reactivity (GFAP/DAPI, *P* < 0.0001), proliferation (SOX9/DAPI, *P* < 0.0001), and cell delamination (Supplementary Fig. 17b, c). Given the condensed actin fibers in MG (Fig. 5c3’,4’), increased RHO-ROCK-MYOSIN-related gene expression (Fig. 7e), and actomyosin function in extrusion^63^, we tested if a widely-used inhibitor for non-muscle myosin II, (−)-Blebbistatin (BLEB), (differentially) affects PR and MG pathologies. To study PIEZO1 function by a different approach, we applied its chemical activator YODA1, either alone or combined with HBEGF or TNF. Strikingly, YODA1 alone was sufficient to induce a severe cone (42% ARR3, *P* < 0.0001) and rod loss (49% NRL, *P* < 0.0001) via cell extrusion (Fig. 9, Supplementary Fig. 18, and Supplementary Data 6). YODA1 also caused gliosis, but MG number increased only when combined with HBEGF or TNF. BLEB partially prevented the cone/rod loss, and also PR ectopy, PIS loss, and MG proliferation (Fig. 9, Supplementary Fig. 18, and Supplementary Data 6). However, BLEB reduced neither gliosis nor delamination, suggesting an actin function downstream of PIEZO1/MAPK. Generally, TUNEL assay indicated a near absence of cell death in situ (Fig. 8b and Supplementary Fig. 18b). Notably, HBEGF or TNF applied alone were not sufficient to induce a complex pathology within the studied timeframe (Fig. 9 and Supplementary Fig. 18) supporting earlier studies^23,31,39,40,42–44^ and indicating synergistic and additive HT functions^50^ previously unknown for the nervous system.

## Discussion

**Fig. 10:**
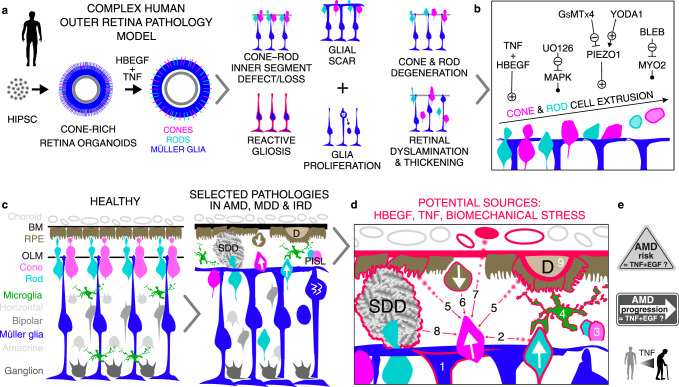
Summary: HT-HRO model phenotype, mechanism, and potential relation to retinal pathologies. **a** Scheme of the HBEGF-TNF (HT)-induced pathology model in human retinal organoids (HT-HRO): several distinct histopathologic processes progressively develop in a spatiotemporally-combined and quantifiable manner. Cone and rod photoreceptor (PR) degeneration via apical cell displacement (ectopy) out of the retina with subsequent cell death, combined with several Müller glia (MG) pathologies, is one complex phenotype. (Ultra)structural, molecular, and functional pharmacological inhibitor data indicate a mechanism: (**b**) HT application or PIEZO1 activation induces PR degeneration via cell extrusion with or without extensive glial pathologies, respectively. Inhibition of MAPK (UO126), PIEZO1 (GsMTx4), or actin-myosin (BLEB, Blebbistatin) signaling each effectively prevents not only PR extrusion but also the complex phenotype. **c** Summary scheme depicting healthy human retina and selected pathologic processes potentially relevant for AMD and other pathologies, and **d**, **e** potential sources and functions of TNF, HBEGF (EGF may have a similar effect), and biomechanical stress in retinal pathologies (indicated in red and numbered) as a basis to discuss and speculate about the potential relevance of the pathologic processes reproduced in the HT-HRO model for patients. Some key hypotheses: PR degeneration by extrusion might underlie PR displacement (white arrow), possibly associated with aging, advanced AMD, and some other pathologies. PIS loss (PISL) is a hallmark of definitive and irreversible vision loss, which might cause or be a consequence of PR extrusion and scarring. Glial scar formation might cause/contribute to PR extrusion, and complex or endstage retinal pathologies. Pathologies might induce HT from various sources (numbered), including retinal cells, microglia, or systemically, and changes in retinal structure or biophysical cell or tissue properties, e.g., due to extracellular pathologic material or changes in choroid/RPE. **e** See Supplementary Data 1. Image legend: (1) Müller glia; (2) PR; (3) PR inner segments (PIS); (4) microglia; (5) RPE, retinal pigment epithelium; (6) extruding RPE; (7) systemic/entire body; (8) SDD, subretinal drusenoid deposits (extracellular pathologic material between retina and RPE), which might result from extruded photoreceptors^13,48,61^ (white arrows); and (9) D, Drusen (extracellular pathologic material below the RPE). BM Bruch’s membrane, OLM outer-limiting membrane.

Here, we show in a human model system that combined (not separate) stimulation with HBEGF-TNF (HT) is sufficient to induce a series of pathologic processes that dynamically and progressively develop as one complex phenotype (Fig. 10a). PR degenerate in combination with glial pathologies: reactive gliosis, proliferation, scarring, dyslamination, and retinal thickening (despite PR loss)—a potential biomarker for complex pathologies^7,10,15,16,30^. This model revealed a mechanism of PR degeneration by cell extrusion mediated by inflammatory and biomechanical regulators (Fig. 10b)—potential therapeutic targets to prevent not just PR extrusion, but even a complex pathology; and this model offers hypotheses for pathologies (Fig. 10c, d).

One promise of organoid technology is the reproduction of complex neuropathologies, and the HT-HRO model advances our general understanding of the pathogenicity and interrelationship of several simultaneously-developing pathologies, and provides a starting point for even more complex models. HROs are a reductionist system advantageous for focusing on major cell types. However, others, like microglia which contribute to pathologies, are missing. It is still unclear whether HT is similarly pathogenic in vivo, or if this requires cell stress potentially present in HROs but not in healthy individuals, and if it depends on the cell composition. We used a cone-rich HRO system robustly reproducing PR and MG cell composition, and several parameters found at the interface of the fovea–parafovea. Conversely, common animal models^6,73^, mouse retinal organoids^73^, and most other HRO systems have fewer cones (cone:rod ratios between 1:2 and 1:5)^33–35,38^, at least one correlates with the peripheral retina^36^, and another one is cone-rich^37^. Since PR degeneration in the fovea–parafovea is prominent in intermediate/advanced AMD and other pathologies^4,7,11^, it will be interesting to determine if HROs could also reproduce other region-specific features. Comparisons to macular development, retinal subregions, and validation of the HT-HRO model in primary human retinas and animals will advance our understanding.

An experimentally induced HRO model at about late fetal-like stage potentially reproducing gene mutation- or aging-related pathologic processes might be far-fetched and a contradiction; however, the HT-HRO model has started to reveal mechanisms that can be used to investigate this. While our data offer several starting points for validation, determining if distinct processes in models might be common across different diseases, or if an entire phenotype might be a reduced model for part of a primary or subsequent stage of a distinct pathology remains an unsolved question. Validation might initially be straightforward for gene mutation-based models, by assessing defined protein expression deficits and their rescue upon gene repair, but pathogenesis might differ between models and patients. For example, timing, phenotype, and mechanisms might depend on the environment and cell composition. Another major question is to what extend AMD can be modeled due to its complex etiology, specifically, aging and lifestyle risk factors. HROs at D150−250 might have properties designating them more vulnerable, which could change upon further maturation and reverse with aging/disease^29^. Via transcriptome analysis, we identified potential regulators of the HT-HRO model, but patient data is still too limited for effective comparative analyses. Thus, we discuss and speculate about the potential relevance of the HT-HRO model for retinal pathologies (Fig. 10c, d) and about validation strategies:

The HT-HRO model revealed PR degeneration by cell extrusion, which might underlie PR displacement in patients^10,12,13,15,18,29,32,51–54,56,59–62^ and animals^16,56^ with various types of pathologies. So far, it is still unclear to what extent PR displacement might cause or contribute to pathogenesis and thus vision loss, and if different mechanisms exist. Differential extrusion mechanisms have been shown in other organs^63,64^. Conceptionally, extrusion involves several regulation levels^63,64^: endogenous or exogenous processes may induce extrusion of dead or living cells, singly or in clusters; execution might require several processes, either cell autonomous, like migration, non-autonomous, like neighboring cells releasing cell contacts, or both. Extrusion might have different functions and consequences. Thus, HT treatment might either induce cone and/or rod extrusion directly, both competing and regulating each other, or indirectly via MG; our data support both possibilities.

HT treatment might cause a complex phenotype by inducing PR and MG pathologies in parallel or interdependently (Fig. 10a). HBEGF, like EGF, binds to and activates EGFR, and ERBB receptors. TNF signals via two receptors, often with opposing functions^50^, like cell death and neuroprotection. Retinal damage-induced EGFR signaling regulates MG proliferation in animals^31,44^. Disease-induced systemic or local TNF (Fig. 10d) might stimulate EGFR via TNF receptors in MG;^23,31,39,40,44^ each pathway may activate the other^50^ to induce MG pathologies, possibly causing PR extrusion. Pathologic changes, like cell crowding or other biomechanical forces, activate PIEZO1-mediated extrusion in monolayered epithelia^63,64^. Thus, neighboring MG or PRs might squeeze extruding PRs out, or PRs migrate out. Since YODA1 mainly causes PR extrusion (possibly stimulating PRs directly), HT treatment might induce PIEZO1-mediated PR extrusion via the other pathologies. Ultrastructural and live-imaging data support the notion of a PIEZO1-mediated biomechanical process: PRs appear to squeeze through the OLM, possibly neighboring cells try to prevent or cause it. Biomechanical changes may be caused by inflammation, or vice versa via PIEZO1^63,64^, and be involved in neuronal and glial pathology. Transcriptomics of the HT-HRO model suggests upregulation of *PIEZO1*, and other genes indicative of glial reactivity and neurotoxicity, as in brain astrocytes upon aging^43^, TNF^43^, inflammation^2^, gliosis^23,31^, microgliosis^43^, and extracellular deposits^43,70^. PR damage might initiate PR extrusion, HT induces pathologic changes of PIS, either caused by HT directly, or indirectly via MG constrictions, an early process of extrusion in other organs^63^. PIS defects and loss, a common clinical biomarker for the onset of irreversible vision loss^10,18,23^, mitochondrial damage^4,5,10,13,18^, and PR displacement also occur in AMD and other pathologies. Ultimately, PRs appear to die upon extrusion while still in contact with the retinal surface. Since TNF impairs epithelial barriers in other organs, it might regulate OLM integrity via CRB1^16,74^ and contribute to different cell displacement phenotypes, like large PR cell clusters upon CRB1 mutations^16,60^. As shown for other cell death mechanisms^40^, some pathologies might involve PR degeneration by displacement in parallel to in situ cell death mechanisms. Systematic studies of PR extrusion and death, and MG pathologies in patients and models might provide further insight. Taken together, our data fuel the hypothesis that inflammation, biomechanical, oxidative, and metabolic stresses—all potential cell-extrusion inducers^63^, might induce pathologic changes in PRs, RPE, MG, and other cells upon aging and retinal diseases, that might be interrelated, and form a vicious cycle contributing to pathogenesis.

Notably, not only PRs, but also the opposing RPE become ectopically displaced in AMD^24,75^ and possibly in other pathologies (Fig. 10d); this raises the question if both pathomechanisms are related (e.g., HT-inducible). Ectopic RPE is an established biomarker and risk factor for AMD progression^75^, but ectopic PRs not yet. The degree of both RPE and PR loss have been suggested to associate with AMD progression^8,14^; and some studies predicted an association of cones and MG with AMD risk^10,20,23,68,76^. Extruded PRs might be a cause of, and/or contributor to, the development and progression of retinal degeneration: initially, PR extrusion might even have a cell protective function, with HBEGF suppressing the apoptotic TNF functions^42^, potentially facilitating PR live-cell extrusion. However, at some point ectopic PRs might undergo cell death, which might attract immune cells for their removal^2,61^ and contribute to extracellular deposits^12,13,61^, inflammation, remodeling, and thus progression of retinal degeneration.

The HT-HRO model potentially reproduces parts of retinal remodeling (Fig. 5a), which might be relevant for advanced AMD^10,19–22,24,26–29^, complex IRDs/MDDs^15–17^, and (secondary) degeneration in most other pathologies at late stages^5,41^. Notably, the mechanisms and functions of glial scars are still unclear, and a contribution to advanced AMD has been suggested^7,10–13,18–28^. Thus, it is tempting to speculate that the temporal stages of the HT-HRO model might reproduce some aspects of the spatial stages in the progressing retinal lesions in AMD—an opposing wedge-shaped transition zone containing all stages of pathogenesis: one wedge ranges from the healthy retina, followed by extruded and degenerating PRs in an otherwise seemingly intact retina, and a subsequently increasing region with PR depletion^8–12,14,18,19,24,26^. This PR loss is superseded, interspersed, and concluded by an inverted wedge of scar formation^7,10,12,18–22,24,26,28^. At the tip of this wedge, the leading edge of PR degeneration, displaced MG form a scar tip that might push the lesion forward or follow its tail^19,24^. The HT-HRO model might assist the development of imaging-based phenotypic^24^ and other biomarkers for PR extrusion and MG pathologies, facilitating disease diagnosis, stratification, and monitoring.

A fundamental question promoted by our work is whether experimental HT challenge might mimic parts of a (chronic) pathologic HT exposure, potentially contributing to various neuropathologies in patients/animals^39–44^, particularly the predicted potential function of TNF-/EGF-signaling components as AMD risk or progression factors^1,45–48^ (Fig. 10e and Supplementary Data 1). HT might also increase secondarily due to AMD risk factors such as aging or complement activation, or due to other pathologic processes, like inflammation or biomechanical stress, in various diseases, but their relationship to retinal disease processes, particularly age-dependent PR displacement^29,51–54^, is still unclear. HT-induced pathologic changes might also variably occur in addition to primary disease processes and thus contribute to phenotype variability. TNF is a well-known inducer of the complement system, and vice versa, as well as of inflammation, as observed at advanced disease stages^3,41^. Aging and extracellular deposits might also affect retinal biomechanics, which could stimulate inflammation, HT expression, PIEZO1 activation, and thus neuropathology. Larger-scale studies could determine if systemic or intraocular HT levels increase prior to or after pathogenesis, and if so, whether they associate with any pathologies reproduced in the HT-HRO model.

In conclusion, we established an inducible, effective, dynamic, and complex human retinal pathology model, which offers access to unsolved processes like PR displacement and scarring, potentially relevant for various pathologies. Our data suggest mechanisms and biomarkers that might advance research on disease risk, subtypes, pathogenesis, and therapies in the nervous system: HT and biomechanical stress might induce or exacerbate neuropathologies. The HT-HRO model might provide a preclinical human system to facilitate the development and translation of biomarkers, and gene-, cell-, and drug-based therapies.

## Methods

### hiPSC generation and maintenance

All experiments involving hiPSCs were performed in accordance with the ethical standards of the institutional and/or national research committee, as well as with the 1964 Helsinki Declaration and its later amendments, and approved by the ethical committee at the Technische Universität Dresden. In this study, one newly generated hiPSC line CRTD1 (https://hpscreg.eu/cell-line/CRTDi004-A), and three previously published ones 5A^77^, IMR90^78^ (iPS(IMR90)−4, WiCell; https://hpscreg.eu/cell-line/WISCi004-B) and CRTD2 (https://hpscreg.eu/cell-line/CRTDi003-A) were used: these were derived from cells of healthy human donors (Supplementary Fig. 1). The CRTD1 hiPSC line was generated from previously published foreskin fibroblasts (termed Theo) of a consenting healthy donor^79^, and the CRTD2 hiPSC line was generated from CD34-positive cells isolated from peripheral blood of a consenting healthy donor. Isolation of cells and reprogramming to hiPSCs was approved by the ethics council of TU Dresden (EK169052010 und EK386102017 for CRTD1 hiPSC, EK 363112012 for CRTD2 hiPSC). Reprogramming of the CRTD2 hiPSCs from CD34-positive cells has been described previously^80^. For CRTD1 hiPSCs, fibroblasts were reprogrammed at the CMCB Stem Cell Engineering Facility at Technische Universität Dresden using the CytoTune-iPS 2.0 Sendai Reprogramming Kit (Thermo Fischer Scientific) according to the supplier’s recommendations for transduction. Following transduction with the Sendai virus, cells were cultured on irradiated CF1 mouse embryonic fibroblasts (Thermo Fisher Scientific) in KOSR-based medium (80% DMEM/F12, 20% knockout serum replacement, 2 mM l-glutamine, 1% nonessential amino acids solution, 0.1 mM 2-mercaptoethanol, all from Thermo Fisher Scientific) supplemented with 10 ng/ml human FGF2 (Stem Cell Technologies). Individual hiPSC colonies were mechanically picked, expanded as clonal lines, and adapted to Matrigel/mTeSR1/ReLeSR conditions after several passages.

Master and working hiPSC banks were established for the study. All hiPSC lines used in this study were maintained on Matrigel-coated culture dishes in mTeSR1 (Stem Cell Technologies) and passaged using ReLeSR (Stem Cell Technologies). To characterize the CRTD1 and CRTD2 hiPSC lines, the following tests were performed:

For flow cytometric analysis of pluripotency, Alexa Flour 488 anti-OCT3/4, PE anti-SOX2, V450-SSEA-4, and Alexa Flour 647 anti-Tra-1-60 were used. All antibodies were obtained from BD Pharmingen (Supplementary Data 7) and used according to the manufacturer’s recommendations.

Three-germ layer differentiation was performed as described previously^81^. Three-germ layer immunocytochemistry was performed using the 3-germ Layer Immunocytochemistry Kit (Thermo Fisher Scientific A25538) according to the instruction manual. For the endoderm, SOX17 antibody was used (see Supplementary Data 7).

QRT-PCR for pluripotency and trilineage spontaneous differentiation was performed according to the instruction manual of the human ES cell Primer Array (Takara Clontech).

Standard G banding karyotyping was performed by the Institute of Human Genetics, University Clinic Jena, Germany.

### Human retinal organoidogenesis

Human retinal organoids (HROs) were differentiated using a protocol modified from previous works^82^. Briefly, undifferentiated hiPSCs were passaged to small cell clumps using ReLeSR (Stem Cell Technologies) and suspended in Matrigel (growth-factor reduced, BD Bioscience) for gelification. The almost solid gel was gently dispersed into small clumps in a floating culture in six-well low-attachment plates (Nunclon Sphera, Thermo Fisher) in N2B27 medium (1:1 DMEM/F12: neurobasal A medium, 1% B27 + Vitamin A, 0.5% N2, 1% penicillin/streptomycin, 1% GlutaMAX, 0.1 mM 2-mercaptoethanol). Cell clumps spontaneously generated epithelial cysts with one single central lumen within 1−2 days (D). On D5, cysts were plated onto Matrigel-coated six-well plates, and media was changed every second day. On D14, adherent cultures were detached as intact cell sheets using Dispase (Stem Cell Technologies) and transferred to a floating culture in B27 medium (DMEM/F12, 1% B27 without vitamin A, 1% penicillin/streptomycin, 1% GlutaMAX, 1% NEAA, 0.1% amphotericin B) in 9 cm low-attachment plates (Nunclon Sphera, Thermo Fisher). Retinal epithelial domains were manually isolated between D25 and D30 using surgical tweezers (Fine Science Tools, Dumont No. 5) under a microscope. 10% FBS was added to the B27 medium from D25. On D100, the medium was changed to N2 + FBS + DMEM/F12 (DMEM/F12, 1% N2, 10% FBS, 1% penicillin/streptomycin, 1% GlutaMAX, 0.1% amphotericin B). The medium was supplemented with synthetic retinoid analog EC23 (0.3 μM) from D25 to D120. From D14 onwards, 50% of the media was replaced every 2−3 days.

### Experimental design of HBEGF-TNF-induced human retinal pathology model

HROs were cultured under the standard conditions described above. For each experiment, HROs were derived from the same original culture plate and divided into treatment and control culture plates when the experiment was started at the indicated timepoints. For the HT challenge (treatment plate), recombinant human HBEGF and recombinant human TNF (HT) (both R&D) were both applied to the culture medium daily, each at a final concentration of 50 ng/ml. Controls received only sterile water (cell-culture grade, 1 µl/ml daily) and were run in each experiment and for each timepoint. In total, 50% of the media was replaced every second day. To study HT phenotype at three different HRO ages (i.e., timepoints after completion of HRO generation), HT and control conditions were applied to separated groups of HROs from the same differentiation batches, at D150, D200, and D250. This was repeated in independent experiments (HROs from independent rounds of differentiation batches): *N* ≥ 3 independent experiments with *n* ≥ 5 organoids per experiment were analyzed per timepoint (*N* = 3 for day 150, all others *N* = 4). Samples were taken after 10 days of HT treatment, i.e., at D160, D210, and D260, respectively, and processed for histological analysis. To determine the temporal dynamics of the HT phenotype development (i.e., days of HT challenge), HT and control conditions were applied to separated groups of HROs from the same differentiation batch, starting at D200, and samples were taken at indicated timepoints (control samples were collected at D202, D210, D220, and D240), and processed for histological analysis (*n* ≥ 5 organoids per condition in *N* = 1 independent experiment analyzed). To assess HT concentration dependency, we studied 2.5, 5, 20, and 50 ng/ml daily each from D200 to D220 (*n* ≥ 5 organoids per condition in *N* = 2 independent experiments (2.5 ng/ml *N* = 1)). Pharmacological inhibitor and agonist experiments were performed as follows: HROs were differentiated until D200 and then separated into the different treatment groups. HT was applied daily at a final concentration of 50 ng/ml from D200 to D210. Pharmacologicals were applied starting 12 h before the start of the HT treatment and then throughout the whole HT treatment period. In the first set of experiments the following inhibitors were used: MEK inhibitor U0126 (Calbiochem, Merck) was applied daily at a final concentration of 10 µM (stock solution 20 mM in DMSO); further PIEZO1 and mechanosensitive ion-channel blocker GsMTx4 (Abcam) was applied at a concentration of 5 µM and 50% of the compound was replaced at media change every second day (stock solution 200 µM in cell-culture-grade water). In a second experiment set, (−)-Blebbistatin (Cayman Chemicals) was tested at 20 µM, 50% replaced at media change (stock solution 15 mM in DMSO). Further, PIEZO1-channel activator YODA1 (Tocris) was applied to HROs without HT treatment from D200 to D210 at 5 µM (stock solution 10 mM in DMSO, applied daily). YODA1 also was tested in combination with TNF (50 ng/ml daily) or HBEGF (50 ng/ml daily). TNF (50 ng/ml daily) and HBEGF (50 ng/ml daily) were also applied separately from D200 to D210 in these experiments. Controls and HT-treated HROs received equal amounts of solvents (DMSO and water). Samples were taken on D210 and processed for immunohistochemistry. *N* = 3 independent experiments with *n* ≥ 5 organoids per *N* were analyzed per treatment condition.

### Live-imaging of photoreceptor cell extrusion

Whole HROs were imaged with a Zeiss spinning disk confocal microscopy system based on an inverted Axio.Observer Z1 stand with a Yokogawa CSU-X1 and a cage incubator (Supplementary Data 3; Plan Apo 20×/0.8 objective; imaging frequency four frames per hour; total stack sizes 130−200 µm; slice distance 2 µm). For en-face imaging (Fig. 3b) of whole living HROs, the optical plane was positioned at the PIS level and imaged from above at an orthogonal angle (en-face). To capture cell extrusion out of the apical retinal border and ectopic cells positioned on the outside organoid surface, we performed live-imaging by taking optical image stacks acquired with a retinal cross-sectional focus plane (Fig. 3c and Supplementary Movies 2–4). Samples were incubated during live-imaging (phenol red-free N2 + FBS + DMEM/F12 medium, µ-dishes (ibidi), 37 °C, 5% CO_2_). The nuclear live-dye SiR-DNA (150 nM, tebu-bio) was added two hours before imaging. At the end of time-lapse imaging, cell ectopy was verified by adding sodium-fluorescein (Fluorescein Alcon 10%; 1:10,000) to the cell-culture media to differentiate the apical organoid boundary from the surrounding space (Supplementary Movie 4).

### Histological analysis, imaging, and quantification methods

#### Immunohistochemistry

For immunohistochemistry of HRO wholemounts or cryosections, HROs were fixed in 4% PFA in PBS. For cryosections, HROs were cryoprotected in a graded series of sucrose solutions, and embedded in O.C.T. compound (Sakura Finetek). Organoids were serially sectioned at 12-μm thickness using a Leica CM3050 S cryostat, mounted on Superfrost Ultra Plus slides (Thermo Scientific), and stored at −80 °C. Sections were washed in PBS for 15 min and, if necessary, antigen retrieval was performed via citrate (10 mM sodium citrate, pH 6.0, 30 min at 70 °C). The tissue was treated for 30 min at RT in blocking solution (0.5% BSA and 0.3% Triton-X-100 in PBS), then incubated with the primary antibodies (48 h, 4 °C). The tissue was washed in PBS (3 × 10 min), and species-specific secondary antibodies conjugated to fluorophores (488, Cy3, 649; Dianova, 1:1000) were applied for 1 h at RT. Nuclei were stained using DAPI (AppliChem). The tissue was washed again in PBS and coverslipped using Fluoromount-G (Southern Biotechnology). A TUNEL assay for cell death analysis was performed before primary antibody incubation using the In Situ Cell Death Detection Kit Fluorescein (Sigma-Aldrich, Roche products) according to the manufacturer’s instructions. Filamentous actin was visualized using Phalloidin488 (PD, Invitrogen) staining (1:500, 15 min at RT) after secondary antibody incubation. The primary antibodies used in this study are listed in Supplementary Data 7.

#### Imaging and quantitative analysis of HRO cellular changes

Samples were imaged on a Zeiss ApoTome2 (quantitative analysis) or Zeiss spinning disk confocal microscope (inner segment markers). Random regions of interest (ROIs) of 100-μm width along the outer (apical) surface of the ROI on central HRO sections were used for cell counts and co-localization analysis. The *x* axis of each ROI was positioned radially to the organoid center, with the *y* axis aligned perpendicular to the organoid surface. Height of the ROI was set to include the entire epithelial width. ROI images are *z* axis projections of 5 × 1 μm, i.e., 5 planes, 1 μm apart acquired in Apotome mode using a ×20 Plan-Apochromate objective. For cell counts, images were 3D reconstructed (maximum-intensity projection) and counted manually using Fiji. Two ROIs per organoid, positioned on opposing sides of the organoid, were imaged and analyzed; the mean of the two ROIs per individual organoid (*n*) was used for statistical analysis, except in Supplementary Fig. 2b (six ROIs per organoid). For quantitative analysis of RCVRN and RLBP1 staining, images were automatically thresholded in Fiji (mean threshold mode) and the pixel area above the threshold was measured and normalized to DAPI (pixel area data).

Photoreceptor inner segments (PIS) and extruded photoreceptor cells were quantified on 450 × 335 μm ROIs (acquired as described above for cell counts) and normalized to the exact organoid apical surface circumference measured within each ROI. Two ROIs were analyzed per HRO. PIS were defined as individual RCVRN + photoreceptor protrusions outside of the apical retinal epithelium border that did not co-localize with cell nuclei (DAPI-negative). Extruded photoreceptor cells were defined as marker-positive cells (RCVRN, NRL, or ARR3) with the nucleus (DAPI) positioned outside (apical to) the apical retinal epithelial border, which was determined based on cell nuclei (DAPI) and MG markers (RLBP1 and SLC1A3). Cell nuclei labeled with phosphohistone-3 (PHH3) were counted per entire organoid section, and normalized to each organoid circumference. Organoid circumference, epithelial thickness, and section area were measured on microscopic images of immunostained entire central organoid sections using Fiji; for all parameters, the apical organoid boundary was defined using DAPI staining, and the basal boundary by RLBP1 + SLC1A3 and DAPI staining.

#### Quantitative analysis of retinal dyslamination

Structural retina dyslamination was assessed by cell-delamination analyses for photoreceptors, MG, and total cell numbers in HROs (RCVRN+ cells, SOX9+ or DAPI+ cell nuclei, respectively) performed on microscopic ROI images (see criteria above). Fiji software macros^83^ were used to automatically threshold images and detect the precise position (*x/y* coordinates) of positive signals. Local maxima detection (3D maxima finder, 3D ImageJ Suite) was used to determine the position of individual SOX9+ cell nuclei. The position of each pixel above the threshold was determined for RCVRN and DAPI signals. Each ROI was divided into ten equally sized sections from the apical to the basal organoid surface (*y* axis), and the relative number of local maxima (SOX9+ cell nuclei) or pixels (RCVRN or DAPI) detected was calculated for each section (see Supplementary Fig. 8d). For analysis of amacrine (PAX6 + SOX9 − cells) and bipolar (VSX2 + SOX9 − cells) neuron delamination, *x* and *y* coordinates were determined by manual selection in Fiji (*n* = 5 HROs from *N* = 2 experiments with 4 ROIs per HRO analyzed). Further analysis and visualization of the cell distribution was done as described for the SOX9 cell delamination analysis.

#### Intraorganoid photoreceptor and MG distribution

To analyze rod and cone, as well as MG cell distribution within an HRO, cryosections were stained for NRL/ARR3/DAPI or SOX9/DAPI, respectively. ROIs were acquired as described above, but six ROIs per organoid were recorded at different random positions within a central HRO section to reflect cell distribution across the organoid. Images were 3D reconstructed (maximum-intensity projection) and counted manually using Fiji. Five HROs per experiment were analyzed (six ROIs each) for a total of four independent experiments (*N*) derived from two hiPSC lines (5A and CRTD1, *N* = 2 each).

#### Analysis of the photoreceptor pattern

Whole HROs (PFA fixed) were stained for the glia markers SCL1A3 and RLBP1, and en-face organoid images were acquired with a Plan Apo ×40/0.95 objective on a spinning disk confocal microscope (Zeiss). The ROI was set to the area where apical glial processes forming the OLM could be imaged at an orthogonal angle (en-face). Due to the HRO shape, the ROI size was limited to 40 × 40 µm. Images were thresholded manually using Fiji software. For pattern analysis, a custom-made Fiji macro^84^ was used based on GPU-accelerated image processing (CLIJ)^85,86^ and machine-learning tools for pixel and label classification (deposited on Zenodo repository; see “Data availability”). In brief, the software was first trained to distinguish between signal and background (CLIJx weka pixel classifier) and second to classify the cells into a larger and a smaller subpopulation (type 1 and type 2, probably cones and rods, respectively) (CLIJx weka label classifier). In the following steps, cell numbers, cell sizes, nearest neighbor distances, and the number of voronoi neighbors were analyzed for every single cell. 8 HROs from one experiment were analyzed with three ROIs per HRO (except for 1 HRO with only 1 ROI). All samples were checked for proper cell detection and segmentation. In total, 1800 photoreceptor cells were analyzed (type 1: 932 cells, type 2: 868 cells). Edge cells that crossed the image border were excluded from the cell diameter, nearest neighbor distance, and the number of neighbors analyses (*n* = 1360 photoreceptor cells without edge cells: type 1: 727 cells, type 2: 633 cells).

#### Cone photoreceptor cell density analysis

Whole HROs (PFA fixed) were immunostained for ARR3, OPN1LW, and DAPI. HRO cap regions were imaged using a spinning disk confocal microscope (Zeiss) with a Plan Apo ×40/0.95 objective. The optical stack size was 5 µm, starting at the very first DAPI+ nuclear layer that became visible when the sample was approached from the apical surface. Seven HROs out of one experiment (N) with one ROI each were analyzed. ROIs were cropped to a size of 120 × 120 µm to remove the image borders that showed the inner segment level of neighboring photoreceptors but not their nuclei (due to the spherical shape of the HROs). Positive cells were counted from maximum-intensity projections (Fiji) and defined as nuclei clearly surrounded by a cytoplasmic fluorescent signal.

#### Analysis of Müller glia seal-like scar formation

Whole HROs (PFA fixed) were stained in two separate channels for RCVRN and combined SLC1A3 and RLBP1. Whole HROs were imaged with a Plan Apo ×20/0.8 objective using a spinning disk confocal microscope (Zeiss). The region of interest (ROI) was set to the area where POS/PIS could be imaged en-face (angle of view orthogonal to the outer-limiting membrane). Z-stacks of optical image sections with 6 µm thickness and at 0.3-µm slice distance were acquired. These image stacks ranged from the RCVRN + POS/PIS (as the apical ROI limit) to the region of the OLM (as the basolateral ROI limit) defined by the beginning of SLC1A3 + RLBP1 + MG as a proxy. ROI size was dependent on the HRO shape and was at least 69 × 69 µm. Maximum-intensity projections were created and converted to a binary image using automatic thresholding in Fiji software. The area fractions of RCVRN + and SLC1A3 + RLBP1 + pixels were determined. Nine HROs (*n*) from one experiment were quantified per group (mean of three ROIs/*n*).

### Flowcytometry analysis of HRO cells

#### Immunostaining for retinal cell-type analysis of dissociated HRO cells

HRO samples were pooled (6−9 HROs per sample, at D210−220) for each experiment and variable (control and HT-treated, 10 ± 1 days of treatment), and dissociated using the Papain Dissociation System (Worthington Industries) according to the manufacturer’s instructions. Briefly, pooled samples were incubated in 500 μl of papain (20 U/ml, 2 h, 37 °C) in an orbital shaker incubator (ES 20/60, Biosan, 100 rpm), followed by manual trituration through a fire-polished glass pipette. Dissociated cells were resuspended in PBS with 0.04% BSA and subsequently fixed with PFA (1% in PBS, 15 min, RT) on an orbital shaker (0.4 g), washed once with PBS, and centrifuged (10 min, 480 × *g*). Cell suspension was treated with a blocking agent (10% FBS, 0.1% Triton in PBS) for 10 min on the orbital shaker. Subsequently, the cell suspension was divided into separate tubes, for the different sets of antibodies detecting RCVRN-FITC and ARR3, and SOX9 and ARR3 (see Supplementary Data 7). ARR3 and SOX9 primary antibodies were detected by fluorescently conjugated secondary antibodies (1:1000; AlexaFluor 647 anti-mouse and Alexa Flour 488 anti-rabbit, Dianova). Cell suspensions were protected from light and incubated with antibodies for 30 min on the orbital shaker (200 rpm); as indicated, secondary antibodies were added halfway through incubation time. After washing with PBS, cells were centrifuged (6 min, 600 g), resuspended in PBS with 0.04% BSA, and filtered through a Flowmi cell strainer (40 μm, BelArt, SP Scienceware). *N* = 3 independent experiments from 2 hiPSC lines: 5 A (*N* = 2) and CRTD1 (*N* = 1). Within each experiment, 2−3 technical replicate samples (each 6−9 HROs pooled) for both variables (CTRL and HT-treated) were collected and independently processed (dissociated, stained, and analyzed using flow cytometry (see below)). To validate antibodies, we performed immunostaining (Supplementary Fig. 6b) and imaging flow cytometry (method see below, Fig. 2e and Supplementary Fig. 6c). In all, 30−40 × 10^3^ cells per sample were analyzed.

#### Flow-cytometry-based live/dead discrimination of dissociated cells

Cells were dissociated using papain as described above, resuspended in PBS with 0.04% BSA, and filtered through a Flowmi cell strainer (40 μm). Live-dead staining was performed using the LIVE/DEAD™ Viability/Cytotoxicity Kit (Thermo Fisher) according to the manufacturer’s instructions. Briefly, cells were incubated with a final concentration of 8 μM ethidium homodimer-1 (2 mM in DMSO/H_2_O 1:4) and 0.1 μM Calcein AM (50 μM in DMSO) for 15 min at RT and then analyzed by flow cytometry. 30−40 × 10^3^ cells per sample were analyzed (4 samples, 2 HROs/sample, *N* = 1 independent experiment, 1 hiPSC line (CRTD1)).

#### Flow-cytometry analysis of dissociated HRO cells

The immunostained or live-dead kit-labeled dissociated HRO cells were analyzed using flow cytometry on a BD FACS Aria III cell sorter (100-μm nozzle, 20 psi sheath pressure). For cell-type analysis only, the nuclear dye DAPI was applied shortly before analysis, to enable distinction between nucleated cells and membranous debris. Fluorescence of DAPI, GFP/Cy2/A488, and Cy5/A647 was measured at 405 nm (bandpass filter (BP) 450/40 nm), 488 nm (BP 530/30 nm), and 633 nm (BP 660/20 nm), respectively. For live-dead discrimination, the fluorescence of Calcein (viable cells) and ethidium homodimer-1 (dead cells) was measured at 488 nm excitation using BP 530/30 nm and BP 610/20 nm filters, respectively. Data analysis was performed with FlowJo software (FlowJo, LLC), v.10.5.3. As a proxy for MG hypertrophy (defined as cell size), the median forward-scattered light area (FSC-A) of the SOX9+ cell population was determined, which is proportional to cell surface area or size^87^. For cell-type and MG-hypertrophy analysis, 1237−21024 (median: 7279) SOX9+ cells per sample were included. Each sample consisted of 6−9 pooled HROs. In all, 2−3 technical replicates (samples) per independent experiment (*N*) and variable. *N* = 3 independent experiments from two hiPSC lines: 5 A (*N* = 2) and CRTD1 (*N* = 1). Further, to determine if any cells are co-labeled for MG and photoreceptor markers, single cells were analyzed for double positivity of SOX9 and ARR3 using the BD FACSDiva software: 32,000−40,000 cells within all events were used for gating, and SOX9 + ARR3 + events ranged between 8 and 255. To validate that the antibodies differentially immunolabeled HRO-derived cells, we also performed imaging flow cytometry using an Amnis ImageStream X Mk II imaging flow cytometer (Luminex) after gating cells for nucleated singlets. For sample excitation, the following laser intensities were used in separate channels: DAPI: 405 nm laser, 20 mW; FITC/Alexa488: 488 nm, 20 mW; Alexa647: 642 nm, 150 mW; SSC: 78 5 nm, 4.69 mW. The bright-field image was recorded in two channels with LED intensities of 30.84 and 32.74 mW. Image analysis was performed with IDEAS Application v.6.2.64.0.

### Electron microscopy

#### Transmission electron microscopy (TEM)

Organoids were fixed in 4% formaldehyde (FA, prepared from paraformaldehyde pills) in 0.1 M phosphate buffer (PB, pH 7.4) and dissected for TEM, CLEM, and immunohistochemistry using a small razor blade. For TEM, dissected samples were postfixed in modified Karnovsky’s fixative (2% glutaraldehyde, 2% FA in 50 mM HEPES) overnight at 4 °C. Samples were washed twice in 100 mM HEPES and twice in water and postfixed in 2% aqueous OsO_4_ solution containing 1.5% potassium ferrocyanide and 2 mM CaCl_2_ for 30 min on ice. After washes in water, the samples were incubated in 1% thiocarbohydrazide in water (20 min at RT), followed by washes in water and a second osmium-contrasting step in 2% OsO_4_ solution (30 min, on ice). Samples were washed in water, contrasted with 1% uranyl acetate solution for 2 h on ice, washed again in water, dehydrated in a graded series of ethanol solutions, and infiltrated in epoxy resin (EMbed 812). After embedding, samples were cured at 65 °C overnight. Ultrathin sections were prepared with a Leica UC6 ultramicrotome (Leica Microsystems), collected on formvar-coated slot grids, and stained with lead citrate and uranyl acetate. Contrasted ultrathin sections were analyzed on a FEI Morgagni D268 (camera: MegaView III, Olympus) or a JEOL JEM-1400Plus (camera: Ruby, JEOL) both at 80 kV acceleration voltage.

#### Correlative-light electron microscopy (CLEM) of ultrathin cryosections

Small pieces of dissected organoids (less than 0.5 mm) fixed in 4% FA in 0.1 M phosphate buffer (PB, pH 7.4) were processed for Tokuyasu cryo-sectioning^88,89^. In brief, samples were washed in PB, infiltrated stepwise into 10% gelatin (1% for 30 min, 3% for 45 min, 7% for 1 h, 10% for 2 h) at 37 °C, cooled down on ice, incubated in 2.3 M sucrose/water for 24 h at 4 °C, mounted on pins (Leica), and plunge-frozen in liquid nitrogen. 70−100 nm sections were cut on a Leica UC6 + FC6 cryo-ultramicrotome and picked up in methylcellulose/sucrose (1 part 2% methylcellulose (MC, Sigma M-6385, 25 centipoises) and 1 part 2.3 M sucrose).

To facilitate the identification of different cell types in the organoids during CLEM^90^, sections were stained with antibodies for markers of photoreceptors (RCVRN, ARR3, RHO) or Müller glia (SLC1A3), or for actin (ACTB). See Supplementary Data 7 for details on primary antibodies used. Briefly, grids were placed upside down on drops of PBS in an incubator at 37 °C for 20 min, washed with 0.1% glycerin/PBS, blocked with 1% BSA/PBS, and incubated with the primary antibodies for 1 h. When using rabbit primary antibodies, the sections were washed in PBS and incubated directly with protein A conjugated to 10 nm gold particles for 1 h, washed again in PBS, and postfixed in 1% glutaraldehyde (GA, 5 min). When using mouse primary antibodies, sections were incubated with rabbit-anti-mouse bridging antibodies^88^, before being incubated with protein A gold, and postfixed as described above. After that, organoid sections were incubated with fluorescently-labeled secondary antibodies (goat-anti-rabbit or goat-anti-mouse Alexa488, goat-anti-mouse Alexa555) to identify the different cell types in the fluorescence microscope, washed with PBS, stained with 1 μg/ml DAPI for 10 min, and washed in water (10 × 1 min). Grids were mounted in 50% glycerin/water between two coverslips, and imaged using a Keyence Biozero 8000 fluorescence microscope. Sections were demounted, washed with distilled water (10 × 1 min), stained with neutral uranyl oxalate (2% uranyl acetate (UA) in 0.15 M oxalic acid, pH 7.0) for 5 min, washed in water and incubated in methylcellulose (MC) containing 0.4% UA for 5 min. Grids were looped out, the MC/UA film was reduced to an even, thin film and air dried. Finally, the sections were analyzed on a JEOL JEM-1400Plus TEM (JEOL) at 80 kV and images were taken from the exact same sections and regions as for the fluorescence microscopy with a Ruby digital camera (JEOL). Overlays of fluorescent and TEM images were prepared using Fiji and Adobe Photoshop by overlaying structures that are clearly distinguishable both in fluorescence and TEM images, i.e., cell nuclei and junctions.

#### Scanning electron microscopy (SEM)

For SEM, organoids were fixed with 4% FA/PB followed by modified Karnovsky’s fixative (2% GA, 2% FA in 50 mM HEPES). After several washes in HEPES and PBS, samples were postfixed in 1% OsO_4_/PBS for 2 h on ice, washed in PBS and water, and dehydrated in a graded series of ethanol solutions (30%, 50%, 70%, 90%, 96%, 2 × 100% ethanol (on a molecular sieve)). Samples were critical-point dried using the Leica CPD 300 (Leica Microsystems). Dried whole organoids were mounted on 12-mm aluminum stubs, and some were manually dissected using a scalpel. This way, samples preferentially break apart between cell borders. Samples were sputter-coated with gold using a Baltec SCD 050 (Leica Microsystems) and analyzed with a JSM 7500 F cold-field emission SEM (JEOL) at 8 mm working distance and 5−10 kV acceleration voltage using the lower secondary electron detector.

### Bulk RNA-seq and data analysis

#### Library preparation and transcriptome sequencing

For transcriptome analysis, HROs were treated for 10 days with HT (50 ng/ml each daily) or without from D150, 200, and 250, as described above (*n* = 6/*N*, *N* = 1). Individual organoids were lysed in 300 µl TRIzol by mortar-pestle homogenization, RNA was isolated using miRNeasy Kit (Qiagen) and Maxtract High-Density tubes (Qiagen), residual DNA was removed using Ambion TURBO DNA-free Kit (Thermo Fisher), and RNA cleaned up by ethanol-precipitation. RNA concentration was determined using a Qubit 2.0 instrument using the Quant-iT RNA kit (Thermo Fisher Scientific). The RNA integrity for each sample was controlled with the RNA 6000 Nano Assay and the Agilent 2100 Bioanalyzer (Agilent Technologies). All samples included in the experiment had RNA integrity number >7; 100 ng total RNA was used for rRNA depletion. Ribosomal RNAs were removed from total RNA using the Ribo-Zero Gold H/M/R Magnetic Kit (Illumina). A strand-specific library for transcriptome sequencing was prepared using the ScripSeqv2 Kit (Illumina), which was checked using the Agilent 2100 Bioanalyzer system with a High Sensitivity DNA Kit (Agilent). Library concentration was determined using a Qubit 2.0 instrument using the Quant-iT dsDNA High Sensitivity kit (Thermo Fisher Scientific). In total, 40 ng from each library was pooled. Library pool was size selected in a range of 200−600 bp using preparative agarose gel in combination with MinElute Gel Extraction Kit (Qiagen). Single-end sequencing with 30 million reads per sample was performed at a length of 75 bases on HiSeq2500 (Illumina).

#### Analysis of RNA-Seq data

Data processing prior to analysis was performed in uap (v.1.0-alpha)^91^. Sequencing reads were demultiplexed using bcl2fastq (Illumina, v.2.20) and sequencing adapters trimmed using cutadapt (v.1.5)^92^. Quality control used the fastqc program (v.0.11.2) and fastx-toolkit (v.0.013). Reads were aligned to the human genome (UCSC Human Dec. 2013 GRCh38/hg38 Assembly) using hisat2 (v.2.1.0)^37^ and subsequently sorted by name and genomic location using samtools (v.1.1)^93^. New transcripts were generated using stringtie (v.1.3.3, reference annotation: Gencode v.26)^94^, and sample-wise assemblies were merged using stringtie-merge. Subsequently, new transcripts were annotated with respect to their relative genomic position to known genes using cuffcompare (v.2.2.1)^95^. We defined novel transcripts as those classified as u (unknown intergenic), x (antisense exonic), or s (antisense intronic), and kept these and annotated transcripts for further analysis. The number of reads overlapping a known gene from the reference annotation or a new transcript was counted using htseq-count (0.6.1)^96^. Differential gene expression was analyzed using the Bioconductors DESeq2 R package (v.1.22.1)^97^. The variance-mean dependence was estimated in our count data, and the treated (HT) samples were tested against the control samples for differential expression at each timepoint using the negative binomial distribution. Genes with a base mean below 10 were discarded, and for all remaining genes the *P* value was adjusted using the Benjamini & Hochberg adjustment method^98^. All genes with an adjusted *P* value below 0.01 (=FDR) were regarded as differentially expressed. The *Z-*score was calculated by subtracting the population mean from a given data point (e.g., rlog-transformed read counts) and divided by the standard deviation.

Gene-set enrichment was analyzed on the DEG using the Bioconductors Ensemble of Gene Set Enrichment Analysis (EGSEA) R package^99^. We included all available human gene sets (h, c1, c2, c3, c4, c5, c6, c7) from MSigDB^100^, gene sets from GeneSetDB^101^, and KEGG^102^ pathways. The top 20 gene sets were kept for all timepoint comparisons, and clustered according to the expression profile of the underlying genes.

#### Integration of genes-of-interest lists

Genes-of-interest (GOI) lists (Supplementary Data 4) were custom-made based on data in the literature, and integrated with the results of the differential expression analysis by mapping the gene identifiers (ensemble gene ID). To retrieve a reduced and comparable visualization of groups of genes inside the GOI list, the first principal component in terms of an Eigengene of their rlog-transformed expression profiles was calculated.

### Single-cell RNA-seq and data analysis

One individual whole HRO (*N* = 1 experiment, 5A hiPSC line) at D200 of differentiation was dissociated using the Papain Dissociation System (Worthington Industries) as described above. Dissociated cells were resuspended in PBS with 0.04% BSA, passed through a 50-μm filter (CellTrics, Sysmex) to remove cell clumps, and immediately processed for sequencing. Cells were counted in a Neubauer chamber. An estimated number of 9000−11,000 cells were carefully mixed with reverse transcription reaction mix using the Chromium Single Cell 3’ Library & Gel beads chemistry v2 and loaded into a Chromium Single Cell A Chip (10X Genomics, Pleasanton, CA). During the encapsulation process in the 10X Genomics Chromium system, the cells lysed within the droplet and released polyadenylated RNA bound to the barcoded bead that was captured with the cell. Following the user manual, the droplets were directly subjected to reverse transcription, the emulsion was broken, and cDNA was purified using Dynabeads MyOne Silane (Thermo Fisher Scientific). After amplifying cDNA with ten cycles, it underwent purification and a quality control check on the Fragment Analyzer. The cDNA was fragmented for 5 min and dA-tailed, followed by an adapter ligation step and an indexing PCR of ten cycles in order to generate libraries. After quantification, the libraries were sequenced on an Illumina NextSeq 500 machine using a high-output flowcell in PE mode (R1: 26 cycles; I1: 8 cycles; R2: 57 cycles), thus generating at least 220 million fragments per sample.

#### Preprocessing and cluster detection

Data analysis was done using the scanpy python package^103^ (v.1.3.1). Cells with fewer than 200 or more than 2500 expressed genes, or more than 4% reads mapped to mitochondrial genes, were discarded. The count data was normalized according to the scanpy tutorial, and highly variable genes were detected using the standard parameters and kept for downstream analysis. Finally, the data was log-transformed, variation factors were regressed out (number of genes and percentage of mitochondrial genes) and scaled in accordance to the scanpy tutorial. Dimensionality was reduced using principal component analysis. In this way, a neighborhood graph was calculated using ten neighbors and 40 principal components. Following a Uniform Manifold Approximation and Projection (UMAP) embedding, cell clusters were detected based on the Louvain clustering implementation of scanpy using a resolution parameter of two, with the aim to detect smaller clusters.

#### Cluster annotation

HRO single-cell analysis by 10X genomics yielded 4031 genes across 6665 cells that were used for cell cluster detection and annotation (Fig. 1h and Supplementary Fig. 4b–f). To functionally assign clusters to different cell types, GOI lists of known marker genes were assembled from published data^37,58,104–109^, aiming to discriminate the following cell types: cones, rods, MG, bipolar cells, and amacrine, horizontal, and retinal ganglion cells. The overlap of genes between GOI lists and single-cell data was taken as input for a dotplot representation in scanpy (v.1.4.1), where the expression of the selected marker genes across all Louvain clusters was analyzed. The local expression pattern of these marker genes was additionally plotted onto the UMAP embedding; Louvain clusters were assigned to a specific cell type based on their respective GOI expression pattern. Two clusters found in this control sample expressed genes of several photoreceptor GOI lists and were therefore assigned as immature photoreceptors. To infer the cell-type-specific composition of the retinal organoid, the number of cells in the cluster was counted and summed up for all clusters corresponding to each specific cell type.

#### Foveal and peripheral expression pattern analysis

To infer whether HRO-derived photoreceptors and MG cells are closer to a foveal or peripheral expression pattern, the correlation between three reference single-cell datasets and our photoreceptors and MG cells was calculated. Published datasets from three healthy human individuals were retrieved^58^. Expression data of annotated rods, cones, and MG were extracted and used for comparison. HRO data: *N* = 1 with the following number of cells: 1692 rods, 1864 cones, 1666 MG. Human reference data: *N* = 3 individuals with the following total number of cells: Foveal samples: 238 rods, 225 cones, 891 MG. Peripheral samples: 1318 rods, 79 cones, 1522 MG. Mean expression vectors of peripheral cones, peripheral rods, foveal cones, foveal rods, peripheral MG, and foveal MG were calculated from the reference datasets. Since each human dataset contained samples from three donors, 18 mean vectors were calculated in the total. Subsequently, the intersection of the genes detected in the reference datasets and the HRO single-cell data was used for computing correlations. The HRO single-cell data were log10(x + 1) transformed to match the scale of the reference data. Finally, the Pearson correlation was calculated for each individual rod, cone, and MG cell against all previously derived mean expression vectors of the reference datasets across all donors. To furthermore highlight the correlation trends toward the fovea or periphery, the distribution of correlation delta values was calculated per cell type and donor.

### Statistical analysis and data presentation

Sample sizes were chosen based on our own preliminary studies. All samples were included in the analysis. Organoids were randomly assigned to experimental groups. Data was documented with Microsoft Excel (version 16.64), and statistical analysis was performed with GraphPad Prism 8 software: to compare the means of three or more groups, a one-way analysis of variance (ANOVA) was used, followed by Tukey’s post hoc multiple comparison test. Reported *P* values were adjusted for multiple comparisons for experiments with three or more groups. To compare the means of two normally-distributed groups, an unpaired two-sided Student’s *t* test was used. Results were considered significant for *P* < 0.05; data were plotted as mean and standard deviation. Mean and standard deviations (SD) were computed for total organoid numbers (*n*) from *N* experiments, unless stated otherwise. Unless otherwise indicated, statistics were analyzed for *N* ≥ 3 independent experiments, with *n* ≥ 5 organoids per variable. Boxplot: 50% of the data are represented by the box and the upper and lower 25% by the lines. Median (box notch), mean (rhomb), and outliers (dots) are shown. Violin graph shows the median (thick line), quartiles (dotted lines), and is based on max/min values.

### Image processing and figure preparation

Data graphs and schematic illustrations were prepared using GraphPad Prism 8 and Adobe Illustrator CC (version 26.5) software, respectively. Images were optimized by making minor changes to contrast, and cropped in Adobe Photoshop CC (version 23.5.0) and arranged using Adobe Illustrator CC. Heatmaps, boxplots, and data analyses were generated for transcriptomics data using the R package pheatmap (1.0.12). Venn diagrams and tables of expressed genes overlapping the genes of the GOI lists were generated using R. All single-cell-related figures were generated using the visualization methods provided by the scanpy package. Only the Pearson correlation plots were visualized in R using the ggplot2 package (3.1.0).

### Reporting summary

Further information on research design is available in the Nature Research Reporting Summary linked to this article.

## Supplementary information

Supplementary Information

Description of Additional Supplementary Files

Supplementary Data 1

Supplementary Data 2

Supplementary Data 3

Supplementary Data 4

Supplementary Data 5

Supplementary Data 6

Supplementary Data 7

Supplementary Movie 1

Supplementary Movie 2

Supplementary Movie 3

Supplementary Movie 4

Reporting Summary

## Data Availability

The authors declare that all data supporting the findings of this study are available within the paper and its Supplementary information files and no restrictions apply. The next-generation sequencing data that supports the findings of the study have been deposited on Gene Expression Omnibus: the accession code is GSE146641 for the raw bulk RNA-seq data and GSE174215 for single-cell RNA-Seq data; there, we also provide the uap workflow including all parameters and software used in the supplement. RNA sequencing reads were aligned to the human genome using a publicly available dataset (UCSC Human Dec. 2013 GRCh38/hg38 Assembly). Source data are provided with this paper.

## Source data

Source Data

## Code availability

The in-house Fiji (version 2.3.0) macro developed for photoreceptor pattern analysis on en-face retinal images was deposited at GitHub repository with the identifier (https://github.com/felwag/photoreceptor-pattern-analysis and 10.5281/zenodo.7040860)^84^ together with related resources and example files. The in-house Fiji macro developed for cell-delamination analyses on images of retinal cross-sections were deposited at Zenodo repository together with related resources and example files [10.5281/zenodo.5188650]^83^.
