# Supplementary material for: HBEGF-TNF induce a complex outer retinal pathology with photoreceptor cell extrusion in human organoids
Source: Nat Commun. 2022 Oct 19;13:6183. doi: 10.1038/s41467-022-33848-y (PMC9581928; doi:10.1038/s41467-022-33848-y)
Supplement: Supplementary file 1 — Supplementary Information [file 41467_2022_33848_MOESM1_ESM.pdf]

SUPPLEMENTARY INFORMATION

Title:

HBEGF-TNF induce a complex outer retinal pathology with photoreceptor cell extrusion in human organoids

Author list

Manuela Völkner 1, 2; Felix Wagner 1, 2; Lisa Maria Steinheuer 3; Madalena Carido 1; Thomas Kurth 4; Ali Yazbeck 3; Jana Schor 3; Stephanie Wieneke 2; Lynn J. A. Ebner 2; Claudia Del Toro Runzer 2; David Taborsky 2; Katja Zoschke 2; Marlen Vogt 1; Sebastian Canzler 3; Andreas Hermann 1, 2, 5; Shahryar Khattak 1, 4; Jörg Hackermüller 3, 6 & Mike O. Karl 1, 2 *

Affiliations

1 Center for Regenerative Therapies Dresden (CRTD), Technische Universität Dresden, Dresden, Germany

2 German Center for Neurodegenerative Diseases (DZNE), Dresden, Germany

3 Department Computational Biology, Helmholtz Centre for Environmental Research – UFZ, Leipzig, Germany

4 Technische Universität Dresden, Center for Molecular and Cellular Bioengineering (CMCB), Technology Platform, Germany

5 Department of Neurology, Technische Universität Dresden, 01307 Dresden, Germany

6 Department of Computer Science, Leipzig University, Germany

* Lead contact / Correspondence to: mike_o.karl@tu-dresden.de

Table of contents:

Supplementary Figures 1 to 19

Supplementary Movies 1 to 4

Supplementary References 1–46

Supplementary Figure 1

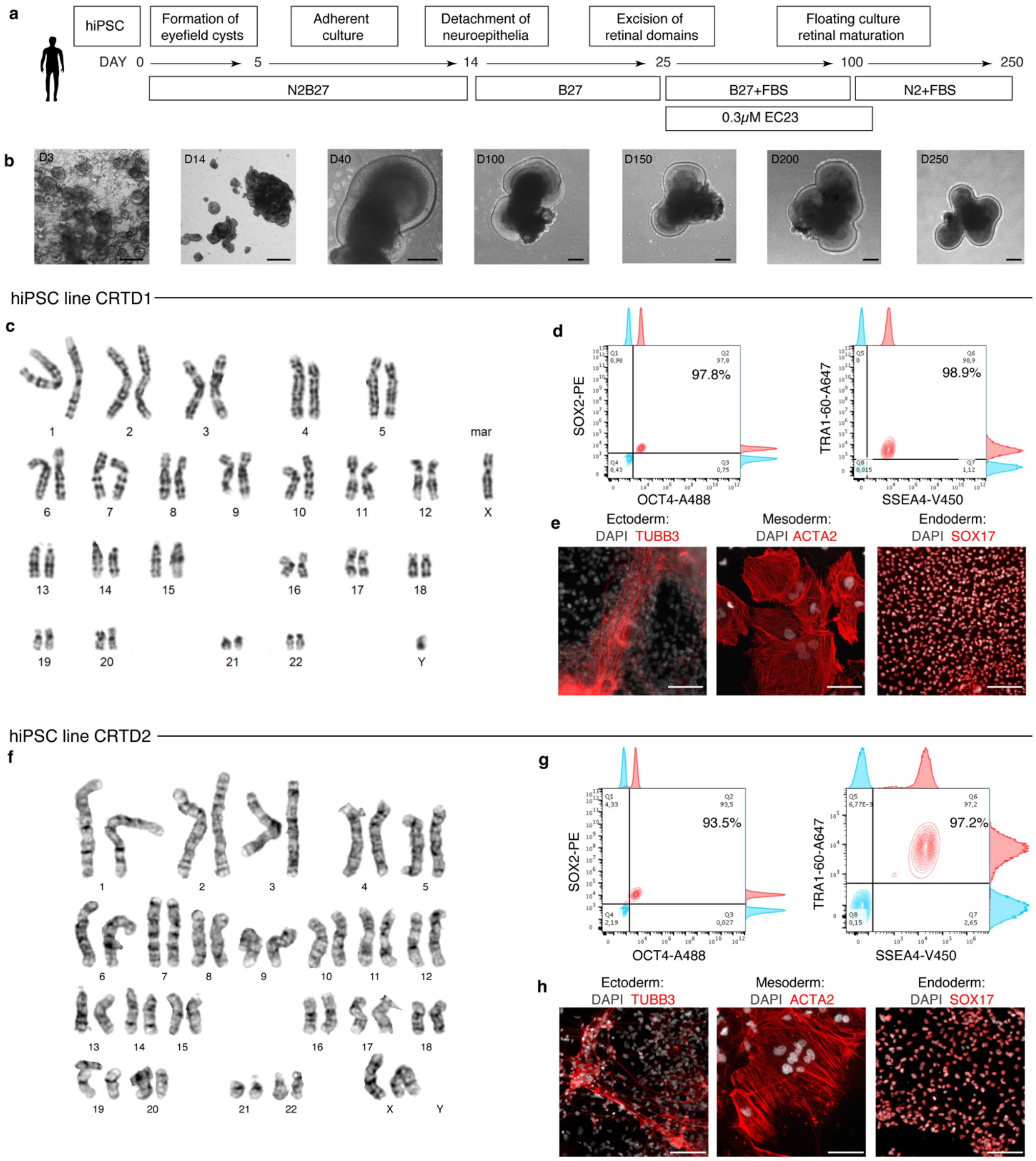

i

hiPSC line	Gender	Cell of origin	Donor	Reprogramming method	Reference
CRTD1	Male	Foreskin fibroblasts	Healthy	Non-integrating sendai virus, OCT3/4, SOX2, KLF4, cMYC	this study
CRTD2	Female	CD34+ peripheral blood	Healthy	Non-integrating sendai virus, OCT3/4, SOX2, KLF4, cMYC	Sürün et al. 2020
5A	Male	Skin fibroblasts	Healthy	Retroviral transduction, OCT4, SOX2, KLF4, cMYC	Lojewski, X. et al. 2014
IMR90	Female	Fetal lung fibroblasts	Healthy	Lentiviral transduction, OCT4, SOX2, NANOG, LIN28	Yu, J. et al. 2007

Supplementary Figure 1: Generation of human retinal organoids (HROs) from published and newly-established (CRTD1 and CRTD2) hiPSC lines. Data supplements Fig. 1. (a) Schematic overview of the cyst-based protocol for HRO differentiation. (b) Representative phase-contrast images of HROs at different developmental (D3–100) and postmitotic (D150–250) stages. N=13 independent experiments (N) with ≥50 HROs (n) per N, using 4 different hiPSC lines. (c-h) Characterization of CRTD1 and CRTD2 hiPSC lines: CRTD1 hiPSCs were derived from foreskin fibroblasts and CRTD2 from CD34+ peripheral blood cells reprogrammed using CytoTune-iPS 2.0 Sendai Reprogramming Kit (see methods). (c, f) Karyotype of CRTD1 and CRTD2 hiPSC is normal. G-banding karyogram is shown. (d, g) CRTD1 and CRTD2 hiPSCs express high levels of pluripotency markers OCT4, SOX2, SSEA4, and TRA-1-60 as determined by flow cytometry. This was also confirmed by real-time PCR for key markers of pluripotency according to the International Stem Cell Initiative (ISCI) using the human ES cell Primer Array from Takara Clontech (not shown). (e, h) Trilineage spontaneous differentiation of the CRTD1 and CRTD2 hiPSC line was confirmed using immunocytochemistry for ectoderm (TUBB3), mesoderm (ACTA2), and endoderm (SOX17) markers, as well as by qRT-PCR by utilizing the human ES cell Primer Array from Takara Clontech (not shown). N=1 independent experiment (N). (i) Summary table: hiPSC lines used in this study. Scale bars: (b) 200 µm; (e, h) 100 µm.

Supplementary Figure 2

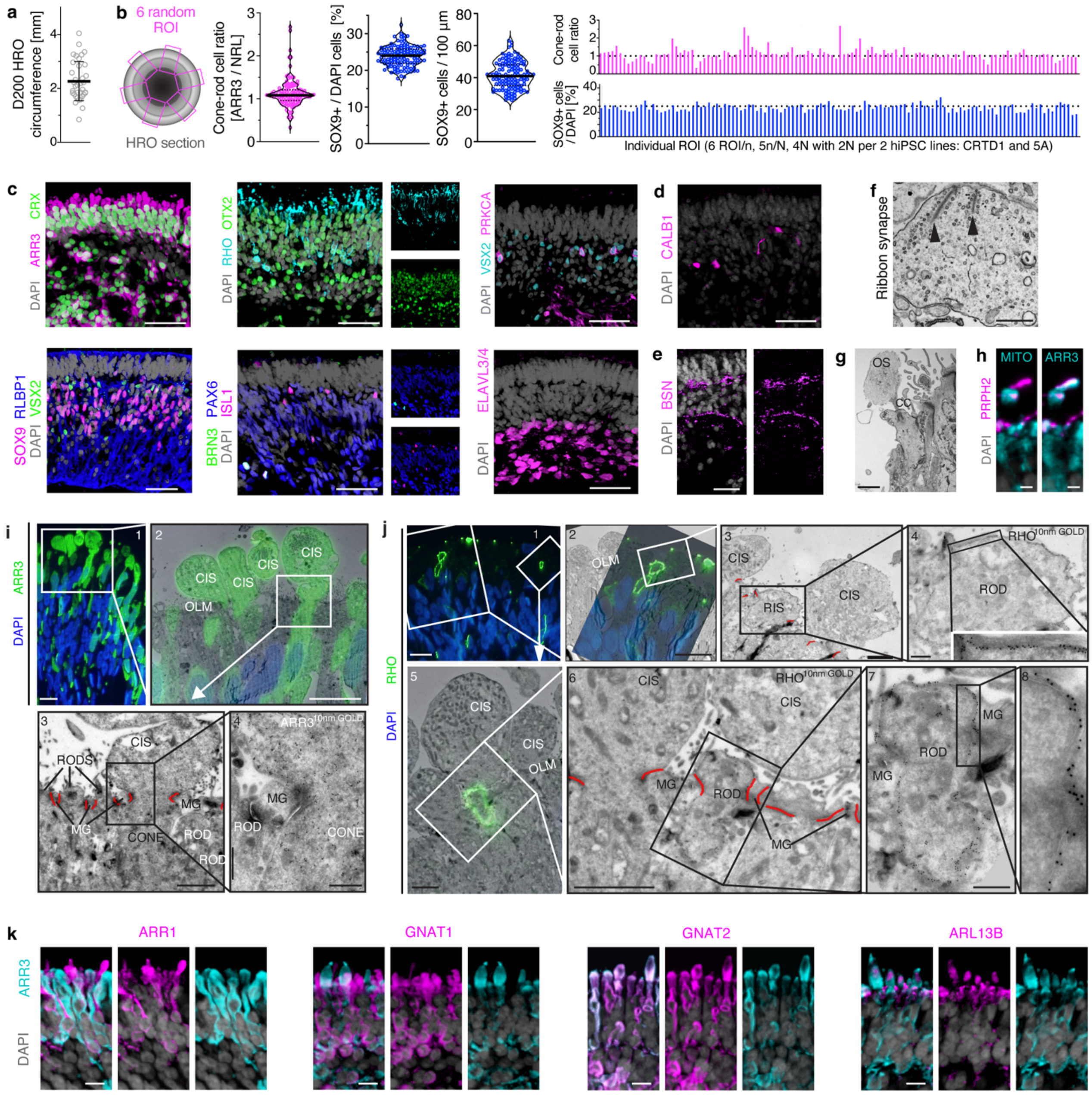

Supplementary Figure 2. Further characterization of the HRO-cell composition. Data supplements Fig. 1. (a) HRO circumference was measured on images of immunostained HRO sections. Circles represent individual HROs (n) derived from 4 independent experiments (N) (n≥5/N). Graph shows mean ±SD over n. (b) To analyze homogeneity of rod and cone, as well as MG cell distribution within an HRO, sections were stained for NRL/ARR3/DAPI or SOX9/DAPI, respectively, and 6 ROIs were analyzed at different random positions within one central HRO section per HRO (n). Violin graphs show median (thick line), quartiles (dotted lines), and are based on max/min values.; each circle represents a ROI. In bar graphs each bar represents one ROI; ROIs of the same HRO are plotted next to each other. N=5 HROs were analyzed per experiment (6

ROIs each) for a total of N=4 independent experiments (N) derived from 2 hiPSC lines (5A and CRTD1, N=2 each). (c) Postmitotic (D200) HROs were immunostained for retinal cell-type markers: photoreceptors: CRX and OTX2. Cones: ARR3. Rods: rhodopsin (RHO). Bipolars: PRKCA, VSX2, OTX2, ISL1. Amacrines: ELAVL3/4, PAX6, ISL1. Ganglion cells: BRN3, PAX6, ELAVL3/4. Müller glia (MG): SOX9, RLBP1. Photoreceptors are mainly localized in an outer nuclear layer-like structure on the outer (apical) side of the organoid. Interneurons (bipolars, amacrines) and MG form an intermediate, inner nuclear layer-like structure. MG processes are organized radially, spanning the entire epithelial width. (d) CALB1 is only detected in inner neurons and not in photoreceptors in the outer nuclear-like layer in HROs, like in the human macula, whereas CALB1 is expressed in photoreceptors in the human peripheral retina[1]. This further supports a macula-like identity of cones in the HRO system. (e) Immunostaining for the synaptic marker bassoon (BSN) indicates the formation of outer and inner plexiform layers in HROs. (c-e) N=2 independent experiments (N) with n=5 HRO/N. (f-g) TEM confirmed the formation of ribbon synapses (f) and indicates the formation of nascent photoreceptor outer segments (g) (POS, with connecting cilium; cc). N=1 independent experiments (N) with n=3 HRO. (h) Immunohistochemistry confirmed that (cone (ARR3)) photoreceptors showed the typical structure of mitochondria-rich (MITO) inner segments, and a more apical localized outer segment indicated by expression of peripherin (PRPH2). N=1 independent experiments (N) with n=6 HROs. (i-j) Correlative light and electron microscopy (CLEM) analysis confirmed formation of cone (ARR3) and rod (RHO) photoreceptors with photoreceptor inner segments in control HROs (D200); staining visualized simultaneously by immunofluorescence and gold particles (black dots, 10 nm gold particles). DAPI labels cell nuclei. The cell junctions (red pseudocolor) between photoreceptors (P) and Müller glia (MG) form an outer limiting membrane (OLM). Cone (CIS) and rod (RIS) photoreceptor inner segments, stained positive for ARR3 and RHO respectively, extend apically to the OLM. Together, the photoreceptor cellular ultrastructure, with prominent photoreceptor inner segments (PIS) and characteristic mitochondria, suggest a neonate maturation status[2, 3]. N=1 independent experiments (N) with n=3 HROs. (k) Markers indicative for mature rods (ARR1, GNAT1) and cones (ARR3, GNAT2), as well as ciliary markers (ARL13B), are expressed in HROs. N=1 independent experiments (N) with n=6 HROs (n). Scale bars: (c, d) 50 µm, (f, i4) 500 nm, (g, h) 1 µm, (i1,2, k) 10 µm, (i3) 2 µm, (j1,5) 5 µm, (j2,4,6) 1 µm, (j3,7) 200 nm. Source data are provided as a Source Data file.

Supplementary Figure 3

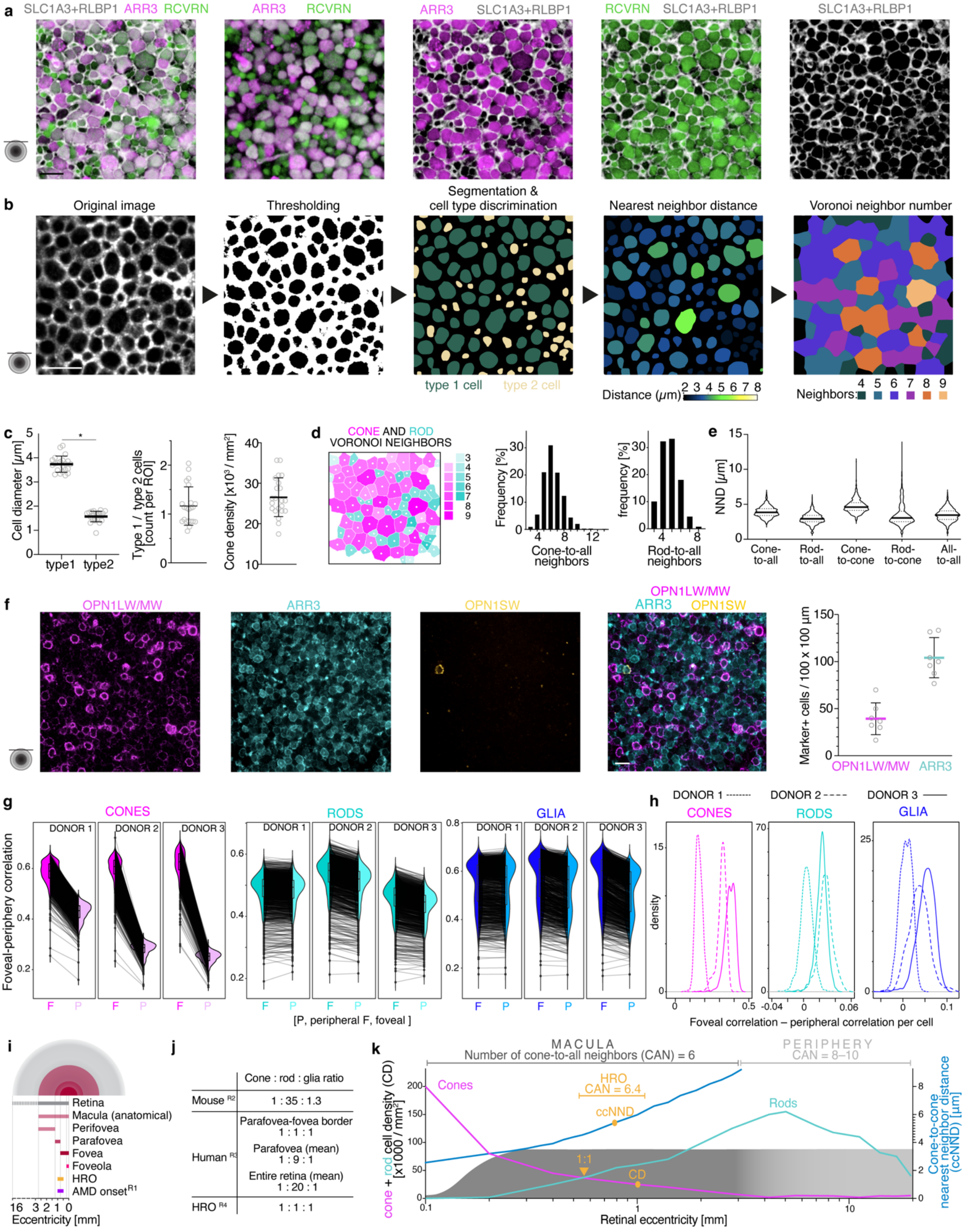

Supplementary Figure 3. Cellular and molecular analysis of the photoreceptor composition in HROs.

Data supplements Fig.1. (a) En-face images at the outer limiting membrane (OLM) level of immunostained HRO wholemounts revealed different sizes of photoreceptor inner segments (RCVRN): indicating larger cone

(RCVRN+ARR3+) and smaller rod (RCVRN+ARR3–) segments. The OLM was identified using the Müller glia markers SLC1A3 and RLBP1 as a proxy. N=1 independent experiments (N) with n=7 HROs (n), 1 region of interest/n. (b) The photoreceptor pattern was investigated using automated FIJI analysis (CLIJx tool, see methods) of en-face images of HRO wholemounts immunostained for SLC1A3+RLBP1[4]. Manual thresholding was followed by automated image segmentation, cell-type classification into a larger (presumably cones) and a smaller subpopulation (presumably rods), and analysis of cell sizes, neighbor distances and number of voronoi neighbors. To analyze cone-to-cone neighbor distances and number of cone neighbors, rods were excluded after cell-type classification and vice versa for rod analysis. N=1 independent experiment with n=9 HROs (n), 3 region of interest/n. (c) A larger (type 1) and a smaller (type 2) subpopulation of cells could be separated with a mean ratio of 1.1 to 1. Assuming that the type 1 cells represent cones (see Supplementary Fig. 3a-b), cone density per mm^2 was assessed. Graphs: Each circle represents a region of interest (ROI); 9 HROs (n)/N, 3 ROIs/n, N=1 independent experiments. Graphs shows mean ±SD and statistics over n (two-sided Student's t-test; * $p<0.0001$). (d-e) Machine learning (Fig. 1d) identified two cell types with parameters as observed for cones and rods[5]. Comparable to patterning studies of the human retina in vivo[6-8], (d) the number of voronoi neighbors, as well as (e) nearest neighbor distances (NND) were assessed for cones and rods of HROs (>630 cells analyzed per cell type). Violin graphs show the median (thick line), quartiles (dotted lines), and are based on max/min values. (f) In the human retina opsins are expressed in rods (RHO) and all cone subtypes[9]. Representative images and quantitative analysis of the number of cone photoreceptor (ARR3) subtypes based on opsin expression (OPN1LW/MW, OPN1SW) in immunostained organoid wholemounts imaged at the level of photoreceptor nuclei (n=7 HROs, N=1 independent experiments; graphs show mean ±SD over n, circles represent individual n). OPN1LW/MW was widely observed, while OPN1SW cells were rarely present. (g-h) Single-cell RNA-seq based comparison of the cone, rod, and MG cell transcriptomes of this HRO system with published data from three individual human donor retinal fovea and periphery samples[10]. Related to Fig. 1h-i; Supplementary Fig. 4a. (g) The violin graphs show the Pearson correlation of HRO cones, rods, and MG against the human reference vectors of foveal and peripheral cells across all three donors. Paired data points (here cells) were connected with lines. HRO cones strongly correlate with a foveal transcriptome in all three donor samples. Rods and glia show a slightly higher correlation value with fovea than periphery in donors 2 and 3, whereas in donor 1 these transcriptomes are rather in-between foveal and periphery. However, a clear majority of rod and MG cells is either more foveal or show transcriptomes in-between fovea and periphery. Thus, we observed no mixture of more foveal and more peripheral-related cells, which is confirmed by quantitative analysis: (h) Distribution of the difference

between foveal and peripheral correlation values in HRO-cell cones across all donors. Donors are indicated by the differently dashed lines as shown. The density plots show the delta of HRO cone, rod, and MG correlation values with foveal and peripheral expression vectors. Negative or positive values indicate a trend towards periphery or fovea, respectively. The observed variance between donors might be due to the sampling procedure: targeting the macula precisely without any standardized methods or a precise manipulator is not trivial. Of note, the human donor samples were a 2-mm foveal-centered punch and a 4-mm peripheral punch from the inferotemporal region. Thus, the foveal sample should ideally contain the entire foveola (cones only) and fovea, as well as about half of the parafovea (both with rods and cones): however, if the foveola is not precisely centered, it is easy to collect more parafovea than fovea and thus more rods. Boxplots within violin graphs depict: minimum, 1st quantile, median, 3rd quantile, maximum values, the dots are outlier data points of the boxplot (lower range is the 25%th percentile-1.5×IQR and upper range is 75%th percentile+1.5×IQR, where IQR is the inter-quartile range or distance between the first and third quartiles). The HRO data are based on N=1 independent experiments (N) with the number of cells (n) given in the legend of Fig. 1h. Human reference data are based on N=3 independent individuals with the number of cells (n) given in the source data. (i) Schematic of the anatomical macula of the human retina, which is radially subdivided into different subregions with unique retinal cell spatial organizations, densities (see Fig. 1i), and ratios. Retinal subregions and cell composition in Fig. 1i were adapted from references[11, 12]. Of note, the anatomical fovea is equivalent to the clinical macula, which represents the innermost 1.5 mm diameter of the entire anatomical macula. Macular degenerative diseases (MDD), including age-related macular degeneration (AMD), Stargardt and Best diseases, most frequently commence in the parafoveal region (reference (R): R1, [13-15]). (j) Comparison of retinal cell-type ratios observed in the human and mouse retina, as well as in the HROs, confirming a human macula-like cone-rod-glia cell ratio in the HROs. References (R) in Table: R2, [16]; R3, [9, 17-19]; R4, HRO based on Fig. 1c. (k) Schematic comparison of HROs with the human retina: based on the analysis shown here, the HRO system (indicated in yellow) contains many characteristics of the human retina, including several key features of the photoreceptor pattern characteristics for the human macula region, but not periphery: a 1:1:1 cone, rod, and MG cell composition[5, 11, 19-21] at low variance[22], similar high cone densities (CD)[6, 18, 22-24] and cone-to-cone nearest neighbor distance (ccNND)[24], and a hexagonal cone pattern based on cone-to-all neighbors (CAN)[6-8, 25-27]. Plotted lines represent cone and rod patterning data from the human in-vivo retina. Shape of the human retina depending on eccentricity is shown in gray, with the foveal pit and slope on the left. Scale bars: (a-b, f) 10 μm. Source data are provided as a Source Data file.

Supplementary Figure 4

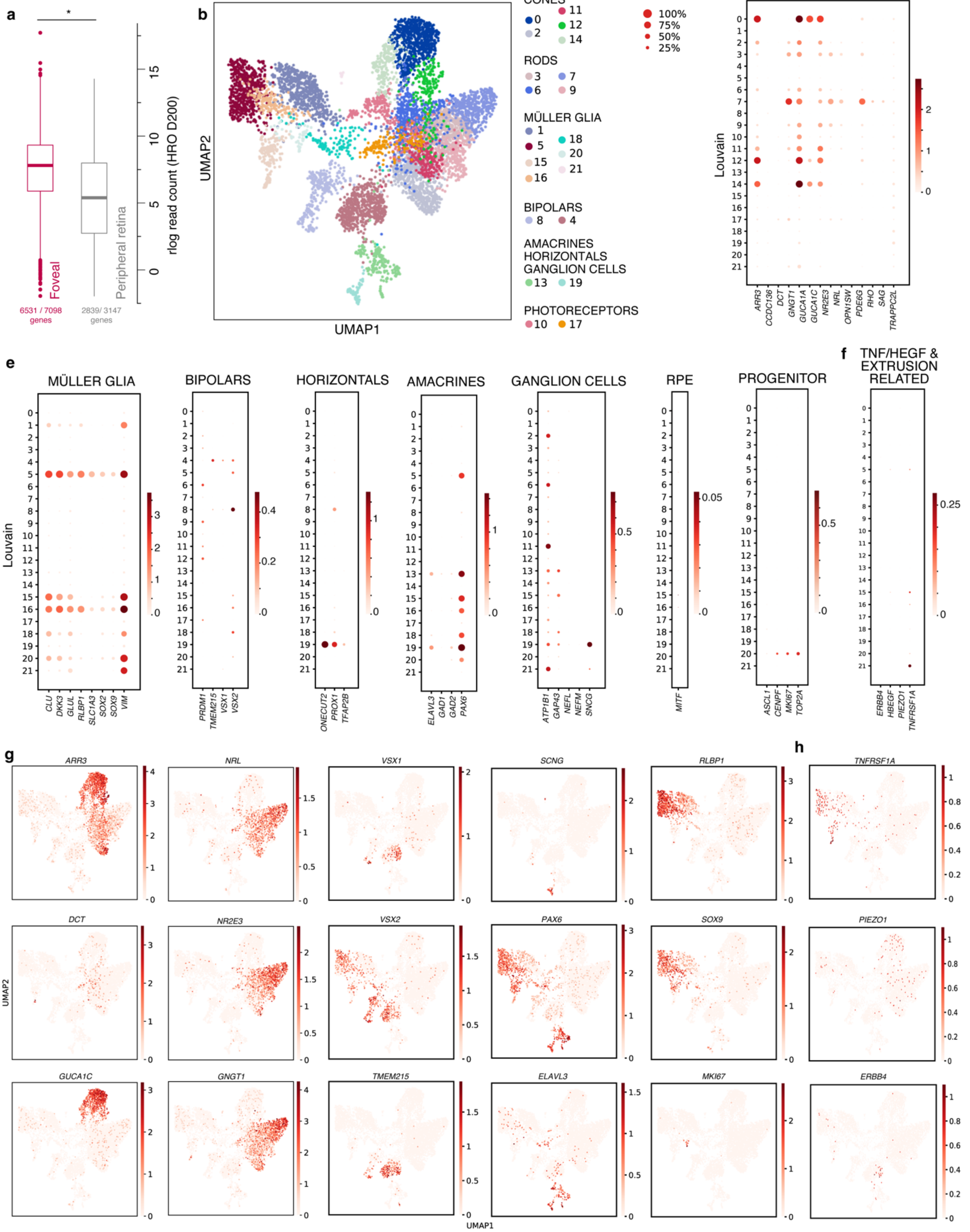

Supplementary Figure 4: Transcriptome analysis of the HRO system: bulk and single-cell RNA-seq.

Data supplements Fig. 1. (a) Comparison of the summarized expression of genes in D210 HROs (bulk RNA-seq, n=6 individual entire HROs) that had previously been identified as being expressed in samples of the foveal[28] and peripheral retina region[29-31] in healthy patients. The n=6531 genes that overlap between HRO and

foveal genes (6531 out of 7098 genes, ≈92%) show significantly higher expression (two-sided Student's t-test, * p=7.06505065385364e-178) than those n=2839 genes that overlap between HRO and peripheral retina (2839 out of 3147 genes, ≈90%). The total number of expressed genes in these HRO samples is 30,542. The boxplots summarise rlog-transformed read counts in HROs (median expression per gene of n=6 individual HRO samples of D200): The measure of center is the median of the expression level of all genes per retina region. The lower and upper hinges correspond to the first and third quartiles (the 25th and 75th percentiles). The upper whisker extends from the hinge to the largest value no further than 1.5×IQR from the hinge (where IQR is the inter-quartile range). The lower whisker extends from the hinge to the smallest value, at most 1.5×IQR of the hinge. Data beyond the end of the whiskers are called outlier and plotted individually. (b-h) Single-cell RNA-seq analysis of the HRO system: (b) After filtering, 4031 genes across 6665 cells (n) were used as a basis for cell cluster detection and annotation. As depicted in the dotplot, a total of 22 Louvain clusters were detected in a UMAP embedding using an increased resolution parameter. Sizes ranged from 665 cells in cluster 0, to ten cells in cluster 21. Clusters were assigned to major retinal cell types based on specific marker genes (see (c-e)) as indicated in the figure: mature cones and rods (right) as well as Müller glia cells (left) were clearly separated from the other cell types (cell types grouped as summarized in Fig. 1h). Bipolars, as well as amacrines, horizontal cells, and ganglion cells were detected on the bottom of the plot. Premature photoreceptors (light pink) were found in between mature rods and cones. All clusters assigned to a single cell type were merged to obtain the labeling in Fig. 1h. (c) Legends for dotplots in (d-f): dot size indicates the abundance of cells expressing each gene, and the color gradient corresponds to the expression level within each cell indicated by each heatmap legend. (d-e) Expression of selected marker genes used to assign clusters to major cell types (see (b)). (f) Selected genes related to the HT-HRO model are expressed in HRO. *TNF*, *TNFR2B*, *EGFR* and *PIEZO2* were not detectable in D200 HRO cells. (d-f) The dotplots shows the mean log(x+1) expression of selected genes across the Louvain clusters in the HRO sample. Analysis for genes associated with retinal pigment epithelium (RPE) and (retinal) progenitors indicate absence of RPE and completion of retinal development (some example genes depicted). (g-h) Examples of UMAP embeddings of single-cell HRO sample: (g) Expression patterns of selected marker genes used to assign cell-cluster identity (see (d-e) for the related major retinal cell types) across all cells. (h) Expression patterns of some of the selected genes shown in (f). (g-h) The color gradient corresponds to the log(x+1) expression of selected marker genes per cell. The gene *MKI67*, encoding a nuclear protein associated with and perhaps necessary for cell proliferation, is not expressed in the majority of cells, indicating that all retinal cells in the HRO are in a postmitotic state. Data have been deposited on Gene Expression Omnibus.

Supplementary Figure 5

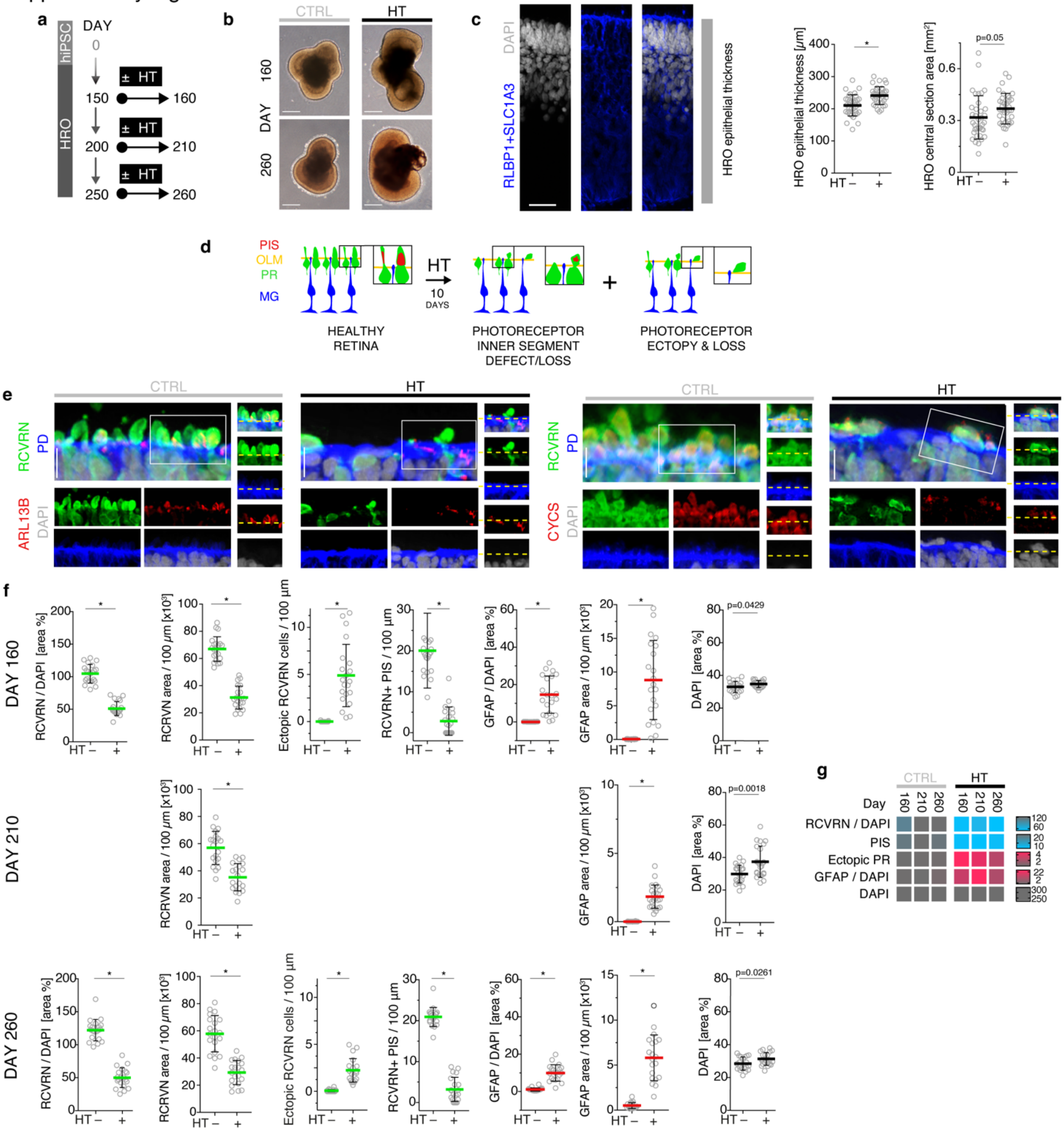

Supplementary Figure 5: HBEGF-TNF (HT) treatment of HROs at three different postmitotic timepoints causes comparable photoreceptor degeneration and reactive gliosis. Data supplements Fig. 2, 4. (a) Schematic of the experimental design for the HT treatment of HROs. HROs were treated daily with HT starting from D150, 200, or 250, and analyzed after 10 days of treatment. (b) Representative bright-field images of HROs in culture. HRO at D160, 210 and 260 were analyzed in N≥3 independent experiments (N), each with n≥5 HRO/N for each variable (CTRL vs. HT) at all 3 time points. (c) HRO tissue section area and epithelial thickness were measured on microscopic images of immunostained entire central HRO sections

(D210) using Fiji software. For all parameters, the apical organoid boundary was defined using DAPI immunostaining (as indicated in the HRO image), and the basal boundary was defined using RLBP1 and SLC1A3, as well as DAPI. Data were determined from 1 or 2 sections per individual HRO (n) derived from N=4 independent experiments (n≥5/N). (d) Drawing illustrates photoreceptor cell (green) localization and structure in the healthy retina and two photoreceptor histopathologies. Cell-cell contacts between Müller glia (blue) and photoreceptors form the outer limiting membrane (OLM, yellow line). Photoreceptors are substructured with their inner segments (PIS) located apically to the OLM. Loss of PIS are frequently observed in most retinal diseases, and photoreceptor displacement (ectopy) into the subretinal space (apically to the OLM), in AMD). (e) Representative images of immunostained HRO sections treated from D200 to 210 with HT or without. RCVRN+ PIS are localized outside the OLM, indicated by phalloidin (PD, labels filamentous actin). RCVRN+ PIS co-localize with ARL13B (cilia marker) and CYCS, known PIS components. HT treatment reduces the number of PISs and impairs their structure. Boxed ROIs show examples of HT-induced PIS defects and photoreceptor cell ectopy. Yellow dashed lines indicate the OLM. Extruded RCVRN+ photoreceptor cells were determined by a displaced cell nucleus positioned apically to the OLM (ectopic). (f) Quantitative analyses of total and ectopic photoreceptors (RCVRN+), and total cells (DAPI+ cell nuclei), RCVRN+ photoreceptor inner segments (PIS), and GFAP-positive areas (gliosis) in HROs. Graphs: Each circle represents one individual HRO (n) based on N≥3 independent experiments (N), each with n≥5/N. Representative images and data for D210 are shown in Fig. 2h-i; 4. (g) Heatmaps summarize quantitative data shown in (f). Heatmap scale according to data in (f). (c, f, g) Graphs ((c, f) mean ±SD; (g) mean) and statistics over n; p-values are from two-sided Student's t-test (* $p<0.0001$; for see Supplementary Data 2); circles represent individual n. Scale bars: (b) 500 μm, (c) 50 μm, (e) 10 μm. Source data are provided as a Source Data file.

Supplementary Figure 6

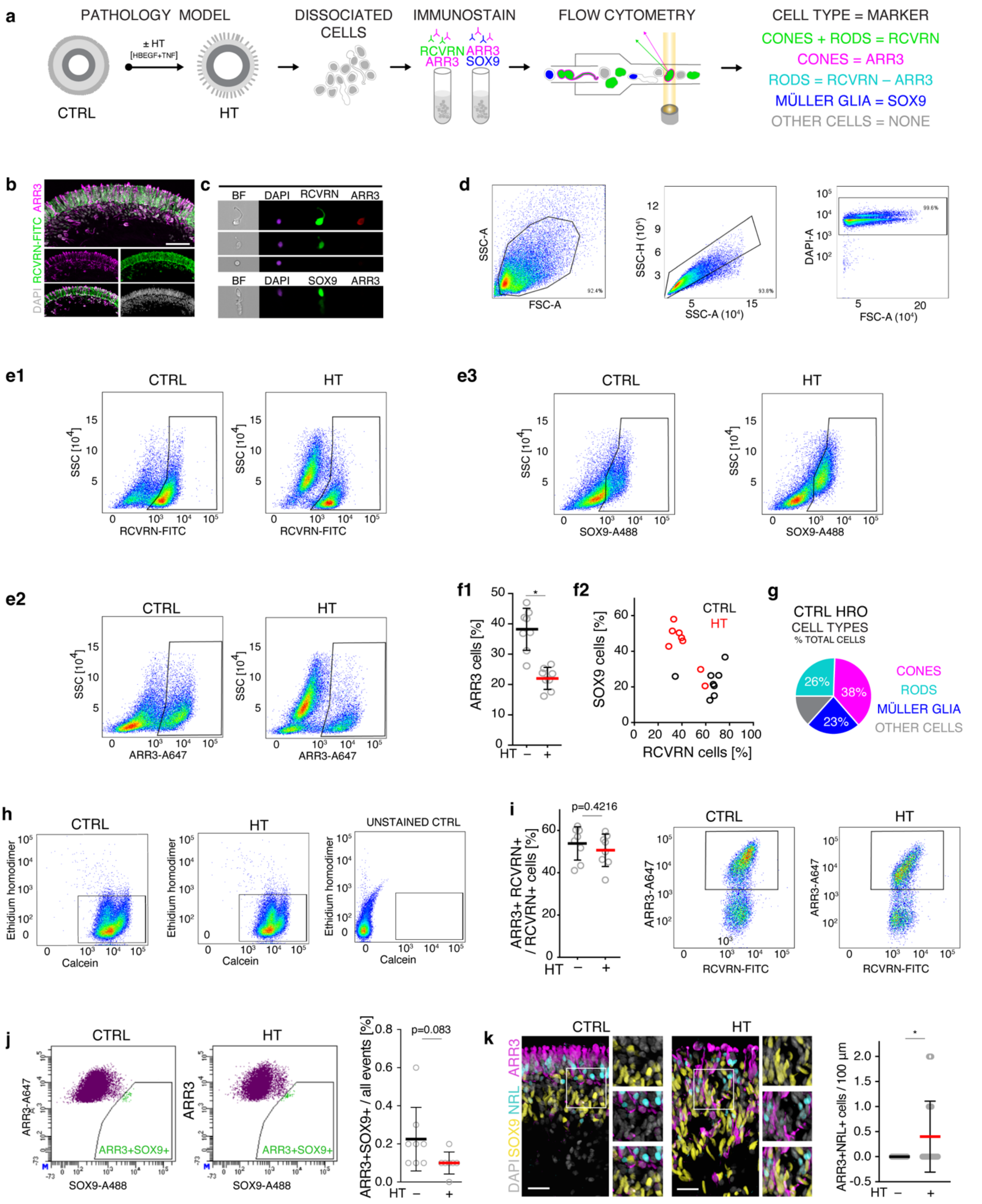

Supplementary Figure 6: Flow cytometry confirms the composition of HRO photoreceptors and MG cells. (a) Schematic: experimental design. Two sets of antibodies (RCVRN/ARR3 and ARR3/SOX9) were stained in parallel in each experiment. Comparisons of the cellular composition of CTRL or HT-treated HROs

after about 10 days. Cell types that can be identified by the respective antibody combinations are indicated. (b) HRO section immunostained with RCVRN-FITC and ARR3 antibodies shows differential labeling of photoreceptors in the outer nuclear layer: most cells are RCVRN+ and a subpopulation is co-stained with ARR3, indicative of cones. N=1 independent experiment with n=5 HROs (n). (c) Representative images of immunostained cells acquired by imaging flow cytometry to validate antibody specificities. Two out of eight set (s) of HROs were analyzed; data for each set was acquired from pooled samples with 6–9 HROs and 2–3s/N (N=2 independent experiments (N), 2 hiPSC lines). (d) Representative FACS blots showing the gating strategy: cell debris exclusion, cell doublets exclusion, and only nucleated (DAPI-positive) cells were included. (e-f) Dissociated control and HT-treated HROs were analyzed for cell-type composition by flow cytometry, using two sets of antibodies for (e1) RCVRN (photoreceptors) in combination with (e2, f1) ARR3 (cones), and ARR3 in combination with (e3) SOX9 (Müller glia) antibodies. (e1-3) Density plots: each dot on the plot represents an individual cell detected by flow cytometry; red/yellow/green/blue hotspots indicate increasing numbers of cells. Data in e1-2 are related to Fig. 2e1; f1 is related to Fig. 2e2. Data graphs and representative flow cytometry plots are shown. (f1) Cone cell number in HROs. Graphs: Each circle represents one set (s) of HROs; data for each set was acquired from pooled samples (s) with 6–9 HROs and 2–3s/N (s=8; N=3, independent experiments, 2 hiPSC lines). (f2) Correlation plot of RCVRN and SOX9 shows differential changes of HRO cell composition after HT treatment compared to CTRL. (g) Data summary based on a, e1, e2, f1, f2, and Fig. 2e. (h) Live-dead assay: Calcein staining confirms high cell viability in CTRL and HT samples. Related to Fig. 2f. (i) Flow cytometry analysis of cone (ARR3) cell fraction of total photoreceptors (RCVRN). Based on re-analysis of data derived from Fig. 2e1. (j-k) Validation of marker overlap by (j) flow cytometry (graph: each circle represents one set (s) of pooled HRO (n), 2–3s/N, N=3, 2 hiPSC lines, 32000–40000 cells analyzed), and (k) immunostaining analysis (graph: each circle represents one ROI, 2 ROIs/individual HRO (n), N=4 independent experiments (n=5/N)). Inset images show one enlarged region from the image on the left. The cone (ARR3) and Müller glia (SOX9) markers, used were very rarely detected in the same cells as the cone (ARR3) and rod (NRL) markers. DAPI, cell nuclei label; SSC, side scatter; FSC, forward scatter; A, area; H, height. Scale bar: 50 µm. (f1, g, k) Graphs ((f1, k) mean ±SD, (g) mean) and statistics over n; p-values are from Student's t-test; * $p<0.001$. Source data are provided as a Source Data file.

Supplementary Figure 7

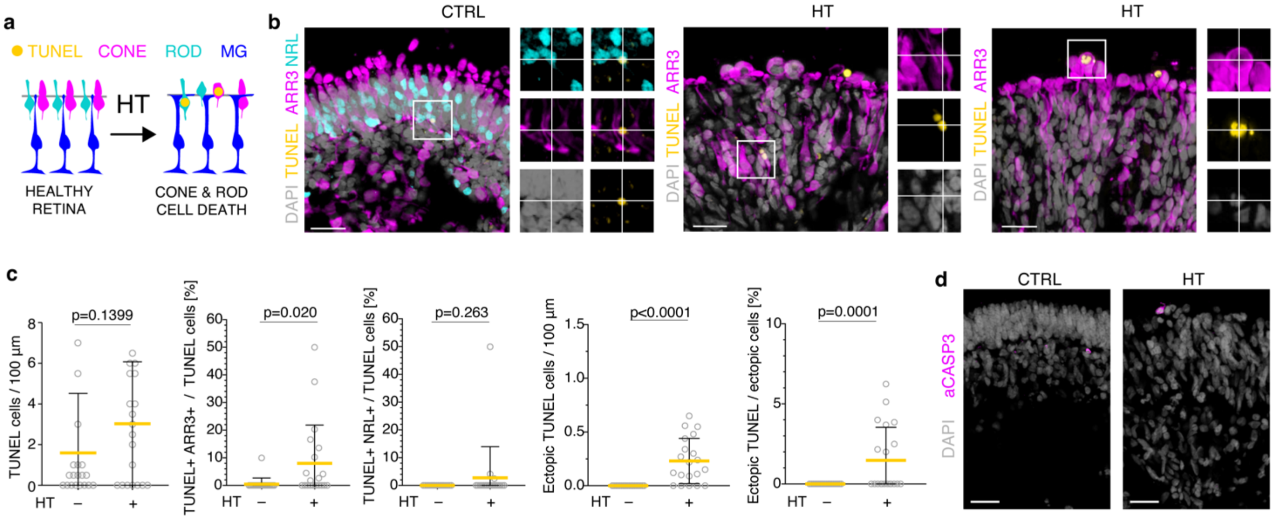

Supplementary Figure 7: Low cell death levels in control and HBEGF-TNF-treated HROs.

Data supplements Fig. 2. (a) Drawing depicts cell death as potential mode of photoreceptor loss. (b) Representative images of CTRL and HT-treated HRO tissue sections after 10 days, and (c) derived quantitative data of cone and rod cell death and ectopy. Immunostaining for ARR3, NRL, and DAPI was used to determine the number of cones, rods, and total cells, respectively. The number of dead cells was assessed using a TUNEL assay. White boxes indicate magnified ROIs depicted on the right of each image panel. Crosshairs highlight co-labeled cells of interest. Ectopic photoreceptors were identified based on ARR3 and NRL cell marker expression and displacement of their cell nuclei outside (apical to) the apical HRO epithelial border (see also Fig. 2-3; Supplementary Fig. 5). Graphs: Each circle represents one individual HRO (n) based on N=4 independent experiments (n=5/N). Graph (mean ±SD) and statistics (two-sided Student's t-test) over n (see Supplementary Data 2). (d) Representative images of CTRL and HT-treated HRO samples stained for activated CASP3 (aCASP3) and cell nuclei (DAPI). Qualitative analysis rarely showed any aCASP3-positive cells in HT-HROs or controls. N=2 independent experiments with n=5 HROs/N. Scale bars: (b) 50 µm; (d) 25 µm. Source data are provided as a Source Data file.

Supplementary Figure 8

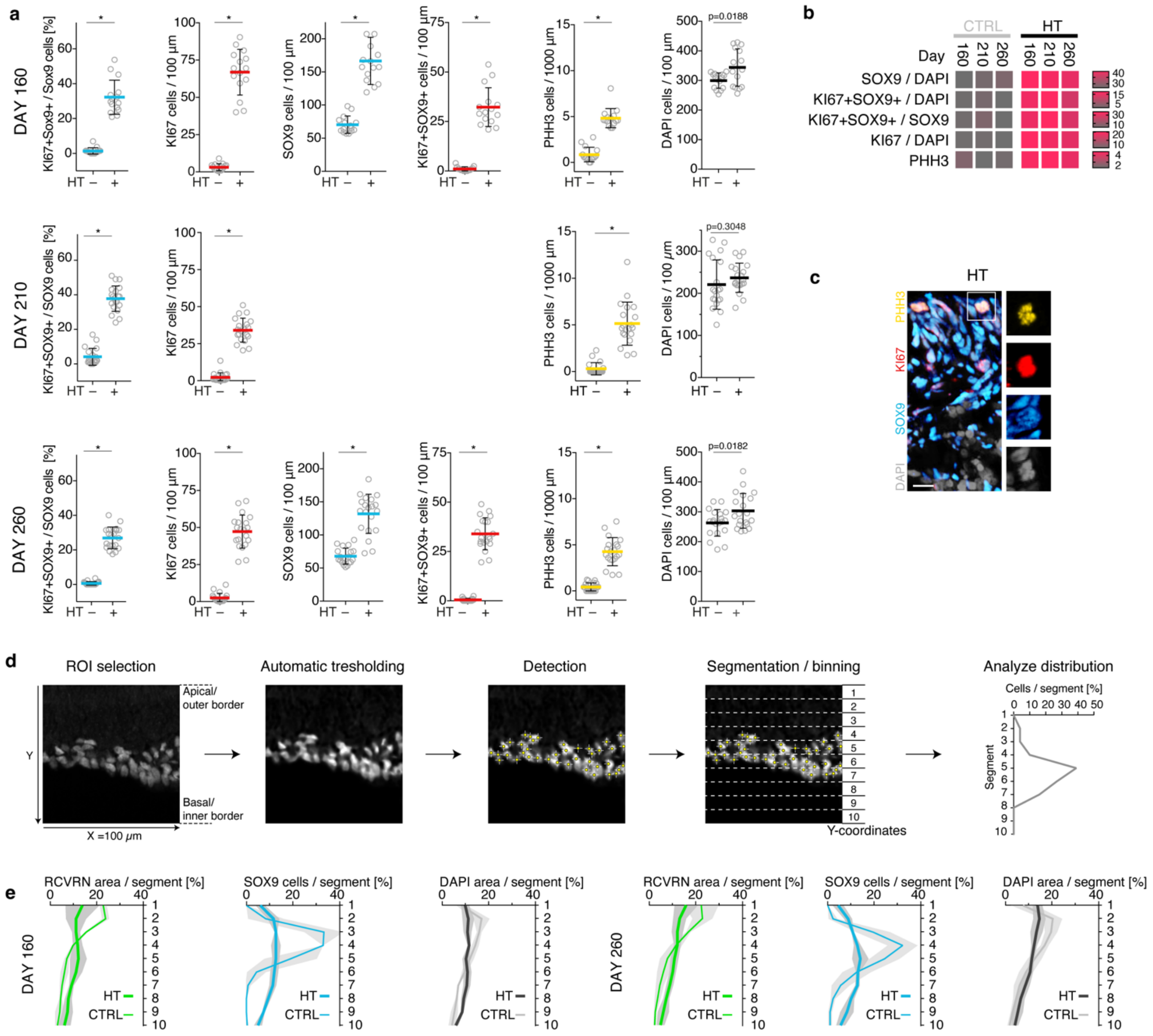

Supplementary Figure 8: HBEGF-TNF treatment of HROs at three different postmitotic timepoints causes comparable glial proliferation and retinal dyslamination. Data supplements Fig. 4. HROs were treated daily with HT starting from D150, 200, or 250 and analyzed after 10 days of treatment (D160, 210, 260). (a) Reactive (proliferative) gliosis was assessed by quantitative analysis of the gliosis hallmark GFAP, Müller glia (MG) (SOX9), cell proliferation (KI67), and mitosis (PHH3, phospho-histone H3) markers. Graphs: Each circle represents one individual HRO (n) based on N≥3 independent experiments (n≥5/N). Graph (mean ±SD) and statistics (two-sided Student's t-test, * p<0.001) over n (see Supplementary Data 2). (b) Heatmaps summarize quantitative data shown in (a). Heatmap scale according to data in (a). (c) Immunostained section of an HRO treated with HT for 10 days shows co-labeling of MG (SOX9), cell proliferation (KI67), and mitosis (PHH3, phospho-histone H3) markers indicating MG proliferation. (d) Workflow used for quantitative analysis of radial cell delamination, performed on microscopic images (ROIs 100 µm wide) of immunostained HRO serial sections. See also Methods for retinal dyslamination analysis. ROIs were positioned to include the entire epithelial layer, with the apical (outer) border of the tissue on top and the basal (inner) border at the bottom of the ROI. ROIs were automatically thresholded; the precise position (x/y coordinates) of positive signals was detected using Fiji software. Local maxima were detected (3D maxima finder, 3D ImageJ Suite) to determine the position of individual SOX9+ cell nuclei (MG). The position of each pixel above the threshold was determined for RCVRN (photoreceptors) and DAPI (all cell nuclei) signals. Then each ROI was divided into 10 equally-sized bins (segments) from the apical to basal organoid surface (y axis), and the relative number of detected local maxima or pixels was calculated for each bin (segment). To analyze the relative distribution of cells, the amount of positive signal (% of total signal per ROI) was plotted for each bin. (c-d) Three HRO ages, with N=3 (D160) or N=4 (D210, D260) independent experiments (n≥5 HROs (n)/N. (e) Quantification of radial cell delamination in control and HT-treated HROs at D160 and 260 (for D210 see Fig. 4f; experimental design: Supplementary Fig. 5a). Graphs show mean ±SD over n of N≥3 independent experiments (n≥5 HROs/N, 2 ROIs/n). Scale bars: (c) 10 µm. Source data are provided as a Source Data file.

Supplementary Figure 9

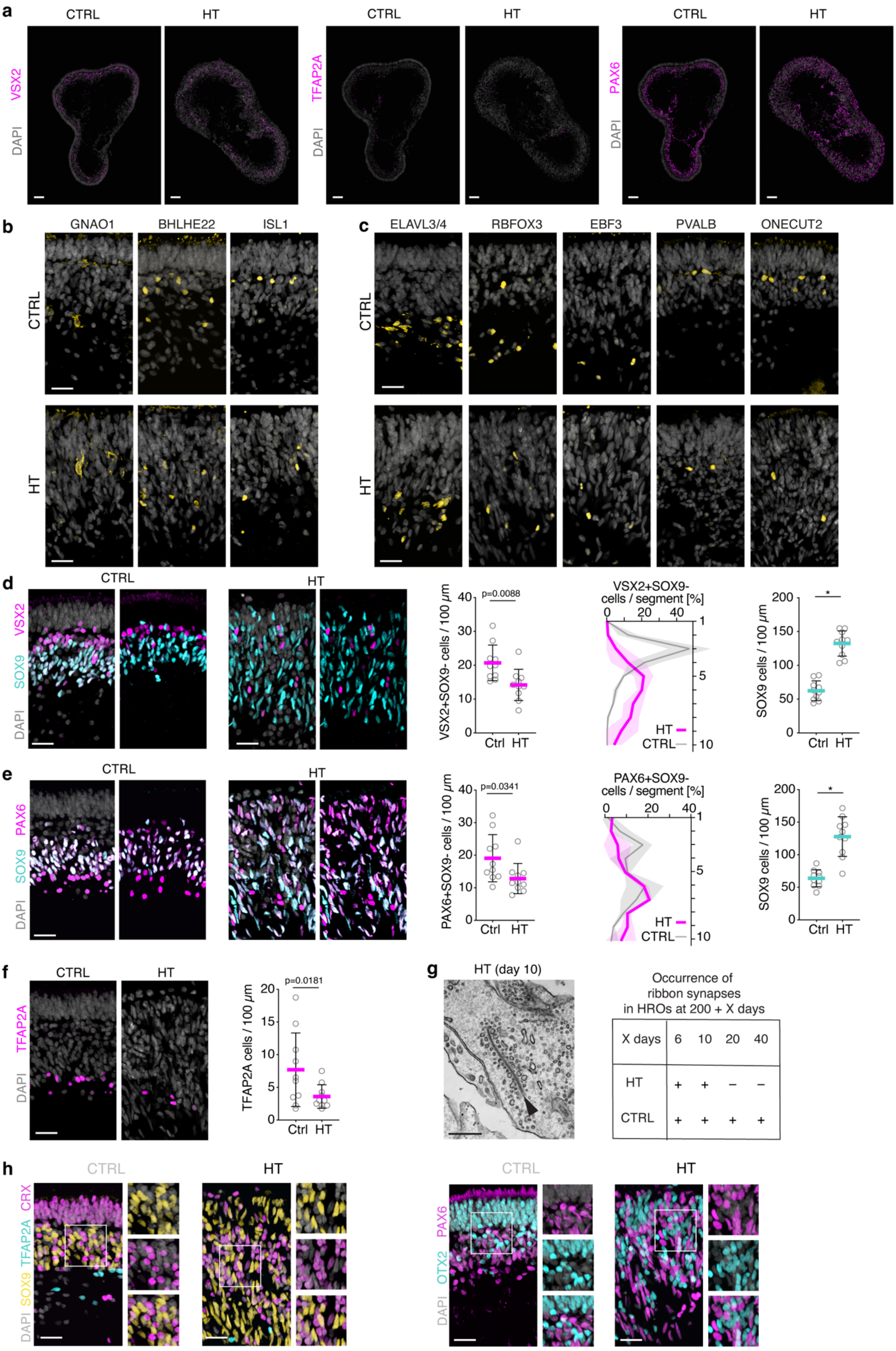

X days	6	10	20	40
HT	+	+	–	–
CTRL	+	+	+	+

Supplementary Figure 9: Pathologic changes of inner retinal neurons in HT-treated HROs.

(a-f, h) Immunostaining analysis of HRO sections. (a) Representative overview images of VSX2, TFAP2A, and PAX6 cell distribution in CTRL and HT-treated HROs. (b-c) Assessment of inner retinal neurons: several markers for (b) bipolar, as well as (c) amacrine, horizontal, and ganglion cells are expressed both in CTRL and HT-treated organoids. (a) For each marker: N=2 independent experiments (N) with n=5 HROs/N. (b, c, h) For each marker, N=1 independent experiments (N) with n=6 HROs/N. (d-f) Quantification of inner retinal neurons in CTRL and HT-treated HROs. Graphs: Each circle represents one individual HRO (n) based on N=2 independent experiments, n=5 HROs/N, mean of 4 ROIs/n. Graphs (mean ±SD) and statistics (two-sided Student's t-test, * $p<0.001$) over n. (d) Representative images and quantification of VSX2+SOX9− cells (bipolars). In CTRL, these are mostly located at the outer INL level. In HT-treated HROs, there are fewer VSX2+SOX9− cells, more widely distributed over the epithelium, though preferentially locating in the inner part. SOX9+ cells (Müller glia) were quantified in the same ROI for comparison. (e) Representative images and quantification of PAX6+SOX9− cells (amacrine, ganglion, and horizontal cells). In CTRL, PAX6+SOX9− cells peak in the outer INL part (presumably horizontal cells) and the inner epithelium (presumably amacrine and ganglion cells). In HT-treated HROs, there are fewer PAX6+SOX9− cells, localized more towards the inner epithelium. SOX9+ cells (Müller glia) were quantified in the same ROI for comparison. (f) Representative images and quantification of TFAP2A+ cells (amacrines). (g) Using TEM, ribbon synapses are detected in 200-days old HROs, and also after 10 days of HT treatment, but not thereafter. Table summarizes findings from 2 sample blocks from 1 HRO per indicated timepoint of CTRL and HT-treated HROs. (h) Validation of marker specificity: Müller glia (SOX9) do not express the photoreceptor-specific marker CRX in CTRL or HT-treated HROs. Further, OTX2 (photoreceptors and bipolars) and PAX6 (amacrine, horizontal, and ganglion cells) do not overlap in CTRL and HT-treated HROs. Insets show one enlarged region from the image on the left. Scale bars: (a) 100 µm, (b-f, h) 25 µm, (g) 500 nm. Source data are provided as a Source Data file.

Supplementary Figure 10

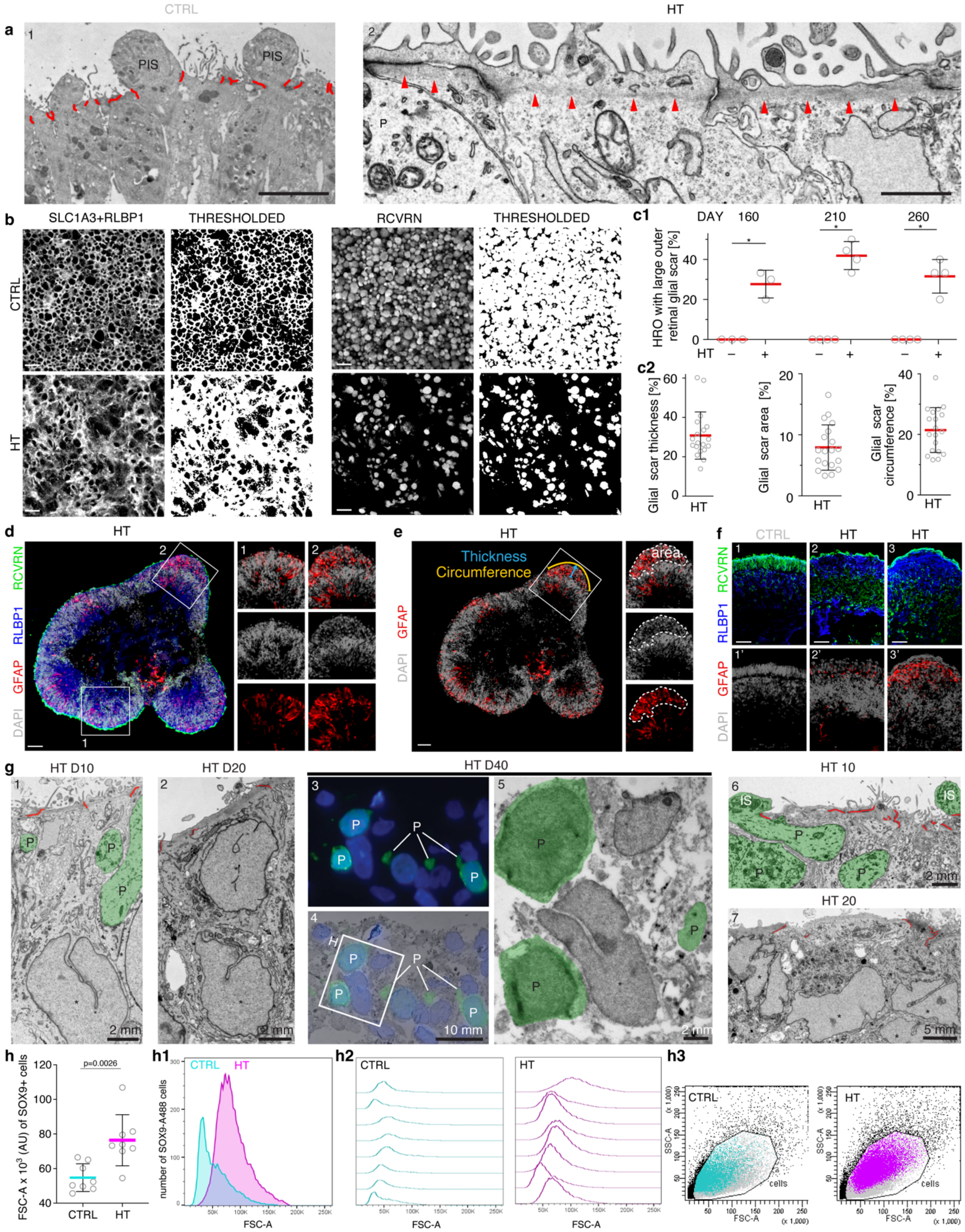

Supplementary Figure 10: Analyses of glial scar formation in HT-treated HROs. Data supplements Fig. 5. (a) Transmission electron microscopy (TEM) of epoxy-embedded HRO sections: in HT-treated (D200−210) HROs, large Müller glia (MG) processes extend along the apical surface: this is not observed in CTRL HROs. (1) Cell junctions are pseudocolored in red in CTRL. (2) MG processes show prominent electron-dense, filamentous structures spanning between cell junctions (red arrowheads). (b-f) MG scar formation analysis: analysis of CTRL and HT-treated HROs (D200−210, unless indicated otherwise): (b) en-face images of immunostained HRO wholemounts; and (c-f) immunostained HRO serial sections. (b) Images with defined stack sizes ranging from the photoreceptor segment to the outer limiting membrane (OLM) level were thresholded in Fiji and used to quantify (Fig. 5d) marker-positive areas: MG (SLC1A3 and RLBP1) and photoreceptors (RCVRN). N=1 independent experiments with n=9 HROs; 3 ROIs averaged/n. (c1) Count of HROs with a glial scar-like lesion. Graphs show mean ±SD, and each circle represents one independent experiments (N) with n≥5 HROs(n)/N. Two-sided Student's t-test over N (* $p<0.001$) (c2) Quantification of glial lesion area, circumference, and thickness, as depicted in (d-e) and normalized to total organoid area, circumference, and epithelial thickness, respectively. Graphs show mean±SD and each circle represents 1 individual HRO (n), data were averaged from 1 to 3 sections/n based on N=4 independent experiments with n≥3/N. (d-e) Overview and (f) two different regions of interests of an HRO section labeled for RLBP1 (MG), RCVRN (photoreceptors), and GFAP (upregulated in gliotic MG). In CTRL (f1,1’), photoreceptors are located in an apical, outer nuclear layer-like structure, no GFAP upregulation is seen, and MG processes are radially organized. In HT-treated HRO, a glial cell mass is dislocated apically above the photoreceptors (d2, f3, f3’), and GFAP upregulation (f3’) is stronger in some regions of the HRO, generating a glial scar-like lesion, but not as strongly in other regions (d1, f2, f2’). (e) Illustrated parameters quantified in (c). Glial lesions were defined as apical RCVRN− and GFAP+ areas. (g) (g1-2, g6-7) TEM of HRO sections: MG nuclei (asterisks) show an altered shape with nuclear segmentation after HT treatment. (g1,2) Epon sections, junctions of the OLM (red), and parts of photoreceptors (green) are pseudocolored. (g3-5) CLEM of a Tokuyasu-cryosection labelled with RCVRN (photoreceptors, green) and DAPI (blue). (g3) double fluorescence image, (g4) overlay of fluorescent and TEM images, (g5) region indicated by the square in (g4) at higher magnification, photoreceptors are highlighted in green. Cells with segmented nuclei do not express RCVRN, indicating that they are MG. (g6-7) Glial scarring after HT treatment for 10 (g6) and 20 days (g7). Photoreceptors (P) and their inner segments (PIS) are indicated (green), and the junctions of the OLM (red). N=1 independent experiments (N) with n=3 HROs (n) per timepoint and treatment. (h) Flow-cytometry analysis of the MG (SOX9) population showed an increase in the forward scatter (FSC), a proxy for cell size, in HT-treated HROs, suggesting glial

hypertrophy (graph shows mean ±SD and each circle represents one set (s) of pooled HRO, 2–3s/N, N=3, 2 hiPSC lines, ≥3935 SOX9+ cells/s; statistics (two-sided Student's t-test) over s; analysis based on data shown in Fig. 2e; Supplementary Fig. 6). (h1) Exemplary graph shows cell size shift of SOX9 cells from 2 individual representative sets of HROs. (h2) Graphs shows all individual HRO data that were used for SOX9 cell size quantification in (h). (h3) Example scatter plots from flow cytometry from cells of 2 individual sets of HROs. FSC, forward scatter area; SSC-A, side scatter area. Scale bars: (a1) 5 µm; (a2) 1 µm, (b) 10 µm, d-f) 100 µm. Source data are provided as a Source Data file.

Supplementary Figure 11

hiPSC lines: 5A CRTD1 CRTD2 IMR90

N=13, ≥5n/N, >65 HRO per variable, 4 hiPSC lines

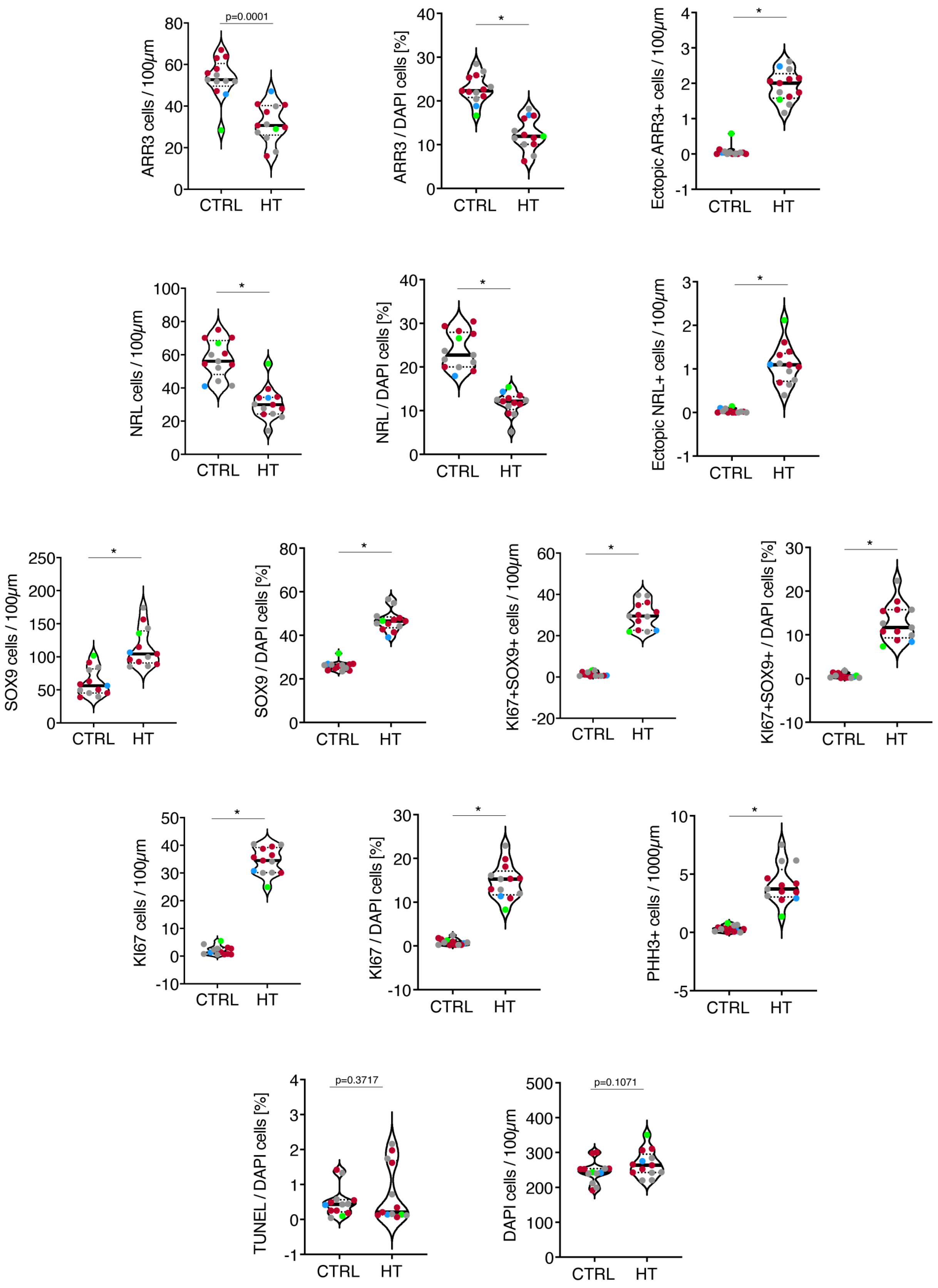

Supplementary Figure 11: Validation of the HT-HRO model in HROs derived from different hiPSC lines. The HRO system (Fig. 1) and HT-HRO model (Fig. 2; 3; 4 and related Supplementary Figures) were originally established using the 5A and CRTD1 hiPSC lines. Next, we reproduced selected findings by different methods (Fig. 2e; Supplementary Fig. 6) and in several different independent experimental set-ups using the 5A and CRTD1 hiPSC lines (dynamics: Fig. 6; Supplementary Fig. 12-14; pharmacologicals: Fig. 8-9; Supplementary Fig. 17-18), and 5A hiPSC line (single-cell RNA-seq: Fig. 1h-i; Supplementary Fig. 4, bulk RNA-seq: Fig. 7; Supplementary Fig. 15). In addition, here we validated the HRO system and HT-HRO model in another 2 hiPSC lines: the CRTD2 line was generated at our institute and had not previously been used for retinal differentiation, and the IMR90 line is commercially available and has been used by others for retinal organoid generation (Supplementary Fig. 1i). HROs at D200 were treated with HT for 10 days and compared to CTRL. Selected parameters were assessed on immunostained HRO sections: the total and ectopic number of cone (ARR3) and rod (NRL) photoreceptors were counted per region of interest (ROI). The total cell numbers were counted based on DAPI-labeled nuclei. A TUNEL assay was performed to assess cell death; labeled cells were counted. The number of total Müller glia (SOX9), and proliferating ones (KI67, PHH3), were counted. To depict a summary of the HRO data (all data at D210) of the four different cell lines, we calculated and plotted the mean for each independent experiment (N) (CRTD2, IMR90 presented only here in Fig. 9; CRTD1 and 5A data derived from Fig. 2c; 4; 6; 8-9). Graphs: Each dot represents the mean from ≥5 HROs (n) of one individual experiment (N), in total N=13, i.e. at least 65 HROs per variable (HT-treated vs. CTRL). Violin graphs show the median (thick line), and quartiles (dotted lines) over N. Experiments deriving from the different cell lines are shown in different colors as indicated in the figure. Two-sided Student's t-test over N; * $p<0.0001$. Source data are provided as a Source Data file.

Supplementary Figure 12

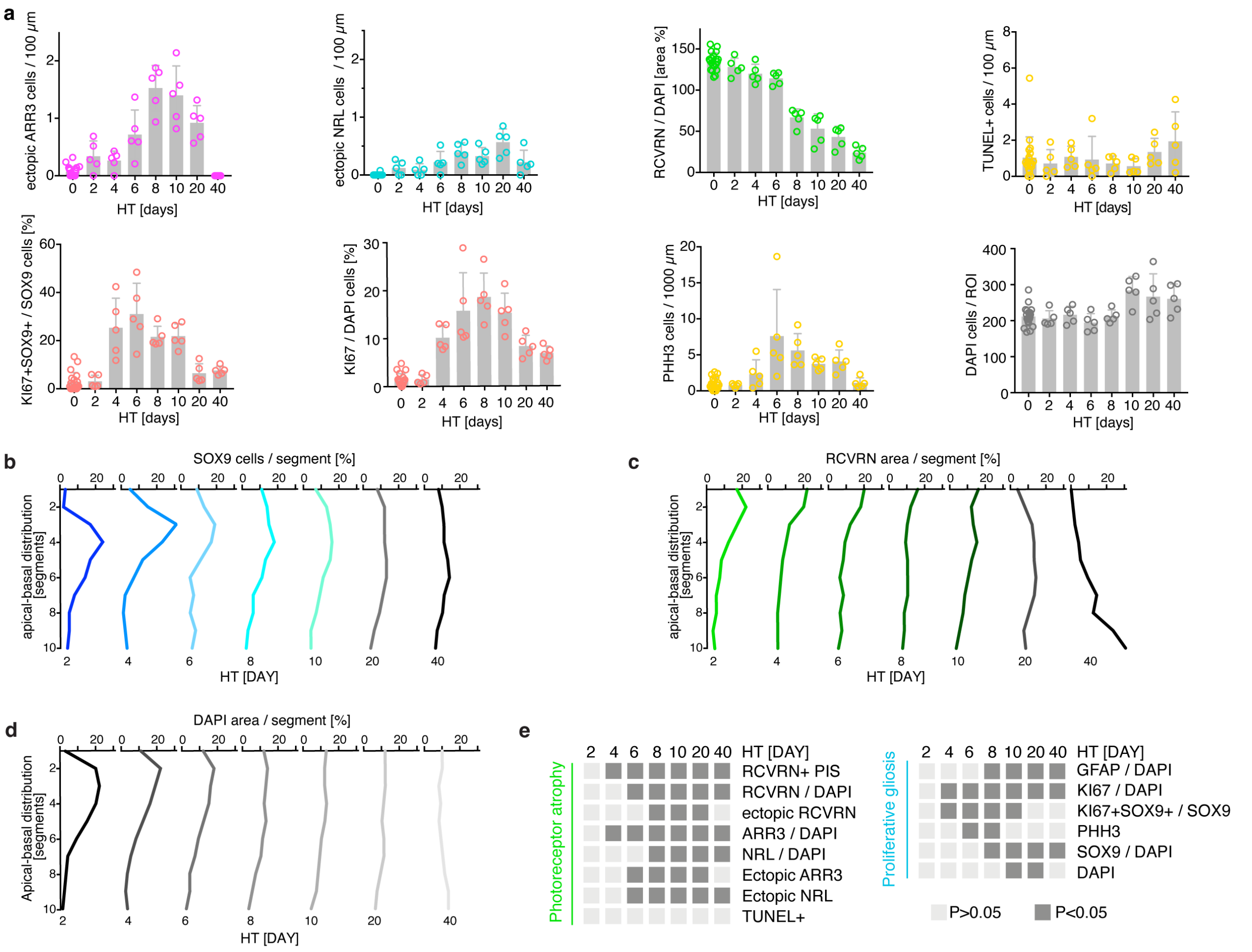

Supplementary Figure 12: Temporal dynamic analysis shows simultaneous development of HT-induced pathologies. Data supplements Fig. 6: HROs derived from the same batch of differentiation (original culture plate) received HT treatment: 50 ng/ml daily starting at D200. HT-treated HROs were collected and analyzed after 2, 4, 6, 8, 10, 20, and 40 days, and compared to day 0, which depicts all non-treatment controls taken on the respective day of HT treatment. (a) Quantitative analysis of HT-induced pathologies: cone (ARR3) and rod (NRL) photoreceptor cell ectopy; photoreceptor cell loss (RCVRN/DAPI); cell proliferation (KI67, PHH3); Müller glia (SOX9); total cell number (DAPI nuclear stain); and (b-d) retinal dyslamination (using Müller glia (SOX9), photoreceptor (RCVRN), and DAPI delamination as a proxy). (a) Graphs: Each circle represents 1 individual HRO (n) based on N=1 independent experiment. Graphs (mean ±SD) and (e) statistics over n. (b-d) Lines represent mean distribution derived from individual HROs (n); n=5 HROs per independent experiment (N); N=1; 2 regions of interest per HRO. (e) Graphical depiction of statistical analyses for data in (a) and Fig. 6. Indicated timepoints compared to control (D0) based on one-way ANOVA (Tukey's post-hoc test). Photoreceptor inner segments (PIS). Source data are provided as a Source Data file.

Supplementary Figure 13

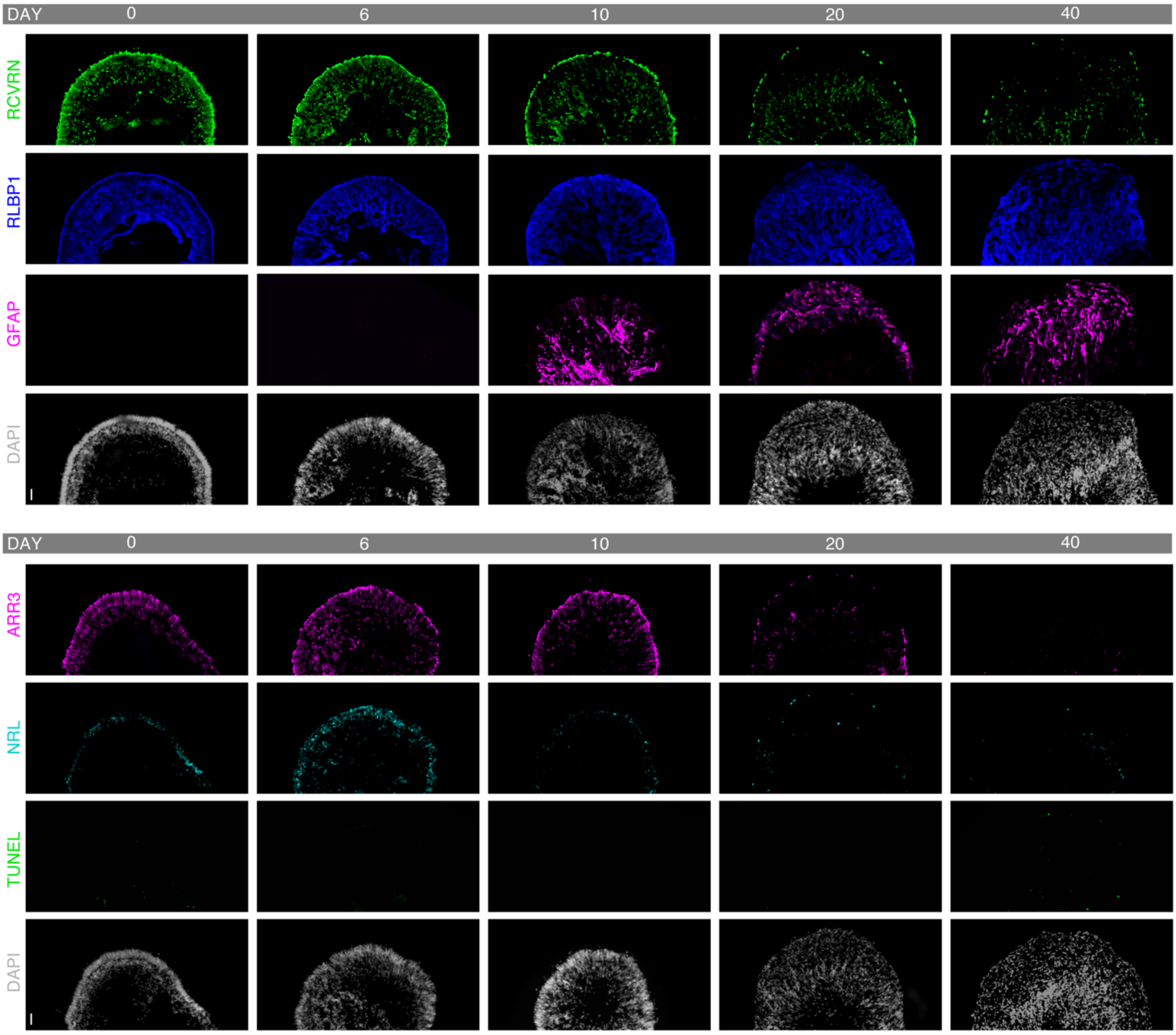

Supplementary Figure 13: Overview images for temporal progression of the HT-HRO model. Data supplements Fig. 6; Supplementary Fig. 12. HROs were treated with HT for up to 40 days and collected at the timepoints indicated. Representative images showing about half of a central HRO section immunostained for photoreceptor (RCVRN), Müller glia (RLBP1), gliosis (GFAP), and cell nuclei (DAPI) markers, as well as for cone (ARR3) and rod (NRL) photoreceptor markers, and TUNEL assay (cell death). N=1 independent experiments (N) with n≥5 HROs per timepoint. Scale bar: 50 µm.

Supplementary Figure 14

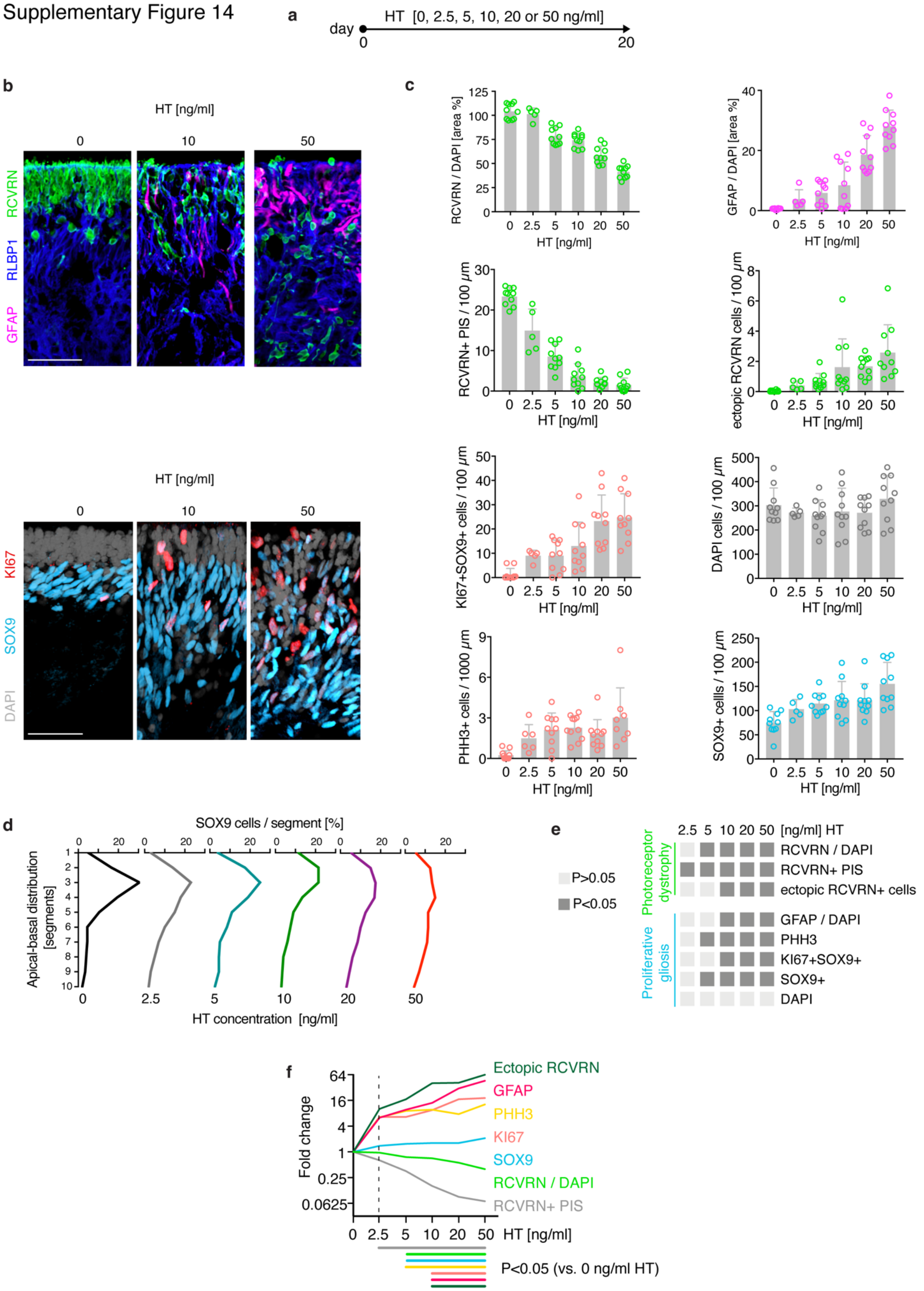

Supplementary Figure 14: Dose-dependency of HT-induced pathologies in HROs. (a) Experimental design: HROs derived from the same batch of differentiation (original culture plate) were divided per experiment into control (without HT, 0 ng/ml) and treatment culture plates: HT was applied daily from D200 to 220 at different concentrations (2.5, 5, 10, 20, and 50 ng/ml). (b) Representative images of immunostained tissue sections from CTRL and HT-treated HROs; representative images shown for selected HT concentrations. Immunostainings shown for RCVRN (photoreceptors), SOX9 and RLBP1 (Müller glia), GFAP (upregulated in gliotic Müller glia), cell proliferation marker (KI67), and cell nuclei (DAPI). N=2 independent experiments (N) with n≥5 HRO (n) per variable. (c) Quantitative data of HT-induced pathologies (based on cell markers analyzed): photoreceptor cell loss and ectopy (RCVRN), photoreceptor inner segment (PIS) loss (RCVRN+ PIS); gliosis (GFAP); cell proliferation (KI67, PHH3); Müller glia (SOX9); total cell number (DAPI nuclear stain); (d) retinal dyslamination: Müller glia delamination (SOX9). Graphs: Each circle represents 1 individual HRO (n) based on N=2 independent experiments. Graphs (mean ±SD) and (e) statistics over n. (d) Lines represent mean distribution derived from individual HROs (n); n=5 per independent experiment (N); N=2 (2.5 ng/ml HT N=1), 2 ROIs/n. (e) Graphical depiction of statistical analyses for data in (c). Indicated HT doses compared to CTRL (0 ng/ml) based on 1-way ANOVA (Tukey's post-hoc test). (f) Summary of quantitative data shown in (c). Scale bar: (b) 50 µm. Source data are provided as a Source Data file.

Supplementary Figure 15

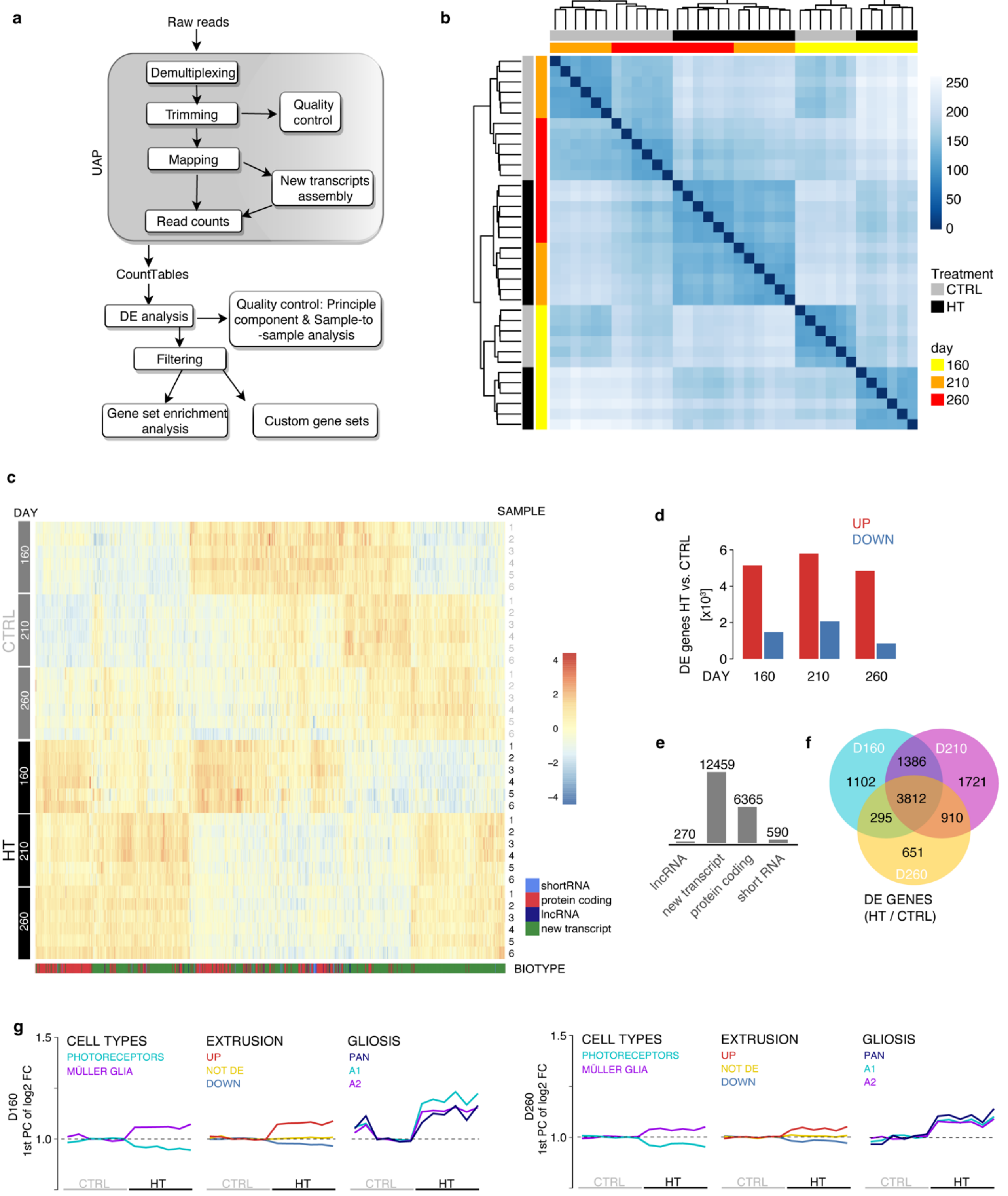

Supplementary Figure 15: Transcriptomics data analyses of the HT-HRO model. Data supplements Fig. 7. (a) RNA-seq data analysis workflow. (b) Heatmap of the sample-to-sample distance based on rlog-transformed counts of all HRO samples (36 samples with 6 for each timepoint and treatment: D160, 210, and 260, each with HT treatment (10 days) and without (CTRL). (c) Heatmap of the 21149 differentially-expressed genes (FDR 0.01; total number of expressed genes: 56642) based on rlog-transformed counts of the 36 samples shown in (b) (see row annotation). The genes are tagged with their biotype (see column annotation). (d) Numbers of differentially-expressed (DE) genes (DEG): number of genes down- and up-regulated between pairwise comparison of HT-treated and CTRL samples for each timepoint. (e) Distribution of the biotypes across the DEG. The biotypes were annotated using biomart R package[32] and the gencode annotation (release Mai 2019). (f) Numbers of DEG that overlap between the indicated timepoints (D, day). (g) Related to Fig. 7d: Graphs depicting the first principal component of the log2 fold change (log2FC) in CTRL and HT-treated HROs at D160 and 260 for custom-made genes of interest (GOI) lists based on published data, representing retinal cell types (photoreceptors and Müller glia cells), cell extrusion regulators, and reactive gliosis (marker for pan-gliosis (PAN), A1, and A2 gliosis states). Data have been deposited on Gene Expression Omnibus.

Supplementary Figure 16

HT-HRO model related process	EGSEA terms based on HT-HRO compared to control	EGSEA term ID
HT signaling related	Cytokine-cytokine receptor interaction	hsa04060
	TNF signaling pathway	hsa04668
	EGFR_UP.V1_UP	M2634
	ERB2_UP.V1_UP	M2636
	MAPK signaling pathway	M10792
	HALLMARK_INFLAMMATORY_RESPONSE	M5932
	HALLMARK_COMPLEMENT	M5921
	GO_IMMUNE_RESPONSE	M19817
Photoreceptor pathology	HALLMARK_REACTIVE_OXIGEN_SPECIES_PATHWAY	M5938
	HALLMARK_UNFOLDED_PROTEIN_RESPONSE	M5922
	cGMP-PKG signaling pathway	hsa04022
	GO_BP_visual_perception	GO:0007601
	HALLMARK_MTORC1_SIGNALING	M5924
	HIF-1 signaling pathway	hsa04066
	MORF_HDAC1	M11080
Glia pathology	GO_BP_cell_proliferation	GO:0008283
	Notch signaling pathway	M7946
	HALLMARK_TGF_BETA_SIGNALING	M5896
	Reactome_Mitotic_M-M/G1_phases	M7634
	CYCLIN_D1_UP.V1_DN	M2652
Photoreceptor extrusion, scar formation & retinal remodeling	Tight junction	M11355
	Adherens junction	M638
	Regulation of actin cytoskeleton	M18306
	GO_BP_regulation_of_cell_shape	GO:0008360
	GO_BP_cell_migration	GO:0016477
	Sphingolipid signaling pathway	M15955
	HALLMARK_EPITHELIAL_MESENCHYMAL_TRANSITION	M5930
Inflammation, cell death, biomechanical cell stress and others	GO_REGULATION_OF_RESPONSE_TO_STRESS	M13345
	HALLMARK_DNA_REPAIR	M5898
	Apoptosis	M5902
	CORDENONSI_YAP_CONSERVED_SIGNATURE	M2871
	Hippo signaling pathway	mmu04390
	HALLMARK_GLYCOLYSIS	M5937
	REACTOME_AMYLOIDS	M1076

Supplementary Figure 16: Selected terms from the EGSEA of the HT-HRO model. An Ensemble of Gene Set Enrichment Analysis (EGSEA) for the differentially-expressed genes (DEGs) between HT-treated and control HRO yielded >1000 enriched terms (Supplementary Data 5a). We selected several sample terms that support or are potentially relevant for the pathologic processes determined in HT-HRO by histological analysis.

Supplementary Figure 17

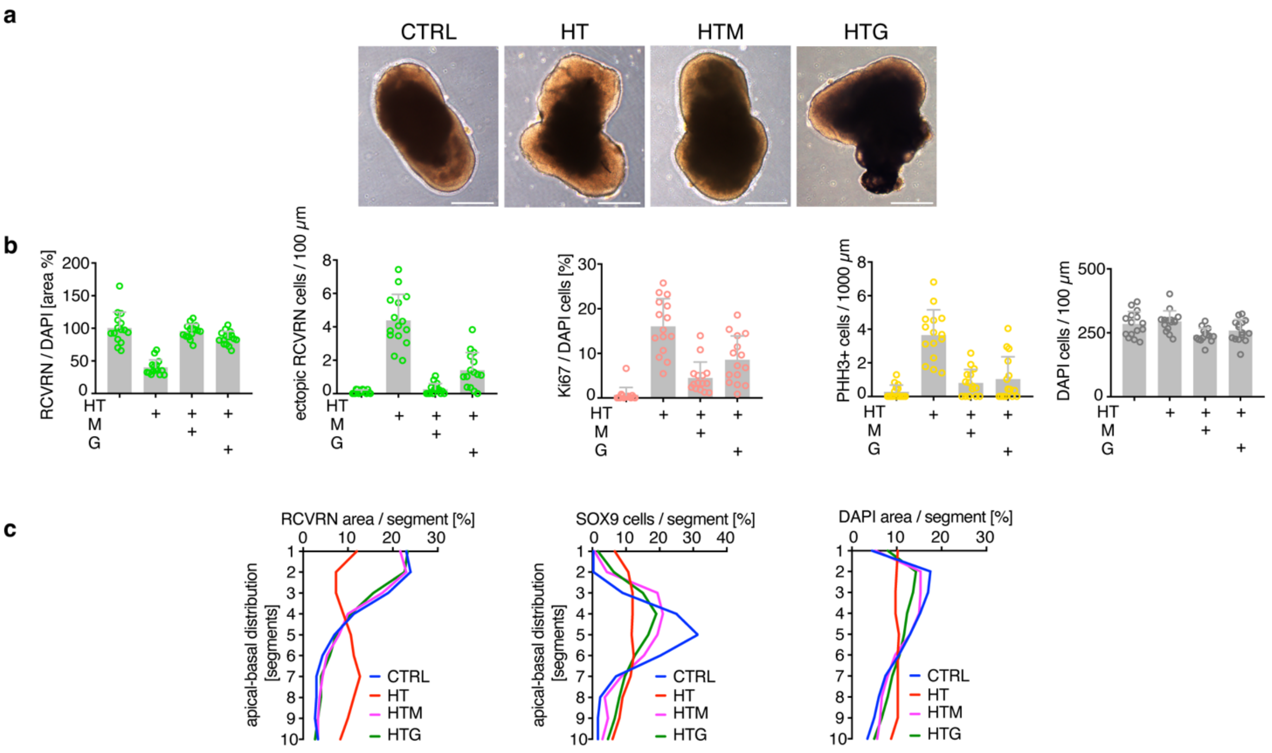

Supplementary Figure 17: MEKi or GsMTx4 treatment each inhibits photoreceptor and glial pathologies, as well as retinal dyslamination in the HT-HRO model. Data supplements Fig. 8 and Supplementary Data 6. Experimental design shown in Fig. 8a. (a) Representative phase contrast images of CTRL, and HT-, HTM (HT+MEKi) and HTG- (HT+GsMTx4) treated HROs. N=3 independent experiments (N) with n≥5 HROs/N for each variable. (b) Quantitative analysis to determine the effect of the pharmacological inhibitors on the HT-HRO phenotype: pathologic changes of photoreceptors (RCVRN), photoreceptor cell ectopy, cell proliferation (KI67), and mitosis (PHH3), as well as DAPI. Graphs: Each circle represents 1 individual HRO (n) based on N=3 independent experiments (n≥5/N). Graphs (mean ±SD) over n. (c) Structural retina dyslamination was assessed by quantifying radial cell delamination of photoreceptors (RCVRN), Müller glia nuclei (SOX9), and total cells (DAPI). Lines represent mean distribution derived from individual HROs (n) derived from N=3 independent experiments (n≥5/N). Scale bars: (a) 500 µm. Source data are provided as a Source Data file.

Supplementary Figure 18

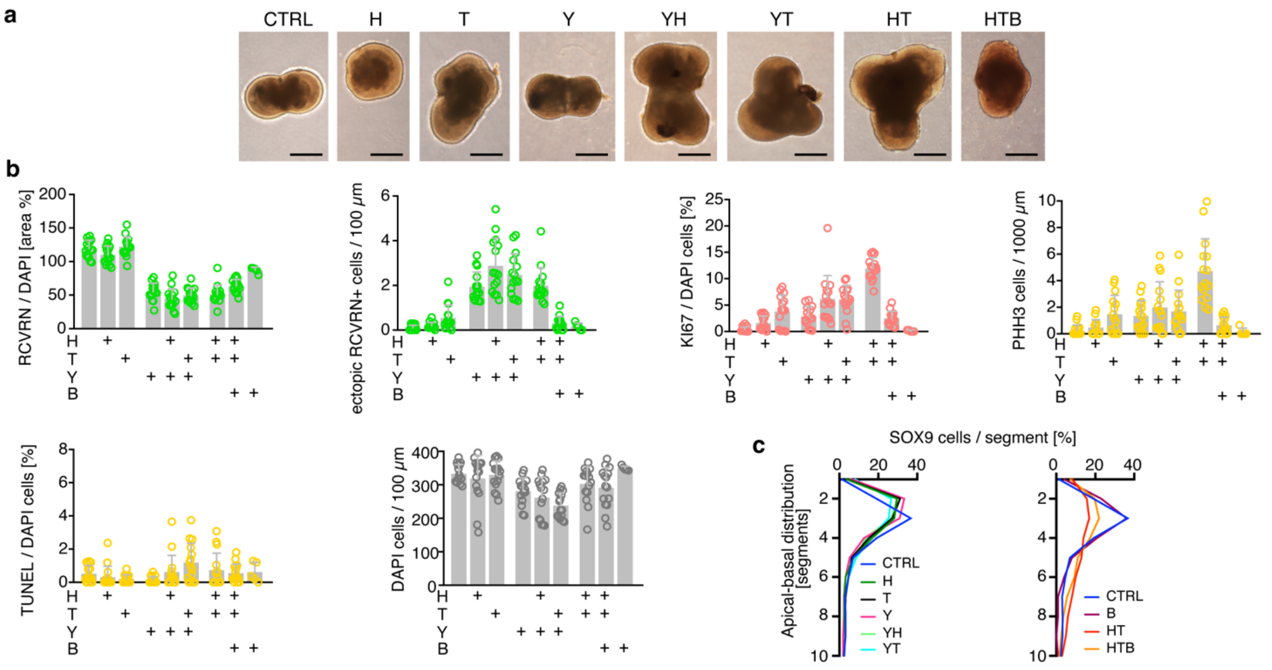

Supplementary Figure 18: Effect of PIEZO1 activator YODA1, Blebbistatin, and single versus combined HBEGF and TNF in HROs. Data supplements Fig. 9 and Supplementary Data 6. Experimental design shown in Fig. 9a. (a) Representative phase contrast images of TNF (T), HBEGF (H), HT, YODA1 (Y), YT, YH and HTB (HT with (-)-Blebbistatin (B)) treated HROs and controls. N=3 independent experiments (N) with n≥5 HROs/N for each variable. (b) Quantitative analysis to determine the effect of the different treatment paradigms on the HROs: pathologic changes of photoreceptors (RCVRN), photoreceptor cell ectopy, cell proliferation (KI67), mitosis (PHH3), cell death (TUNEL), and DAPI. Graphs: Each circle represents 1 individual HRO (n) based on N=3 independent experiments (n≥5/N). Graphs (mean ±SD) over n. (c) Structural retina dyslamination was assessed by quantification of radial cell delamination of Müller glia nuclei (SOX9). Lines represent mean distribution derived from individual HROs (n) derived from N=3 independent experiments (n≥5/N). Scale bars: (a) 500 µm. Source data are provided as a Source Data file.

References

1. Yi W*, et al.* A single-cell transcriptome atlas of the aging human and macaque retina. *National Science Review* **8**, (2020).

2. Hendrickson AE, Yuodelis C. The morphological development of the human fovea. *Ophthalmology* **91**, 603-612 (1984).

3. Wahlin KJ*, et al.* Photoreceptor Outer Segment-like Structures in Long-Term 3D Retinas from Human Pluripotent Stem Cells. *Sci Rep* **7**, 766 (2017).

4. Wagner F, Haase R. felwag. (2022). felwag/photoreceptor-pattern-analysis: Photoreceptor pattern analysis (v1.0.0). Zenodo. https://doi.org/10.5281/zenodo.7040860.). GitHub repository (2022).

5. Polyak SL. *The retina*. Univ. Chicago Press (1941).

6. Curcio CA, Sloan KR, Kalina RE, Hendrickson AE. Human photoreceptor topography. *J Comp Neurol* **292**, 497-523 (1990).

7. Lombardo M, Serrao S, Ducoli P, Lombardo G. Eccentricity dependent changes of density, spacing and packing arrangement of parafoveal cones. *Ophthalmic Physiol Opt* **33**, 516-526 (2013).

8. Sawides L, de Castro A, Burns SA. The organization of the cone photoreceptor mosaic measured in the living human retina. *Vision Res* **132**, 34-44 (2017).

9. Curcio CA, Sloan KR, Jr., Packer O, Hendrickson AE, Kalina RE. Distribution of cones in human and monkey retina: individual variability and radial asymmetry. *Science* **236**, 579-582 (1987).

10. Voigt AP*, et al.* Molecular characterization of foveal versus peripheral human retina by single-cell RNA sequencing. *Exp Eye Res* **184**, 234-242 (2019).

11. Bringmann A*, et al.* The primate fovea: Structure, function and development. *Prog Retin Eye Res*, (2018).

12. Rattner A, Nathans J. Macular degeneration: recent advances and therapeutic opportunities. *Nat Rev Neurosci* **7**, 860-872 (2006).

13. Sarks JP, Sarks SH, Killingsworth MC. Evolution of geographic atrophy of the retinal pigment epithelium. *Eye (Lond)* **2 (Pt 5)**, 552-577 (1988).

14. Curcio CA. Photoreceptor topography in ageing and age-related maculopathy. *Eye (Lond)* **15**, 376-383 (2001).

15. Fleckenstein M*, et al.* Age-related macular degeneration. *Nat Rev Dis Primers* **7**, 31 (2021).

16. Jeon CJ, Strettoi E, Masland RH. The major cell populations of the mouse retina. *J Neurosci* **18**, 8936-8946 (1998).

17. Syrbe S*, et al.* Muller glial cells of the primate foveola: An electron microscopical study. *Exp Eye Res* **167**, 110-117 (2018).

18. Jonas JB, Schneider U, Naumann GO. Count and density of human retinal photoreceptors. *Graefes Arch Clin Exp Ophthalmol* **230**, 505-510 (1992).

19. Reichenbach A, Bringmann A. Glia of the human retina. *Glia*, (2019).

20. Li M*, et al.* Histology of gegraphic atrophy secondary to age-related macular degeneration: A multilayer apporach. *Retina*, (2018).

21. Hendrickson A. Organization of the Adult Primate Fovea. In: *Macular Degeneration* (eds Penfold PL, Provis JM). Springer Berlin Heidelberg (2005).

22. Song H, Chui TY, Zhong Z, Elsner AE, Burns SA. Variation of cone photoreceptor packing density with retinal eccentricity and age. *Invest Ophthalmol Vis Sci* **52**, 7376-7384 (2011).

23. Provis JM, Penfold PL, Cornish EE, Sandercoe TM, Madigan MC. Anatomy and development of the macula: specialisation and the vulnerability to macular degeneration. *Clin Exp Optom* **88**, 269-281 (2005).

24. Cooper RF, Wilk MA, Tarima S, Carroll J. Evaluating Descriptive Metrics of the Human Cone Mosaic. *Invest Ophthalmol Vis Sci* **57**, 2992-3001 (2016).

25. Curcio CA, Sloan KR. Packing geometry of human cone photoreceptors: variation with eccentricity and evidence for local anisotropy. *Vis Neurosci* **9**, 169-180 (1992).

26. Hirsch J, Curcio CA. The spatial resolution capacity of human foveal retina. *Vision Res* **29**, 1095-1101 (1989).

27. Wells-Gray EM, Choi SS, Bries A, Doble N. Variation in rod and cone density from the fovea to the mid-periphery in healthy human retinas using adaptive optics scanning laser ophthalmoscopy. *Eye (Lond)* **30**, 1135-1143 (2016).

28. Ziesel A, Bernstein S, Wong PW. Generation of a foveomacular transcriptome. *Mol Vis* **20**, 947-954 (2014).

29. Bowes Rickman C*, et al.* Defining the human macula transcriptome and candidate retinal disease genes using EyeSAGE. *Invest Ophthalmol Vis Sci* **47**, 2305-2316 (2006).

30. Hoshino A*, et al.* Molecular Anatomy of the Developing Human Retina. *Dev Cell* **43**, 763-779 e764 (2017).

31. Wang J*, et al.* ATAC-Seq analysis reveals a widespread decrease of chromatin accessibility in age-related macular degeneration. *Nature communications* **9**, 1364 (2018).

32. Durinck S, Spellman PT, Birney E, Huber W. Mapping identifiers for the integration of genomic datasets with the R/Bioconductor package biomaRt. *Nat Protoc* **4**, 1184-1191 (2009).

33. Wagner BD*, et al.* Association of Systemic Inflammatory Factors with Progression to Advanced Age-related Macular Degeneration. *Ophthalmic Epidemiol*, 1-10 (2021).

34. Lynch AM*, et al.* Plasma Biomarkers of Reticular Pseudodrusen and the Risk of Progression to Advanced Age-Related Macular Degeneration. *Transl Vis Sci Technol* **9**, 12 (2020).

35. Luthert PJ, Kiel C. Combining Gene-Disease Associations with Single-Cell Gene Expression Data Provides Anatomy-Specific Subnetworks in Age-Related Macular Degeneration. *Netw Syst Med* **3**, 105-121 (2020).

36. Logue MW*, et al.* Search for age-related macular degeneration risk variants in Alzheimer disease genes and pathways. *Neurobiol Aging* **35**, 1510 e1517-1518 (2014).

37. Klein R*, et al.* Markers of inflammation, oxidative stress, and endothelial dysfunction and the 20-year cumulative incidence of early age-related macular degeneration: the Beaver Dam Eye Study. *JAMA Ophthalmol* **132**, 446-455 (2014).

38. Han X, Gharahkhani P, Mitchell P, Liew G, Hewitt AW, MacGregor S. Genome-wide meta-analysis identifies novel loci associated with age-related macular degeneration. *J Hum Genet* **65**, 657-665 (2020).

39. Fritsche LG*, et al.* A large genome-wide association study of age-related macular degeneration highlights contributions of rare and common variants. *Nature genetics* **48**, 134-143 (2016).

40. Ratnapriya R*, et al.* Retinal transcriptome and eQTL analyses identify genes associated with age-related macular degeneration. *Nature genetics* **51**, 606-610 (2019).

41. Kim EJ, Grant GR, Bowman AS, Haider N, Gudiseva HV, Chavali VRM. Complete Transcriptome Profiling of Normal and Age-Related Macular Degeneration Eye Tissues Reveals Dysregulation of Anti-Sense Transcription. *Sci Rep* **8**, 3040 (2018).

42. Newman AM*, et al.* Systems-level analysis of age-related macular degeneration reveals global biomarkers and phenotype-specific functional networks. *Genome Med* **4**, 16 (2012).

43. Saddala MS, Lennikov A, Mukwaya A, Fan L, Hu Z, Huang H. Transcriptome-wide analysis of differentially expressed chemokine receptors, SNPs, and SSRs in the age-related macular degeneration. *Hum Genomics* **13**, 15 (2019).

44. Lyu Y*, et al.* Implication of specific retinal cell-type involvement and gene expression changes in AMD progression using integrative analysis of single-cell and bulk RNA-seq profiling. *Sci Rep* **11**, 15612 (2021).

45. Clarke LE, Liddelow SA, Chakraborty C, Munch AE, Heiman M, Barres BA. Normal aging induces A1-like astrocyte reactivity. *Proc Natl Acad Sci U S A* **115**, E1896-E1905 (2018).

46. Whitmore SS*, et al.* Altered gene expression in dry age-related macular degeneration suggests early loss of choroidal endothelial cells. *Mol Vis* **19**, 2274-2297 (2013).
