# Supplementary material for: HBEGF-TNF induce a complex outer retinal pathology with photoreceptor cell extrusion in human organoids
Source: Nat Commun. 2022 Oct 19;13:6183. doi: 10.1038/s41467-022-33848-y (PMC9581928; doi:10.1038/s41467-022-33848-y)
Supplement: Supplementary file 2 — Description of Additional Supplementary Files [file 41467_2022_33848_MOESM2_ESM.pdf]

Description of Additional Supplementary Files

File name: Supplementary Data 1

Description: References potentially supporting an association between the HT-HRO model and AMD.

A curated list of selected references with a brief summary of the findings potentially relevant for the HT-HRO model, potential data limitations, and the location of the related data, including GWAS and other studies predicting EGF- or TNF-signaling related candidates as potential AMD risk genes or AMD progression factors; and analysis of EGF- or TNF-signaling related genes and proteins in AMD patients. All references cited in the file: 33-44.

File name: Supplementary Data 2

Description: Quantitative data of the HBEGF-TNF-induced pathology model.

Summary of quantitative data partly shown in Fig. 2; 4; Supplementary Fig. 5; 7-8. Separate sets of human retinal organoids (HROs) were treated with HT from D150 (a), 200 (b), or 250 (c), and analyzed after 10 days of treatment. Details on analyzed numbers of (n) individual organoids derived from (N) independent experiments and (L) hiPSC lines, as well as p-values (Students t-test) are provided. Abbreviations: human retinal organoid (HRO), day (D), control organoid (CTRL), HBEGF-TNF-treated organoid (HT), region of interest (ROI, see Methods), outer limiting membrane (OLM), Müller glia (MG), photoreceptor (PR), individual organoids analyzed (n), independent experiments (N), human induced pluripotent stem cell (hiPSC) lines (L).

File name: Supplementary Data 3

Description: Data summary for live-imaging cell extrusion studies.

Related to Fig. 3a. List of movies recorded by live-imaging microscopy. Legend: sample name; HT day: day of recording after start of HBEGF-TNF (HT) treatment; and #ROI: number of regions of interest (ROI) recorded per sample. CTRL: control (not HT treated). HROs were derived from the 5A hiPSC line. The Supplementary Movies 2-4 are recorded from HRO5.

File name: Supplementary Data 4

Description: Custom-made genes of interest (GOI) lists.

Custom-made GOI lists were established from published data for cell-type-specific genes expressed in photoreceptors (PR) and Müller glia (MG); reactive gliosis phenotypes45: proinflammatory, neurotoxic (A1) glia, neuroprotective, anti-inflammatory (A2) glia, and pan-reactive glial marker (PAN); and cell extrusion. GOI lists were applied in Fig. 7d-e and Supplementary Fig. 15g as indicated. The table depicts the differentially-

expressed (DE) genes, indicated by 1 (zero are not DE) in the HT-HRO model (comparing HT vs. CTRL samples from D210).

File name: Supplementary Data 5

Description: Overview of AMD retina datasets and AMD risk genes used for explorative analysis.

Sheet 5a: EGSEA HT-HRO & CTRL-HRO: Overview of enriched gene set as a result of an EGSEA of genes that are differentially expressed between HT and CTRL samples taken at D160, D210, and D260. The columns are: EGSEA terms – the name of the gene set, D[160,210,260] – the row-wise (per gene set) normalized adjusted p-values of DEGs of the respective comparison, Source – the source of the gene set (we used gene sets from gene set DB, KEGG, and GO terms), and Cluster – the associated cluster of this gene set according to a hierarchical clustering of the enriched terms. Sheet 5b: GOI AMD risk genes: The genes-of-interest (GOI) list is based on a previous genome-wide association study of AMD39. Sheet 5c: AMD patient retina datasets: Overview table for the different datasets that we explored for selected differentially-expressed genes (DEGs) in the HT-HRO model. In this table clinical AMD phenotype, AMD stage, number of patients included in the study, total number of DEGs in AMD patients compared to healthy individuals, analysis method, tissue-sample size and type, as well as statistics applied in the respective studies are collected from the indicated references31, 40-44, 46. We provide the original data location and acronyms in the column termed original dataset location / dataset details in the reference. FDR, false discovery rate.

File name: Supplementary Data 6

Description: Quantitative data for pharmacological studies of the HBEGF-TNF-induced pathology model.

Summary of quantitative data shown in Fig. 8-9; Supplementary Fig. 17-18. Details on p-values (ANOVA) are provided. Abbreviations: control organoid (CTRL), HBEGF-TNF-treated (HT), MEK inhibitor (MEKi), Blebbistatin (BLEB), region of interest (ROI), not significant (n.s.).

File name: Supplementary Data 7

Description: List of antibodies and in-situ assays.

Abbreviations: Catalogue number (Cat.no), correlative light and electron microscopy (CLEM), Müller glia (MG), amacrine cell (AC), horizontal cell (HC), ganglion cell (GC).

File name: Supplementary Movie 1

Description: En-face imaging analysis of HROs for cone photoreceptor markers.

Confocal microscopy optical image stack of a fixed D229 HRO wholemount immunostained for the pan-cone marker ARR3 in combination with the cone subtype markers OPN1LW/MW and OPN1SW (image slice distance: 0.6 µm; image stack size: 16.2 µm; number of image slices: 27). Photoreceptor outer segments are observed first when approaching the HRO from the apical site, followed by photoreceptor inner segments, and photoreceptor cell soma in the outer nuclear layer. Note that OPN1LW/MW+ cells are frequently observed, while only few cells are OPN1SW+ (arrowhead). Arrows indicate cells where outer and inner segments, as well as nuclei, can be tracked for individual photoreceptors.

File name: Supplementary Movie 2

Description: Live-imaging analyses of cell extrusion dynamics in HT-HROs.

Differential interference contrast (DIC) and fluorescence spinning disk imaging microscopy of a whole living human retinal organoid (HRO) in culture on D6 of HT-treatment. The nuclear live-dye SiR-DNA was added to HROs acutely prior to imaging. DIC imaging reveals position of the outer limiting membrane (OLM) and photoreceptor inner segments, the first image snapshot shows the region of interest (ROI) where the subsequently presented movie was recorded. For this movie, images were recorded at a frequency of 4 frames per hour (N=2). The SiR-DNA-labeled cell nucleus (red) is extruded within 90min of imaging. The slow-motion image series shows a change in cell nuclear shape (hourglass-like) as if it were being squeezed through the outer organoid border, possibly the outer limiting membrane (OLM) as also observed by electron microscopy (Fig.3 e6, e7).

File name: Supplementary Movie 3

Description: Live-imaging analyses of cell extrusion dynamics in HT-HROs.

Another recoding of cell extrusion comparable to Supplemental Movie 2. Differential interference contrast (DIC) and fluorescence spinning disk imaging microscopy of a whole living human retinal organoid (HRO) in culture on D6 of HT-treatment. The nuclear live-dye SiR-DNA was added to HROs acutely prior to imaging. DIC imaging reveals position of the outer limiting membrane (OLM) and photoreceptor inner segments, the first image snapshot shows the region of interest (ROI) where the subsequently presented movie was recorded. For this movie, images were recorded at a frequency of 4 frames per hour (N=2). The SiR-DNA-labeled cell nucleus (red) is extruded within 90min of imaging. The slow-motion image series shows a change in cell nuclear shape (hourglass-like) as if it were being squeezed through the outer organoid border, possibly the outer limiting membrane (OLM) as also observed by electron microscopy (Fig.3 e6, e7).

File name: Supplementary Movie 4

Description: Sodium-fluorescein-assisted ectopic cell visualization in HT-HRO model.

Living whole human retinal organoid (HRO) in cell culture treated with HT for 9 days or without were acutely stained with the nuclear live-dye SiR-DNA. Further, to visualize the external space surrounding the HROs sodium-fluorescein was added to the cell culture medium. Using fluorescence spinning disk confocal microscopy, HROs were imaged: SiR-DNA revealed cell nuclei of whole HROs which is clearly distinguishable from the surrounding fluorescein signal. In controls, but not in HT-treated HROs, a fluorescein-negative band surrounding the HRO can be seen at the intersection of fluorescein and SiR-DNA labeled nuclei potentially representing protruding photoreceptor inner and outer segments. Based on this data, the apical HRO border and position of the outer limiting membrane (OLM) was approximated, as indicated by a yellow line. In HT-HROs, but not in controls, numerous brightly SiR-DNA-labeled cell nuclei can be identified above the yellow line and clearly positioned within (surrounded by) fluorescein indicative of cells outside (ectopic) of the HRO.
