# Supplementary material for: HBEGF-TNF induce a complex outer retinal pathology with photoreceptor cell extrusion in human organoids
Source: Nat Commun. 2022 Oct 19;13:6183. doi: 10.1038/s41467-022-33848-y (PMC9581928; doi:10.1038/s41467-022-33848-y)
Supplement: Supplementary file 14 — Reporting Summary [file 41467_2022_33848_MOESM14_ESM.pdf]

nature portfolio

Corresponding author(s): Mike Karl

Last updated by author(s): Sep 12, 2022

Reporting Summary

Nature Portfolio wishes to improve the reproducibility of the work that we publish. This form provides structure for consistency and transparency in reporting. For further information on Nature Portfolio policies, see our Editorial Policies and the Editorial Policy Checklist.

Statistics

For all statistical analyses, confirm that the following items are present in the figure legend, table legend, main text, or Methods section.

n/a	Confirmed	
☐	☒	The exact sample size (*n*) for each experimental group/condition, given as a discrete number and unit of measurement
☐	☒	A statement on whether measurements were taken from distinct samples or whether the same sample was measured repeatedly
☐	☒	The statistical test(s) used AND whether they are one- or two-sided<br>*Only common tests should be described solely by name; describe more complex techniques in the Methods section.*
☒	☐	A description of all covariates tested
☐	☒	A description of any assumptions or corrections, such as tests of normality and adjustment for multiple comparisons
☐	☒	A full description of the statistical parameters including central tendency (e.g. means) or other basic estimates (e.g. regression coefficient) AND variation (e.g. standard deviation) or associated estimates of uncertainty (e.g. confidence intervals)
☒	☐	For null hypothesis testing, the test statistic (e.g. *F*, *t*, *r*) with confidence intervals, effect sizes, degrees of freedom and *P* value noted<br>*Give P values as exact values whenever suitable.*
☒	☐	For Bayesian analysis, information on the choice of priors and Markov chain Monte Carlo settings
☒	☐	For hierarchical and complex designs, identification of the appropriate level for tests and full reporting of outcomes
☒	☐	Estimates of effect sizes (e.g. Cohen's *d*, Pearson's *r*), indicating how they were calculated

Our web collection on statistics for biologists contains articles on many of the points above.

Software and code

Policy information about availability of computer code

Data collection	Microsoft Excel (version 16.64), GraphPad Prism (version 8)
Data analysis	Fiji (2.3.0), Clij (ver.), Graph Pad Prism 8 , Microsoft Excel (13), Adobe Illustrator (26.5) and Photoshop CSS (23.5.0), R package pheatmap (1.0.12), ggplot2 package for R (3.1.0), scanpy (version 1.3.1)

For manuscripts utilizing custom algorithms or software that are central to the research but not yet described in published literature, software must be made available to editors and reviewers. We strongly encourage code deposition in a community repository (e.g. GitHub). See the Nature Portfolio guidelines for submitting code & software for further information.

Data

Policy information about availability of data

All manuscripts must include a data availability statement. This statement should provide the following information, where applicable:

- Accession codes, unique identifiers, or web links for publicly available datasets
- A description of any restrictions on data availability
- For clinical datasets or third party data, please ensure that the statement adheres to our policy

The authors declare that all data supporting the findings of this study are available within the paper and its Supplementary information files, and no restrictions apply. The source data underlying Figs. 1 c, d, h; 2 c-i; 4 c, d, f; 5 d; 6 d, e; 8 b, d, and 9 b, d, e; and Supplementary Fig. 2 a, b; 3 c-h; 5 c, f, g; 6 f1, f2, g-k; 7 c; 8 a, b, e; 9 d-f; 10 c1, c2, h; 11; 12; 14 c-f; 17 b-c, and 18 b-c are provided as a Source Data file.
The next-generation sequencing data that supports the findings of the study have been deposited on Gene Expression Omnibus: the accession code is GSE146641 for the raw bulk RNA-seq data [https://www.ncbi.nlm.nih.gov/geo/query/acc.cgi?acc=GSE146641] and GSE174215 for single-cell RNA-Seq [https://www.ncbi.nlm.nih.gov/geo/query/acc.cgi?acc=GSE174215]; and there we also provide the uap workflow including all parameters and software used in the

supplement. RNA sequencing reads were aligned to the human genome using a publicly available dataset (UCSC Human Dec. 2013 GRCh38/hg38 Assembly).

Field-specific reporting

Please select the one below that is the best fit for your research. If you are not sure, read the appropriate sections before making your selection.

☒ Life sciences ☐ Behavioural & social sciences ☐ Ecological, evolutionary & environmental sciences

For a reference copy of the document with all sections, see nature.com/documents/nr-reporting-summary-flat.pdf

Life sciences study design

All studies must disclose on these points even when the disclosure is negative.

Sample size	Sample sizes were chosen based on previous experience using human and mouse organoids (see references 1-3 below) and pilot experiments. No statistical methods were used to predetermine sample size. Experiments were reliably reproduced using independent samples (organoid cultures or individual organoids) from separate differentiations. Sample sizes are depicted as number of organoids (n) or set of pooled organoids (s) per experiment (N). Unless stated otherwise N≥3 with n≥5 per N were used for histological analysis. Sample sizes for all experiments are reported in the supplemental data tables. Data are provided as a Source Data file.<br>References: (1) Völkner M, Kurth T, Schor J, Ebner LJA, Bardtke L, Kavak C, Hackermüller J, Karl MO. Mouse Retinal Organoid Growth and Maintenance in Longer-Term Culture. Front Cell Dev Biol. 2021; 9:645704. doi: 10.3389/fcell.2021.645704. (2) Völkner M, Pavlou M, Büning H, Michalakis S, Karl MO. Optimized Adeno-Associated Virus Vectors for Efficient Transduction of Human Retinal Organoids. Hum Gene Ther. 2021; 32(13-14):694-706. doi: 10.1089/hum.2020.321. (3) Völkner M, Zschätzsch M, Rostovskaya M, Overall RW, Busskamp V, Anastassiadis K, Karl MO. Retinal Organoids from Pluripotent Stem Cells Efficiently Recapitulate Retinogenesis. Stem Cell Reports. 2016; 6(4):525-538. doi: 10.1016/j.stemcr.2016.03.001.
Data exclusions	No data was excluded
Replication	All attempts at replication were successful. Findings were replicated based on independent experiments (N), as well as by using different types of analysis: histology, transcriptomics, and through mechanistic studies. We defined the number of independent experiments (N) based on individual batches (differentiations) of human retinal organoids (HROs). We indicate if different hiPSC lines were used for each N. We defined the number of HROs (n) analyzed per N. Thus, we analyzed and show HROs individually, and derive mean/median as well as statistics over n. We provide some data on inter-/intraorganoid variances, like in Supp. Fig. 2b, and we validated our findings with HROs derived from different N. Further, we validate the HT-HRO model in experiment across HROs from four different hiPSC lines each with ≥5 HROs (n) per individual experiment (N), i.e. at least 65 HROs per variable (Supp. Fig.11), where we derived mean and statistics over N.
Randomization	Organoids per experiment were randomly assigned to groups.
Blinding	Investigators were not blinded to group allocation; analysis was performed based on objective quantitative methods. Key experiments were validated in independent experiments. Selected data was analyzed by different investigators to validate findings. Blinding was not relevant to the characterization of the organoid system and the HT-induced pathology model, which was readily distinguishable from control.

Reporting for specific materials, systems and methods

We require information from authors about some types of materials, experimental systems and methods used in many studies. Here, indicate whether each material, system or method listed is relevant to your study. If you are not sure if a list item applies to your research, read the appropriate section before selecting a response.

Materials & experimental systems

n/a	Involved in the study	
☐	☒	Antibodies
☐	☒	Eukaryotic cell lines
☒	☐	Palaeontology and archaeology
☒	☐	Animals and other organisms
☒	☐	Human research participants
☒	☐	Clinical data
☒	☐	Dual use research of concern

Methods

n/a	Involved in the study	
☒	☐	ChIP-seq
☐	☒	Flow cytometry
☒	☐	MRI-based neuroimaging

Antibodies

Antibodies used	All antibody information are given in the order: Antigen; Target; Host species; Cat.no. and Vendor:<br>ARR3; cone photoreceptors; mouse; 7G6; a gift from P.R. MacLeisch, U Texas, Houston;<br>ARR3; Arrestin 3; Cone arrestin; cone photoreceptors; goat ; NBP1-37004 Novus;<br>ARR1; Arrestin beta 1; rod photoreceptors rabbit; PA1-731; Thermo Scientific;<br>ARL13B; primary cilia; inner segment mouse ab136649; Abcam;

ACTA2; mesoderm; mouse; A25538; Thermo Scientific;
ACTN; pan-actin (recognizes all actin isoforms); mouse; CLT9001; Cedarlane;
ARL13B; primary cilia, inner segment; rabbit; 177-11-1-AP; Cosmobio;
BRN3; ganglion cells; goat; sc31984; Santa Cruz;
BHLHE22; bHLHb5; amacrines; few bipolars; goat; sc-6046 Santa Cruz;
CALB1; calbindin; horizontals; cones in peripheral retina; mouse; 301 Swant
aCASP3; active caspase 3; cell death/apoptotic cells; rabbit 559565; BD
CRX; photoreceptors, photoreceptor precursors; rabbit; ; a gift from E. Tanaka, TU Dresden;
CYCS; mitochondria (inner segments); mouse; BLD-612302; Biolegend;
ELAVL3/4; amacrines, ganglion cells, horizontals; mouse; A-21271; Invitrogen;
EBF3; amacrines; ganglion cells; bipolars; mouse; H00253738-M06 Abnova;
GFAP; astrocytes, Müller glia endfeet (healthy) & Müller glia cell body (reactive); rat; 13-0300; Invitrogen;
GNAO1; ON-cone-BP; mouse MAB3074 Millipore;
GNAT1 transducin 1; rod photoreceptors; rabbit sc-389 Santa Cruz;
GNAT2 transducin 2; cone photoreceptors; rabbit PA5-24554 Thermo Scientific;
ISL1; ON-cone-BP; HC; few amacrines; mouse ab86473 Abcam;
ISL1; ON-cone-BP, HC, few amacrines; mouse; 39.4D5; DSHB;
KI67; proliferating cells; mouse; 550609; BD;
mito; human mitochondria (inner segments); mouse; MAB1273; EMD Millipore;
NRL; rod photoreceptors; goat; AF2945; R&D;
OCT4; pluripotency; mouse; 560253; BD;
OTX2; Photoreceptors, bipolars; goat; BAF1979; R&D;
ONECUT2; horizontals; amacrines; sheep AF6295 R&D;
OPN1LW/MW; long & medium wave sensitive L/M-cones; rabbit AB5406 EMD Millipore;
OPN1SW; short wave sensitive S-cones; goat sc-14363 Santa Cruz;
PRKCA PKCa; rod bipolars; mouse; 05-154 EMD Millipore;
PRKCA PKCa; rod bipolars; rabbit; sc-209 Santa Cruz;
PRPH2; peripherin-2; outer segment; rabbit; 18109-1-AP; Proteintech;
PVALB; parvalbumin; amacrines; mouse; P3089; Sigma;
PAX6; progenitors, MG, HC, AC, GC; rabbit; 901301; Biolegend;
PHH3; proliferating cells (mitosis); rat; NB600-1168; Novus;
PRKCA; rod bipolars; mouse; 05-154; EMD Millipore;
RCVRN; Photoreceptors, some BPs, some GCs; rabbit; AB5585; EMD Millipore;
RCVRN-FITC Recoverin; Photoreceptors, some BPs, some GCs; rabbit; 1251-05041; Assay Pro
RHO; rod photoreceptors; mouse; O4886; Sigma-Aldrich;
RLBP1; Müller glia; mouse; MA1-813; Thermo Scientific;
RBFOX3; amacrines; ganglion cells; horizontals; mouse MAB378 Millipore ;
ROM1; outer segment; rabbit 21984-1-AP Proteintech;
SLC1A3; Müller glia; mouse; 130-095-822; Miltneyi Biotec;
SOX17; endoderm; mouse; ab84990; Abcam;
SOX2; pluripotency; mouse; 560291; BD;
SOX9; Müller glia, progenitors; rabbit; HPA001758; Sigma;
SOX9; Müller glia; progenitors; goat AF3076 R&D;
SOX9; Müller glia; progenitors; rabbit ab185967 Abcam;
SSEA4; pluripotency; mouse; 561156; BD;
Tra-1-60; pluripotency; mouse; 560850; BD;
TUBB3; ectoderm; rabbit; A25538; Thermo Scientific;
VSX2; bipolars, progenitors, Müller glia; sheep; X1180P; Exalpha;

Validation

All antibodies used in this study work well for immunohistochemistry in mouse and/or human samples. The expression pattern for each antibody matches previous reports in the literature.

Eukaryotic cell lines

Policy information about cell lines

Cell line source(s)

One newly-generated hiPSC line CRTD1 (https://hpscreg.eu/cell-line/CRTDi004-A), and three previously published ones 5A (see methods for reference), IMR90 (iPS(IMR90)-4, WiCell; https://hpscreg.eu/cell-line/WISCi004-B), and CRTD2 (https://hpscreg.eu/cell-line/CRTDi003-A) were used (details in method section).
Cell lines we generated will be made available to researchers upon reasonable request and are registered at https://hpscreg.eu as indicasted above.

Authentication

None of the cell lines used were authenticated.

Mycoplasma contamination

All cell lines used were tested frequently negative for mycoplasma and were negative.

Commonly misidentified lines
(See ICLAC register)

None

Flow Cytometry

Plots

Confirm that:

☒ The axis labels state the marker and fluorochrome used (e.g. CD4-FITC).

☒ The axis scales are clearly visible. Include numbers along axes only for bottom left plot of group (a 'group' is an analysis of identical markers).

☒ All plots are contour plots with outliers or pseudocolor plots.

☒ A numerical value for number of cells or percentage (with statistics) is provided.

Methodology

Sample preparation

Fig.S1D,G: Pluripotency marker characterization of hiPSC.

Flow cytometric analysis of pluripotency:
Alexa Flour 488 anti-OCT3/4, PE anti-SOX2, V450-SSEA-4, and Alexa Flour 647 anti Tra-1-60 were used. All antibodies were obtained from BD Pharmingen (Table S8) and used according to the manufacturer's recommendations.

Fig.2E, S6A-J: Cell composition analysis by flow cytometry of HROs after 10 days of HT treatment and control.
Fig.2F: Flow cytometry of calcein live-dye staining.
Fig.S10: Flow-cytometry analysis of the MG (SOX9) population showed an increase in the forward scatter (FSC), a proxy for cell size, in HT-treated HROs.

Flow cytometry analysis of HRO cell composition:
Immunostaining for retinal cell-type analysis of dissociated HRO cells:
HRO samples were pooled (6–9 HROs per sample, at D210–220) for each experiment and variable (control and HT-treated, 10+/-1 days of treatment), and dissociated using the Papain Dissociation System (Worthington Industries) according to manufacturer's instructions. Briefly, pooled samples were incubated in 500 µl of papain (20 U/ml, 2 h, 37℃) in an orbital shaker incubator (ES 20/60, Biosan, 100 rpm), followed by manual trituration through a fire-polished glass pipette. Dissociated cells were re-suspended in PBS with 0.04% BSA and subsequently fixed with PFA (1% in PBS, 15 min, RT) on an orbital shaker (0.4 g), washed once with PBS, and centrifuged (10 min, 480 g). Cell suspension was treated with a blocking agent (10% FBS, 0.1% Triton in PBS) for 10 min on the orbital shaker. Subsequently, the cell suspension was divided into separate tubes, for the different sets of antibodies detecting RCVRN-FITC and ARR3, and SOX9 and ARR3 (see Table S8). ARR3 and SOX9 primary antibodies were detected by fluorescently conjugated secondary antibodies (1:1000; AlexaFluor 647 anti-mouse and AlexaFlour 488 anti-rabbit, Dianova). Cell suspensions were protected from light and incubated with antibodies for 30 min on the orbital shaker (200 rpm); as indicated, secondary antibodies were added halfway through incubation time. After washing with PBS, cells were centrifuged (6 min, 600 g), resuspended in PBS with 0.04% BSA, and filtered through a Flowmi cell strainer (40 µm, BelArt, SP Scienceware). N=3 independent experiments from 2 hiPSC lines: 5A (N=2) and CRTD1 (N=1). Within each experiment, 2–3 technical replicate samples (each 6–9 HROs pooled) for both variables (CTRL and HT-treated) were collected and independently processed (dissociated, stained, and analyzed using flow cytometry (see below)). To validate antibodies, we performed immunostaining (Fig.S6B) and imaging flow cytometry (method see below, Fig.2E; S6C). 30–40 +/- 103 cells per sample were analyzed.

Flow cytometry-based live/dead discrimination of dissociated cells:
Cells were dissociated using papain as described above, resuspended in PBS with 0.04% BSA and filtered through a Flowmi cell strainer (40 µm). Live-dead staining was performed using the LIVE/DEAD™ Viability/Cytotoxicity Kit (Thermo Fisher) according to the manufacturer's instructions. Briefly, cells were incubated with a final concentration of 8 µM ethidium homodimer-1 (2 mM in DMSO/H2O 1:4) and 0.1 µM calcein AM (50 µM in DMSO) for 15 min at RT and then analyzed by flow cytometry. 30–40 +/- 103 cells per sample were analyzed (4 samples, 2 HROs/sample, N=1 independent experiment, 1 hiPSC line (CRTD1)).

Flow-cytometry analysis of dissociated HRO cells:
The immunostained or live-dead kit-labeled dissociated HRO cells, were analyzed using flow cytometry on a BD FACS Aria III cell sorter (100 µm nozzle, 20 psi sheath pressure). For cell-type analysis only, the nuclear dye DAPI was applied shortly before analysis, to enable distinction between nucleated cells and membranous debris. Fluorescence of DAPI, GFP/Cy2/A488, and Cy5/A647 was measured at 405 nm (bandpass filter (BP) 450/40 nm), 488 nm (BP 530/30 nm), and 633 nm (BP 660/20 nm), respectively. For live-dead discrimination, fluorescence of calcein (viable cells) and ethidium homodimer-1 (dead cells) was measured at 488 nm excitation using BP 530/30nm and BP 610/20nm filters, respectively. Data analysis was performed with FlowJo software (FlowJo, LLC), v.10.5.3. As a proxy for MG hypertrophy (defined as cell size), the median forward-scattered light area (FSC-A) of the SOX9+ cell population was determined, which is proportional to cell surface area or size. For cell-type and MG-hypertrophy analysis, 1237–21024 (median: 7279) SOX9+ cells per sample were included. Each sample consisted of 6–9 pooled HROs. 2–3 technical replicates (samples) per independent experiment (N) and variable. N=3 independent experiments from 2 hiPSC lines: 5A (N=2) and CRTD1 (N=1). Further, to determine if any cells are co-labeled for MG and photoreceptor markers, single cells were analyzed for double positivity of SOX9 and ARR3 using the BD FACSDiva software: 32000–40000 cells within all events were used for gating and SOX9+ARR3+ events ranged between 8 and 255. To validate that the antibodies differentially immunolabeled HRO-derived cells, we also performed imaging flow cytometry using an Amnis ImageStream X Mk II imaging flow cytometer (Luminex) after gating cells for nucleated singlets. For sample excitation, the following laser intensities were used in separate channels: DAPI: 405 nm laser, 20 mW; FITC/Alexa488: 488 nm, 20 mW; Alexa647: 642 nm, 150 mW; SSC: 78 5nm, 4.69 mW. The brightfield image was recorded in two channels with

LED intensities of 30.84 and 32.74 mW. Image analysis was performed with IDEAS Application v.6.2.64.0.

Instrument	BD FACS Aria III cell sorter
Software	FlowJo software (FlowJo, LLC), version 10.5.3
Cell population abundance	30.000-40.000 cells analysed
Gating strategy	Pluripotency marker analysis of hiPSC (Suppl. Fig. 1 d, g): 1. Gating on FCS-A and SSC-A performed on live cells to exclude cell debris and aggregates. 2. Gating on SSC-H and SSC-W, and FSC-H and FSC-W to exclude doublets. 3. Gating on Alexa Fluor 488 anti-Oct3/4, PE anti-Sox2, V450-SSEA-4, and Alexa Fluor 647 anti-Tra-1-60 to determine pluripotency marker expression was performed per manufacturer's recommendations. For each marker gates were set in comparison with the non-stained control. Gating is shown in Supplementary Figure 1 d, g.<br><br>HRO cell composition analysis (Suppl. Fig. 6 d-j; Fig.1 e1, e2, f) and Müller glia cell hypertrophy analysis (Suppl. Fig. 10 h, h1-h3): 1. Gating on FCS-A and SSC-A performed on fixed cells to exclude cell debris and aggregates. 3. Further, only nucleated (DAPI-positive gated) cells were included. 4. Two marker sets were each analyzed on separately prepared and analyzed samples with the following gating: Marker set 1) ARR3-A647 and SOX9-A488 to select for cone and Müller glia cells, respectively. Marker set 2) RCVRN—FITC and ARR3-A647 to select for total photoreceptors and cone cells, respectively. Gating on combined SOX9-A488 and ARR3-A647 was performed to determine if any cells are positive for both markers. For each marker, gates were set in comparison with the non-stained control. To establish markers, gate boundaries were determined on samples stained for each marker separately, and in combinations. Final gating strategy is shown in Supplementary Figure 6 d, e1, e2, e3, i, j.<br><br>Cell viability analysis: Steps 1-2 as above for pluripotency markers analysis. 3. Gating for Calcein live-dye and ethidium homodimer to determine cell viability was performed per manufacturer's recommendations. For each marker gates were set in comparison with the non-stained control. Gating is shown in Supplementary Figure 6 d, h.

☒ Tick this box to confirm that a figure exemplifying the gating strategy is provided in the Supplementary Information.
